# Taxonomic Status of the *Bemisia tabaci* Complex (Hemiptera: Aleyrodidae) and Reassessment of the Number of Its Constituent Species

**DOI:** 10.1371/journal.pone.0063817

**Published:** 2013-05-13

**Authors:** Wonhoon Lee, Jongsun Park, Gwan-Seok Lee, Seunghwan Lee, Shin-ichi Akimoto

**Affiliations:** 1 Laboratory of Systematic Entomology, Department of Ecology and Systematics, Graduate School of Agriculture, Hokkaido University, Kita-ku, Sapporo, Japan; 2 Stronghold Innovation Center, Gocheok-dong, Guro-gu, Seoul, Korea; 3 Crop Protection Division, National Academy of Agricultural Science, RDA, Gyongi*-*do, Korea; 4 Insect Biosystematics Laboratory, Research Institute for Agricultural and Life Sciences, Seoul National University, Seoul, Korea; University of Arkansas, United States of America

## Abstract

*Bemisia tabaci* (Hemiptera: Aleyrodidae) is one of the most important insect pests in the world. In the present study, the taxonomic status of *B. tabaci* and the number of species composing the *B. tabaci* complex were determined based on 1059 *COI* sequences of *B. tabaci* and 509 *COI* sequences of 153 hemipteran species. The genetic divergence within *B. tabaci* was conspicuously higher (on average, 11.1%) than interspecific genetic divergence within the respective genera of the 153 species (on average, 6.5%). This result indicates that *B. tabaci* is composed of multiple species that may belong to different genera or subfamilies. A phylogenetic tree constructed based on 212 *COI* sequences without duplications revealed that the *B. tabaci* complex is composed of a total of 31 putative species, including a new species, *JpL*. However, genetic divergence within six species (*Asia II 1*, *Asia II 7*, *Australia*, *Mediterranean*, *New World*, and *Sub Saharan Africa 1*) was higher than 3.5%, which has been used as a threshold of species boundaries within the *B. tabaci* complex. These results suggest that it is necessary to increase the threshold for species boundaries up to 4% to distinguish the constituent species in the *B. tabaci* complex.

## Introduction

The sweet potato whitefly, *Bemisia tabaci* (Gennadius, 1889) (Hemiptera: Aleyrodidae), is one of the most important agricultural pests in the world [1]–[3]. This species attacks several host plants including ornamental and vegetable plants, grain legumes, and cotton by ingesting the phloem sap [4] and transmits begomoviruses (Geminiviridae) [5], [6], inducing a large amount of economic damage [3]. *B. tabaci* is a complex of at least 24 morphologically indistinguishable species [3]. These species have been mainly distinguished by molecular methods because of difficulties in detecting morphological and biological differences among the species [3], [7], [8]. Since the 1990s, various molecular methods such as allozymes, random amplified polymorphic DNA, microsatellites, mitochondrial genes, and nuclear genes have been used for defining several groups in this complex [3]. Currently, mitochondrial *cytochrome oxidase subunit I* (*COI*) gene is primarily used to define several species of the *B. tabaci* complex [9].

Until 2010, large genetic divergence within *B. tabaci* had posed the question of whether *B. tabaci* was one biological species composed of several biotypes or a complex of biological species [10]. Dinsdale et al. [9] addressed this problem and reported that 24 constituent species of *B. tabaci* were distinguished by a threshold of 3.5% difference based on 454 *COI* sequences and biological characters. As a result, many researchers have distinguished the constituent species in the *B. tabaci* complex based on the 3.5% genetic boundary [11]–[13]. However, the 3.5% boundary has not been compared with species boundaries of other groups regarding the same position of *COI* sequence. Thus, it is required to confirm the ground for the 3.5% boundary in the *B. tabaci* complex using much more sequence data. Until now, it is still unresolved whether the *B. tabaci* complex is monophyletic, consisting of cryptic species, or is polyphyletic, including species of different genera, subfamilies, and families. This question is raised because genetic divergence in *COI* sequences within the *B. tabaci* complex (range, 0–34%) [9] is much higher than that observed between congeneric species in other hemipteran groups such as aphids (range, 0–11%) [14].

Recently, new species of the *B. tabaci* complex have been reported based on the 3.5% boundary. For example, Hu et al. [13] reported four new species (*Asia II 9*, *Asia II 10*, *Asia III*, and *China 3*), and then two new species (*Asia I-India* and *New World 2*) were later reported by Chowda-Reddy et al. [11] and Alemandri et al. [12], respectively. As a result, a total of 30 species have been reported in the *B. tabaci* complex. However, this estimate is still uncertain because not all of the *COI* sequences of *B. tabaci* were included in these studies (i.e., Dinsdale et al. [9], Chowda-Reddy et al. [11], Alemandri et al. [12], and Hu et al. [13]). For example, Ueda et al. [15] reported one putative new species, *JpL*, of the *B. tabaci* complex; however, *COI* sequences of the *JpL* species have not been considered in the subsequent calculations of the number of species in the *B. tabaci* complex [9].

A number of species concepts have been proposed to date, and the usefulness and applicability of respective species concepts has been critically discussed [16]. A species concept based on genetic distance has often been criticized because genetic distance does not always reflect the existence of pre- or post-mating isolation [17]. This criterion of the biological species concept (BSC) [18] has been applied to diverse taxonomic groups with different genetic systems and life histories. However, unusually, the delimitations of the species comprising the *B. tabaci* complex have been based on a discontinuous distribution of genetic distances in genetic markers without considering biological data. Currently, most researchers have followed ‘phylogenetic’ species concept [9], in which species boundaries are constructed with phylogenetic analyses and pairwise comparisons of genetic distances. Thus, in the present study, we consistently use “species” as a group that is discontinuous from other such groups in terms of genetic distance.

In this study, first, we examined the taxonomic status of *B. tabaci* by calculating genetic divergences among a larger number of *COI* sequences collected from databases. As a comparison, genetic divergence among 509 *COI* sequences of 153 hemipteran species was calculated according to three taxonomic levels (within species, between congeneric species, and between genera in the same family), and the results were compared with genetic divergence among 1059 *COI* sequences of *B. tabaci*. Second, a phylogenetic tree was constructed based on 212 *COI* sequences of *B. tabaci* and reassessed the number of species composing the *B. tabaci* complex. In this re-analysis, we revised the threshold genetic distance that most appropriately represents species boundaries within the *B. tabaci* complex by observing the distribution of genetic divergences among the *COI* sequences of the *B. tabaci* complex. As a control group to the *B. tabaci* complex, genetic divergence of 34 aphid species was calculated according the three taxonomic levels. Third, to examine the advantage and characteristics of the *COI* gene as a genetic marker, we compared patterns of genetic divergences among the *COI* gene and ten other mitochondrial genes by using 20 hemipteran mitochondrial genomes.

## Results

### Genetic Divergence at Three Taxonomic Levels

**Figure 1:**
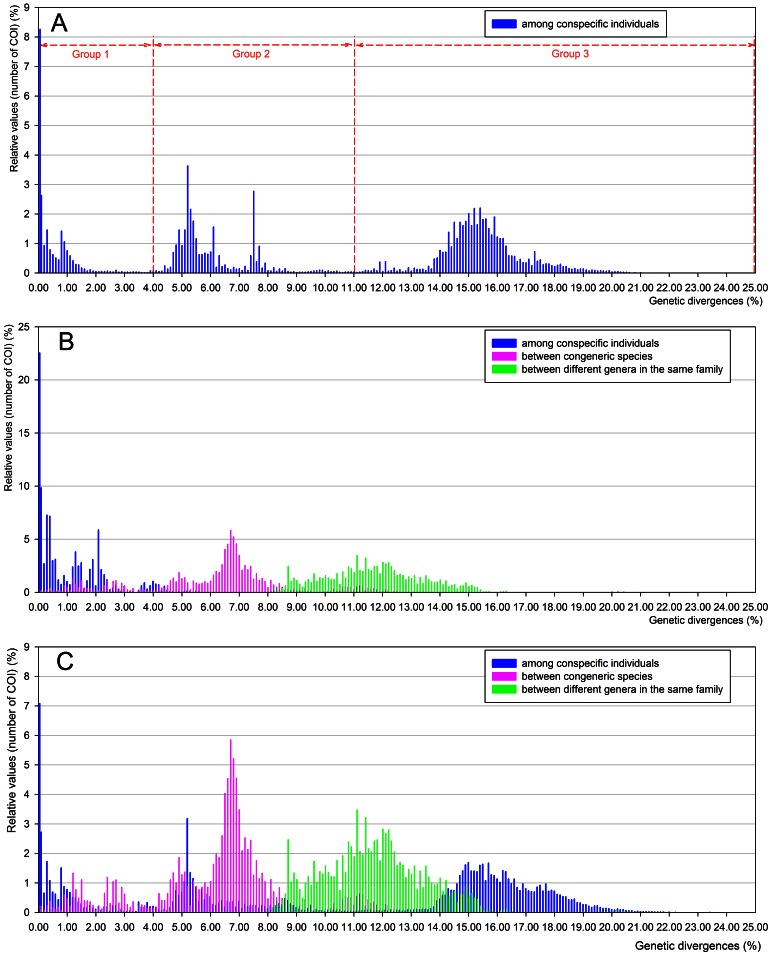
Distribution of genetic divergence estimate for *COI* sequences according to taxonomic levels. (A) *Bemisia tabaci* dataset consisting of 1059 *COI* sequences of *B. tabaci*, (B) hemipteran species dataset consisting of 509 *COI* sequences of 153 hemipteran species, and (C) combined dataset consisting of 1568 *COI* sequences of 153 hemipteran species and *B. tabaci*.

In the *B. tabaci* dataset, genetic divergence was detected among the *COI* sequences, ranging from 0% to 24.1% with an average of 11.1% (Table 1). Genetic divergence among *B. tabaci* individuals was categorized into three separate groups based on their frequency distributions (Figure 1A): group 1 (divergence ranging from 0–4%), group 2 (range, 4–11%), and group 3 (range, 11–25%). Dinsdale et al. [9] reported that the genetic divergences among 198 *COI* sequences of *B. tabaci* and four sequences of *B. atriplex*, *B. afer*, and *B. subdecipiens* ranged from 0% to 34%; the range was subsequently separated into four groups: group 1 (range, 0–3%), group 2 (range, 3.5–11%), group 3 (range, 11–26%), and group 4 (range, 26–34%). Because our analysis excluded the four sequences of *B. atriplex*, *B. afer*, and *B. subdecipiens*, the group 4 in Dinsdale et al. [9] was not used.

**Table 1:** Genetic divergence estimates of the three datasets at three taxonomic levels.

	Within species			Within genus and between species			Within family and between genera		
	Avg.(%)^a^	Min.(%)^b^–Max.(%)^c^	N.C.^d^	Avg.(%)	Min.(%)–Max.(%)	N.C.	Avg.(%)	Min.(%)–Max.(%)	N.C.
*Bemisia tabaci*	11.1	0.0*–*24.1	207046	·	·	·	·	·	·
hemipteran species	1.0	0.0*–*4.9	6474	6.5	0.0–28.1	12004	12.1	5.8*–*26.9	27928
*B. tabaci+*hemipteranspecies	10.7	0.0–24.1	213171	6.5	0.0–28.1	13629	12.9	5.8–26.9	28566

a Average,

b Minimum,

c Maximum, and ^d^Number of comparisons.

The hemipteran species dataset exhibited a different tendency from the *B. tabaci* dataset in the average genetic divergence within species (Table 1); the average genetic divergence increased as the taxonomic level rose (Figure 1B). The combined dataset (*B. tabaci*+hemipteran species) also exhibited variation in genetic divergence at the three taxonomic levels (Table 1). However, in this case, they did not increase with rising taxonomic levels (Figure 1C), and the average genetic divergence among conspecific individuals (10.7%; range, 0–24.1%) was higher than the average genetic divergence between congeneric species (6.5%; range, 0–28.1%).

As a control to the *B. tabaci* complex, we used aphids which are closely related to the genus *Bemisia* in Hemiptera and quantified the pattern of genetic divergences using 47 *COI* sequences of 34 aphid species. The aphid group exhibited average genetic divergences of 0.3% (range, 0–1.5%) among individuals of one species, 5.7% (range, 0.1–11.7%) between different species belonging to one genus, and 12.8% (range, 5.2–18.9%) between different genera belonging to one subfamily.

### Defining the Number of Species of the *B. tabaci* Complex

**Figure 2:**
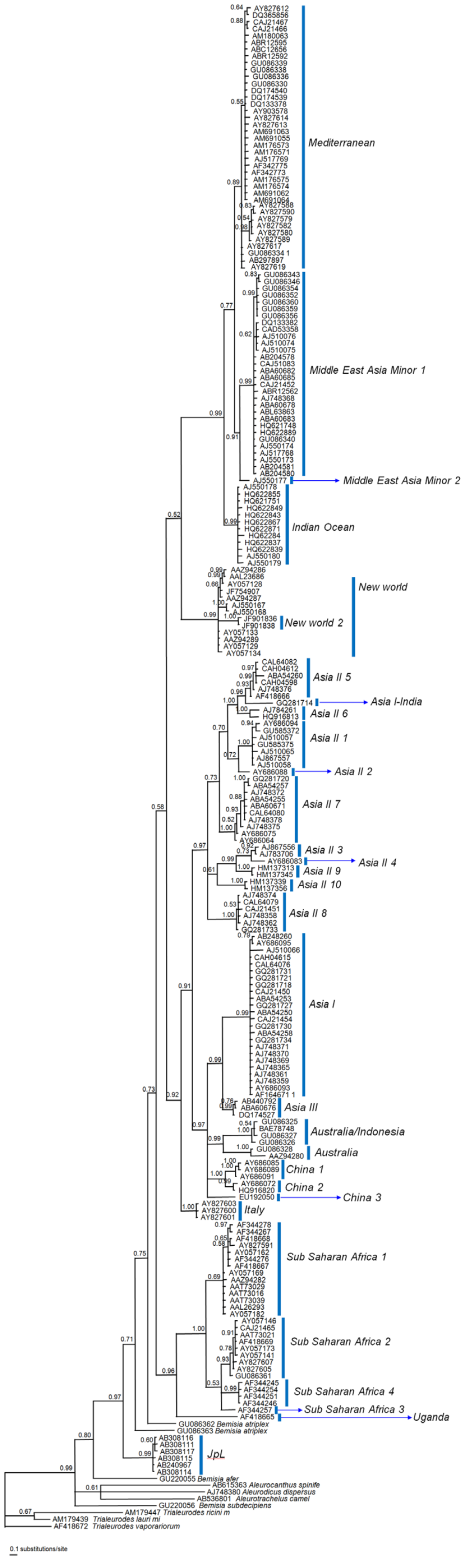
Phylogenetic tree based on 212 *COI* sequences of *Bemisia tabaci* estimated using Bayesian inference analysis. Posterior probability is shown above branches.

The phylogenetic tree constructed from the 222 *COI* sequences of *B. tabaci*+ten other aleyrodide species revealed that the *B. tabaci* sequences were separated into 31 groups (Figure 2). These groups were named based on five previous studies [9], [11]–[13], [15]. The phylogenetic tree was similar in topology to those of the previously reported phylogenetic trees [3], [13]. For example, four species (*Indian Ocean*, *Mediterranean*, *Middle East Asia Minor 1*, and *Middle East Asia Minor 2*) were clustered with 99% posterior probability. In addition, eight species (*Asia II 1*, *Asia II 2*, *Asia II 3*, *Asia II 4*, *Asia II 5*, *Asia II 6*, *Asia II 7*, and *Asia II 8*) and seven species (*Asia I*, *Asia III*, *Australia/Indonesia*, *Australia*, *China 1*, *China 2*, and *China 3*) were clustered with 97% posterior probability, respectively. However, some species showed different relationships from the previous studies. Here, *New World* was clustered with four species (*Indian Ocean*, *Mediterranean*, *Middle East Asia Minor 1*, and *Middle East Asia Minor 2*), whereas it was clustered with *Bemisia atriplex* in Dinsdale et al. [9]. In addition, *Asia I-India* was clustered with *Asia II 5* with 96% posterior probability, whereas it was clustered with *Asia I* in Chowda-Reddy et al. [11]. Additionally, *New World 2* came into *New World* in the present study; however, it was separated from *New world* in Alemdandri et al. [12]. Among the 31 species, *JpL* has not been rerpoted in other molecular studies conducted since the publication of Ueda et al. [15]. For the first time, we observed that *JpL* species was separated from the other 30 species in the phylogenetic tree.

**Figure 3:**
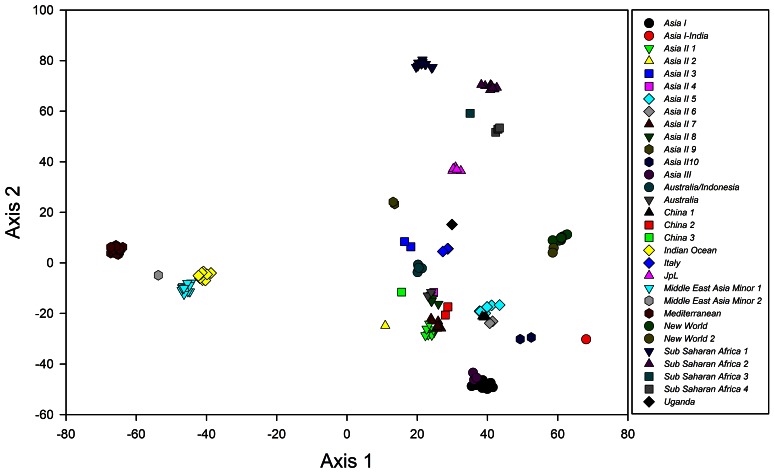
Results of the canonical discriminant analysis based on the 22336 pairwise genetic distances for demonstrating the relationship among the 31 species belonging to the *Bemisia tabaci* complex.

Figure 3 indicates the first two axes of a canonical discriminant analysis (CDA) for the 31 species of *B. tabaci* based on a matrix of pairwise genetic distances. The first axis in the diagram clearly separates the 31 species assemblages into two groups, with the plots from four species (*Indian Ocean*, *Mediterranean*, *Middle East Asia Minor 1*, and *Middle East Asia Minor 2*) having negative scores, and the plots from the remaining 27 species all having positive scores. The second axis, coupled with the first axis, successfully separated the sampling units into the 27 species. The validity of this classification was tested with a leave-one-out cross-validation method, in which CDA was conducted using all the sequence data excepting one. Then, we tested into which group the excepted sequence was categorized based on the discriminant criteria, and this test was repeated for every sequence by excluding the sequences one by one. The result of this reclassification revealed that the proportion of correct classification was 100%, indicating that the 31 species are clearly distinct (Table 2). Thus, CDA corroborated the 31 groups revealed by the phylogenetic analysis.

**Table 2:** Classification table, showing the reclassification of the original visits into groups, and the success and misclassification rates of the CDA.

Original group	Classified into groups ()																															Total	% correct
	1	2	3	4	5	6	7	8	9	10	11	12	13	14	15	16	17	18	19	20	21	22	23	24	25	26	27	28	29	30	31		
*Asia I* (1)	24																															24	100%
*Asia I-India* (2)		1																														1	100%
*Asia II 1* (3)			7																													7	100%
*Asia II 2* (4)				1																												1	100%
*Asia II 3* (5)					2																											2	100%
*Asia II 4* (6)						1																										1	100%
*Asia II 5* (7)							6																									6	100%
*Asia II 6* (8)								2																								2	100%
*Asia II 7* (9)									10																							10	100%
*Asia II 8* (10)										6																						6	100%
*Asia II 9* (11)											2																					2	100%
*Asia II 10* (12)												2																				2	100%
*Asia III* (13)													3																			3	100%
*Australia/Indonesia* (14)														4																		4	100%
*Australia* (15)															2																	2	100%
*China 1* (16)																3																3	100%
*China 2* (17)																	2															2	100%
*China 3* (18)																		1														1	100%
*Indian Ocean* (19)																			12													12	100%
*Italy* (20)																				3												3	100%
*JpL* (21)																					6											6	100%
*Middle East Asia Minor 1* (22)																						30										30	100%
*Middle East Asia Minor 2* (23)																							1									1	100%
*Mediterranean*(24)																								39								39	100%
*New World* (25)																									11							11	100%
*New World 2* (26)																										2						2	100%
*Sub Saharan Africa 1* (27)																											14					14	100%
*Sub Saharan Africa 2* (28)																												9				9	100%
*Sub Saharan Africa 3* (29)																													1			1	100%
*Sub Saharan Africa 4* (30)																														4		4	100%
*Uganda* (31)																															1	1	100%
Total	24	1	7	1	2	1	6	2	10	6	2	2	3	4	2	3	2	1	12	3	6	30	1	39	11	2	14	9	1	4	1	212	100%

### Comparing Intra- and Inter-specific Genetic Divergences

**Figure 4:**
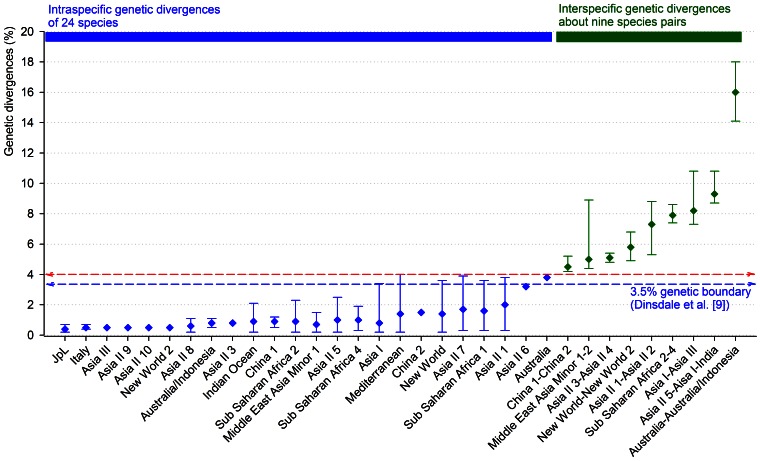
Comparison of intraspecific genetic divergences in 24 species and interspecific genetic divergences in nine species pairs.

Intraspecific genetic divergence of 24 species with two or more different sequences and the interspecific genetic divergence between pairs of closely related species in the *B. tabaci* complex were analyzed using the *p*-distance and K2P distance models (Figure 4; Tables S1 and S2). Nine pairs of species were selected based on the result of the above-mentioned phylogenetic analysis. Overall, genetic divergence estimates from the K2P distance model were higher than those from the *p*-distance model (Tables S1 and S2). In this study, we focused on the results obtained with the K2P distance model because the 3.5% boundary was calculated based on the K2P distance model [9], [19].

Intraspecific genetic divergence varied among the 24 species, ranging from 0.2% to 4.0% (Figure 4; Table S1). Among the 24 species, *JpL* demonstrated the lowest average genetic divergence, 0.4% (range, 0.2*–*0.7%), whereas *Australia* showed the highest average genetic divergence, 3.8%. Most of the species showed average genetic divergences less than 2.0%, except for two species, *Asia II 6* (with an average of 3.1%) and *Australia* (with an average of 3.8%). However, six species (*Asia II 1*, *Asia II 7*, *Australia*, *Mediterranean, New World*, and *Sub Saharan Africa 1*) had the maximum genetic divergences that were higher than the genetic boundary of 3.5%. In particular, *Mediterranean* showed genetic divergences ranging from 0.2% to 4.0%.

**Figure 5:**
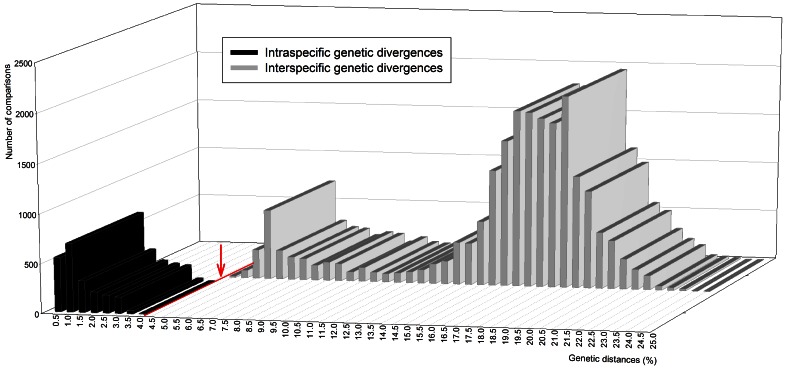
Distribution of genetic divergences based on the Kimura-2 parameter analysis for *COI* sequences according to two taxonomic levels.

Interspecific genetic divergence estimates also varied among the nine pairs of closely related species (Figure 4; Table S2). Among them, *Australia−Australia/Indonesia* showed the largest average genetic divergence, 16.0% (range, 14.1–18.0%), while *China 1−China 2* showed the lowest average divergence, 4.5% (range, 4.2–5.2%). The genetic divergences between all the combinations of the nine pairs were always higher than the 3.5% boundary (Figure 4; Table S2). From 22366 pairwise genetic distances for the 212 *COI* sequences, we observed that the 31 species revealed an average intraspecific genetic divergence of 1.2% (range 0.2–3.9%) and an average interspecific genetic divergence of 15.7% (range, 4.2–24.1%) (Figure 5).

### Comparing Genetic Divergences between *COI* and Ten Mitochondrial Genes

**Figure 6:**
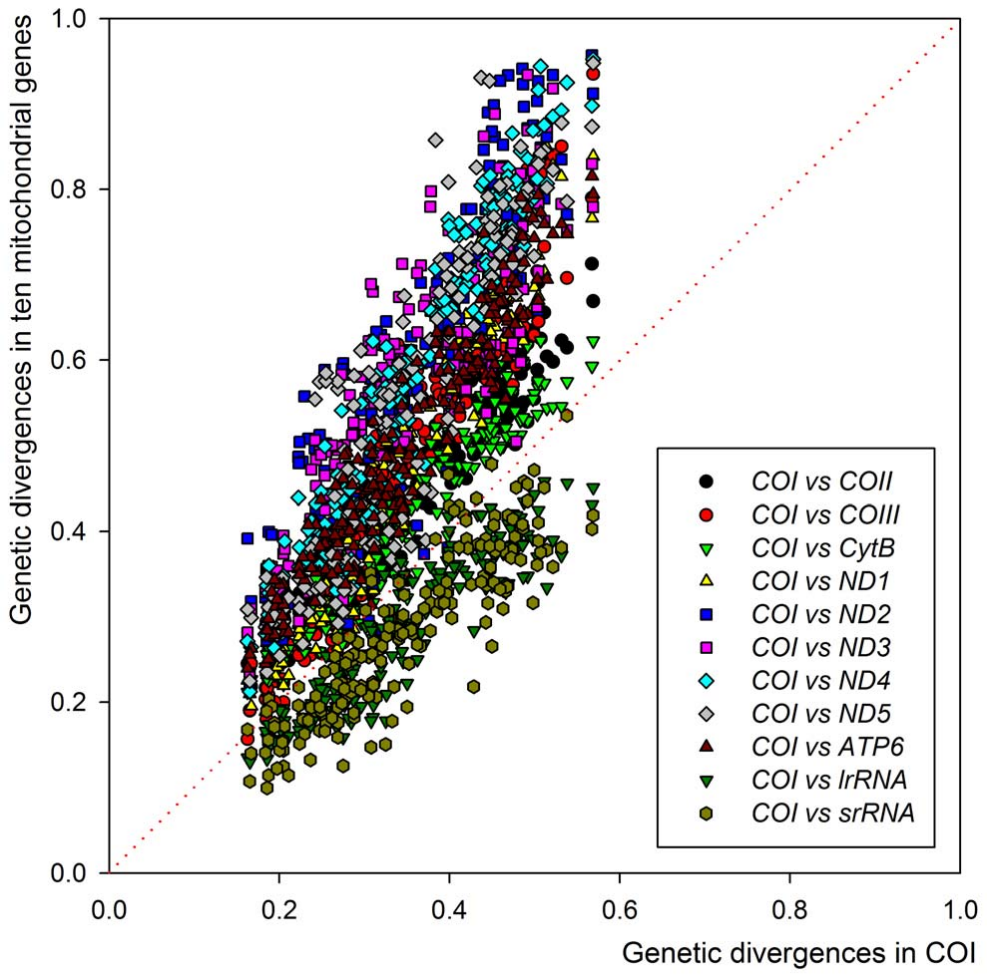
Relationships between the genetic distance in *COI* and that in another mitochondrial gene (*ATP6*, *COII*, *CytB*, *ND1*, *ND2*, *ND3*, *ND4*, *ND5*, *lrRNA*, and *srRNA*) for the same pair of hemipertan species.

A total of 220 sequences of eleven mitochondrial genes were examined by using 20 hemipteran mitochondrial genomes, including *B. tabaci* (NC 006279) (Table S3). Comparisons of the K2P distances among the eleven genes revealed that eight genes (*ATP6*, *COII*, *CytB*, *ND1*, *ND2*, *ND3*, *ND4*, and *ND5*) showed higher pairwise divergences, whereas two genes (*lrRNA* and *srRNA*) showed lower pairwise divergences than did *COI* (Figure 6). Wilcoxon signed rank tests also indicated that *COI* had lower genetic distances among all the species than did the eight genes, but higher genetic distances than did *lrRNA* and *srRNA* (Table 3). For all mitochondrial genes but *COI*, the genetic divergences for each gene exhibited a linear relationship to those for *COI*, suggesting that mitochondrial genes with a higher evolutionary rate could be used as a genetic marker as well as *COI* in the *B. tabaci* complex.

**Table 3:** Wilcoxon signed rank tests of interspecific difference among loci.

Locus pairs		Relative Ranks, *n*, *P*-value	Result
W+	W−		
*COI*	*ATP6*	W+ = 0, W− = 96, n = 190, *P*≤0.000	*COI<ATP6*
*COI*	*COII*	W+ = 1, W− = 96, *n = *190, *P*≤0.000	*COI<COII*
*COI*	*CytB*	W+ = 10, W− = 99, n = 190, *P*≤0.000	*COI<CytB*
*COI*	*ND1*	W+ = 11, W− = 99, n = 190, *P*≤0.000	*COI<ND1*
*COI*	*ND2*	W+ = 4, W− = 97, n = 190, *P*≤0.000	*COI<ND2*
*COI*	*ND3*	W+ = 0, W− = 96, n = 190, *P*≤0.000	*COI<ND3*
*COI*	*ND4*	W+ = 0, W− = 96, n = 190, *P*≤0.000	*COI<ND4*
*COI*	*ND5*	W+ = 0, W− = 96, n = 190, *P*≤0.000	*COI<ND5*
*COI*	*lrRNA*	W+ = 98, W− = 16, n = 190, *P*≤0.000	*COI>lrRNA*
*COI*	*srRNA*	W+ = 102, W− = 24, n = 190, *P*≤0.000	*COI>srRNA*

*n* is the number of comparison pairs. *P*-value is one sided probability of divergence rates being equal. *P*-values less than 0.05 were considered significant and interpreted to reflect significant differences in observed rates of divergences.

## Discussion

### Taxonomic Status of the *B. tabaci* Complex

The results of our analyses indicate that the taxonomic status of *B. tabaci* is peculiar, compared to that of other hemipteran species. Among the three datasets examined, the pattern of genetic distances in the hemipteran species dataset (Figure 1B) was similar to the pattern reported in DNA barcode studies with the superclass Hexapoda [20]. Barcode studies have revealed that genetic divergence estimates generally increased from low taxonomic levels (e.g., conspecific individuals and congeneric species) to higher taxonomic levels (e.g., confamilial species) in several orders such as Hemiptera [14], Lepidoptera [21], Diptera [22], and Hymenoptera [23]. However, in the combined dataset (Figure 1C), such an increase in genetic divergences across taxonomic levels was not observed. This result may be mainly due to the broad range of intraspecific genetic divergences observed only in *B. tabaci*. The distribution pattern of genetic divergences in the combined dataset has not been documented in Hemiptera or in other hexapod orders. This result corroborates the possibility that the *B. tabaci* complex consists of several species that may belong to different genera or subfamilies with respect to genetic divergence.

Despite the large genetic divergence, there was no clear evidence for polyphyly in the *B. tabaci* complex. The phylogeny obtained indicated that other *Bemisia* species do not cluster with any species of the *B. tabaci* complex. Although the resolution of *Bemisia* species, including *B. tabaci*, was not sufficient in our phylogenetic tree, it is likely that the constituent species of the *B. tabaci* complex have a separate origin from other *Bemisia* species. Future studies would therefore benefit from a detailed examination as to whether or not the *B. tabaci* complex is paraphyletic to other *Bemisia* species.

The phylogenetic tree and the CDA plots based on the 212 *COI* sequences of *B. tabaci* produced 31 groups, matching the 31 “species” discussed in previous molecular studies. Among these 31 species, 30 have often been reported in several molecular stuides conducted since that of Dinsdale et al. [9]. However, the present study is the first to incorporate *JpL* sequences (AB308111, AB308114–AB308117, and AB240697.1) into analysis of the *B. tabaci* complex since Ueda et al. [15]. Samples of the *JpL* species were collected on four host plants in Japan: *Lonicera japonica* (Caprifoliaceae), *Perilla frutescens* (Lamiaceae), *Solanum lycopersicum*, and *Solanum melongena* (Solanaceae) [15]. Because the *JpL* species was separated from the other 30 species of the *B. tabaci* complex in the phylogenetic tree (Figure 2), additional studies are required to determine whether the *JpL* species indeed belongs to the *B. tabaci* complex or constitute a distinct *Bemisia* species.

### New Threshold for Species Boundary

Dinsdale et al. [9] suggested the 3.5% boundary for distinguishing 24 species of the *B. tabaci* complex. However, in this study, we observed that estimates of intraspecific genetic divergence were higher than 3.5% for six species (*Asia II 1*, *Asia II 7*, *Australia*, *Mediterranean*, *New world*, and *Sub Saharan Africa 1*) (Figure 4). We also calculated 22366 pairwise genetic distances for the 212 *COI* sequences, and found a gap at 4% in the distribution of genetic divergences (Figure 5). This implies that a genetic divergence of 4% could be a threshold that distinguishes divergences within the same species from divergences between species. Thus, the threshold of the species boundary in the *B. tabaci* complex is required to be replaced with 4.0%.

The difference between 3.5% boundary and 4.0% boundary (in this study) may be due to the addition of *COI* sequences that were reported after or not included in Dinsdale et al. [9]. For example, for *Asia II 7*, intraspecific genetic divergences ranged from 0.3% to 2.9% using five *COI* sequences [9]. However, after adding six more *COI* sequences, the largest genetic divergence increased up to 3.8%. The results of aphid genetic divergences implied that if more sequence data were used and if the pattern of genetic divergences in the *B. tabaci* complex is similar to aphids, the species boundary in the *B. tabaci* complex could increase from 4.0% to 6.0%. This study suggests that the current genetic boundary could vary depending on the sample number of *COI* sequences.

### Additional Molecular Makers in the *B. tabaci* Complex

Comparisons of genetic divergences among the 11 mitochondrial genes indicate that respective genes have different patterns of genetic divergences (Table S3; Figure 6). The reason for different levels of genetic divergence among mitochondrial genes has not been clearly explained; however, we confirmed that the *COI* gene exhibits low levels of differentiation compared with the eight genes (*ATP6*, *COII*, *CytB*, *ND1*, *ND2*, *ND3*, *ND4*, and *ND5*) probably due to a low evolutionary rate. Genetic differentiation in the *COI* gene may readily reflect reproductive isolation that occurred a few million years ago [24] without saturation of nucleotide substitutions because of a low evolutionary rate.

Recently, DNA barcode studies have frequently reported that when only *COI* sequences were used, they did not show proper phylogenetic relationships [14], [25], [26]. This indicates that the use of additional molecular markers is required to resolve phylogenetic relationships in the *B. tabaci*. Incorporation of these additional molecular markers will clarify not only the phylogenetic relationships among the species comprising the *B. tabaci* complex but also the taxonomic status of this complex.

## Conclusion

In this study, we confirm that the *B. tabaci* complex is composed of 31 distinct groups or “species” and that the genetic divergence between some species corresponded to that found between different genera or subfamilies. In addition, we observe that the 4.0% genetic boundary was more realistic than the 3.5% in distinguishing species in the *B. tabaci* complex. However, it is not confirmed whether the *JpL* species belongs to the *B. tabaci* complex or not.

Until now, in the *B. tabaci* complex, morphological variation has not been emphasized in spite of large genetic variation among several species [27]. However, in the phylogentic tree, *B. atriplex* that was distinguished from *B. tabaci* based on morphological characters [28] was clustered with some lineages of *B. tabaci*. Thus, it is necessary to detect morphological differences among several species groups of *B. tabaci*. Although the present study provides useful genetic information in understanding the *B. tabaci* complex, morphology and reproductive incompatibility tests must be essential for applying the biological species concept to the *B. tabaci* complex.

## Materials and Methods

### Selecting *COI* Sequences of *Bemisia tabaci* and Hemipteran Species

We obtained 15465 *COI* sequences in the Hemiptera from the GenBank (http://www.ncbi.nlm.nih.gov/genbank/) by using two keywords, ´Hemipterá and ´*COÍ*. To select 650 bp *COI* sequences located in the 3′ region from the 15465 *COI* sequences, alignments were conducted using both CLUSTALW 1.83 [29], with default parameters, and a BLAST search [30] in the Insect Mitochondrial Genome Database (IMGD; http://www.imgd.or/) [31]. A complete *COI* sequence of *B. tabaci* (NC 006279) was used as the standard sequence in the alignments.

A total of 1568 *COI* sequences belonging to 154 species were selected based on the alignment results. The 1568 *COI* sequences consisted of 1059 *COI* sequences of *B. tabaci* and 509 *COI* sequences of 153 hemipteran species belonging to 53 genera and 8 families (Table S4).

### Comparing Genetic Divergence between *B. tabaci* and Other Hemipteran Species

Firstly, three datasets were constructed from the 1568 *COI* sequences of *B. tabaci* and the 153 hemipteran species: i) *B. tabaci* dataset consisting of 1059 *COI* sequences of *B. tabaci*, ii) hemipteran species dataset consisting of 509 *COI* sequences of the 153 hemipteran species, and iii) combined dataset consisting of 1568 *COI* sequences of the 153 hemipteran species+*B. tabaci*. The alignments of *COI* sequences of the three datasets were conducted by using both CLUSTALW 1.83 [29], with default parameters, and a BLAST search [30] in the IMGD [31].

Genetic divergence values for the three datasets were calculated using the ´distance matrix analysis functioń implemented in the IMGD. Genetic divergence of the *B. tabaci* dataset was calculated at one taxonomic level, among conspecific individuals, whereas those of the remaining datasets were analyzed according to three taxonomic levels, 1) among conspecific individuals, 2) among congeneric species, and 3) among confamilial species.

### Defining the Number of Species of the *B. tabaci* Complex

To construct a phylogenetic tree, we first conducted neighbor-joining (NJ) analyses based on the 1059 *COI* sequences of *B. tabaci* using MEGA 5.0 [32] and excluded duplicate haplotypes in these sequences based on the NJ tree. The duplicate haplotypes were rechecked using TCS [33]. In total, 212 *COI* sequences (20% out of the 1059 *COI* sequences) were selected.

Phylogenetic trees were constructed based on the 212 *COI* sequences of *B. tabaci* and ten *COI* sequences of *Bemisia atriplex*, *Bemisia subdecipiens*, *Bemisia afer*, *Aleurocanthus camelliae*, *Aleurodicus dispersus*, *Aleurocanthus spiniferus*, *Trialeurodes lauri*, *Trialeurodes ricini*, and *Trialeurodes vaporariorum* as one outgroup. Alignments of nucleotide sequences were performed using CLUSTALX with default conditions [29]. The well-aligned blocks from the nucleotide sequences were then selected with GBlocks 0.91b, again under default conditions [34]. Phylogenetic reconstruction was done using Bayesian inference (BI) analysis. The BI trees were obtained using MrBayes 3.1.2 [35] and the Markov Chain Monte Carlo technique (MCMC). We chose the GTR+G model for the dataset based on the hierarchical likelihood ratio test that was conducted using MrModeltest v2.2 [36]. Model parameter values were treated as unknowns and were estimated for each analysis. Random starting trees were used, and analyses were run for 10000000 generations with sampling done every 100 generations for the *B. tabaci* dataset. Bayesian posterior probabilities were then calculated from the sample points after the MCMC algorithm began to converge. To ensure that our analyses are not trapped in local optima, four independent MCMC runs were performed. Topologies and posterior clade probabilities from different runs were compared for congruence.

Because of the large number of *COI* sequences of *B. tabaci*, a canonical discriminant analysis (CDA), as implemented in SPSS (IBM® SPSS Statistics, Ver 20, IBM), was used to demonstrate a graphical summary of the species-grouping results. CDA explores differences in species assemblage compositions by using non-parametric permutation tests which provide exact probability values, and the similarity or dissimilarity of the various sampling units are graphically displayed. A matrix was constructed based on 22336 pairwise genetic distances from the 212 *COI* sequences using on the Kimura 2-parameter (K2P) distance model [37] in MEGA 5.0 [32].

### Comparing Intraspecific and Interspecific Genetic Divergences

The 212 *COI* sequences were categorized into several species based on the phylogenetic tree (see Results). We tentatively named a total of 31 species based on five previous studies: *Asia I*, *Asia II 1*, *Asia II 2*, *Asia II 3*, *Asia II 4*, *Asia II 5*, *Asia II 6*, *Asia II 7*, *Asia II 8*, *Australia*, *Australia/Indonesia*, *China 1*, *China 2*, *Italy*, *Mediterranean*, *Middle East Asia Minor 1*, *Middle East Asia Minor 2*, *Indian Ocean*, *New World*, *Sub Saharan Africa 1*, *Sub Saharan Africa 2*, *Sub Saharan Africa 3*, *Sub Saharan Africa 4*, and *Uganda* [9], *JpL* [15], *Asia I-India* [11], *Asia III*, *China 3*, *Asia II 9*, *Asia II 10* [13], and *New World 2* [12]. Of these species, seven species, *Asia I-India*, *Asia II 2*, *Asia II 4*, *China 3*, *Middle East Asia Minor 2*, *Sub Saharan Africa 3*, and *Uganda*, were excluded in this analysis because each group included only a single haplotype. Intraspecific genetic divergences were analyzed based on the remaining 24 species.

Interspecific genetic divergence was calculated between pairs of closely related species. We selected nine pairs of closely related species consisting of ten species based on the phylogenetic tree (see Results): *Asia II 1−Asia II 2, Asia I−Asia III*, *Asia II 3−Asia II 4*, *Asia II 5–Asia I-India*, *Australia/Indonesia−Australia*, *China 1−China 2*, *Middle East Asia Minor 1−Middle East Asia Minor 2*, *New World−New World 2*, and *Sub Saharan Africa 2−Sub Saharan Africa 4*.

All intraspecific and interspecific genetic divergence estimates were calculated using the *p*-distance model and the K2P distance model [37] of MEGA 5.0 [32], respectively.

### Analyzing Genetic Divergences of Aphid Species

A total of 47 *COI* sequences of 34 aphid species were downloaded from the Genbank (Table S5). To analyze sequences of the same length and position as in the *B. tabaci* dataset, the 47 *COI* sequences were aligned with *B. tabaci COI* sequences by using CLUSTALW 1.83 [29]. Genetic divergence of the aphid species was calculated according to three taxonomic levels, 1) among conspecific individuals, 2) among congeneric species, and 3) among species of different genera in the same family, by using the K2P distance model [37] of MEGA 5.0 [32].

### Comparing Genetic Divergences of Eleven Mitochondrial Genes

To compare the pattern of genetic divergences among genes, we chose eleven mitochondrial genes, ATP synthase F0 subunits 6 (ATP6), COI, cytochrome oxidase subunits II (COII), cytochrome oxidase subunits III (COIII), NADH dehydrogenase subunits 1–5 (ND1, ND2, ND3, ND4, and ND5), large subunit ribosomal RNA (lrRNA), and small subunit ribosomal RNA (srRNA), which have frequently been used in insect molecular studies. A total of 20 complete mitochondrial genomes belonging to the order Hemiptera were downloaded from the Genbank (Table S6). The genetic divergences of the eleven genes were calculated according to two taxonomic levels (between genera within the same suborder and between suborder within the same order) by using the K2P distance model [37] of MEGA 5.0 [32]. Wilcoxon signed rank tests were performed to compare interspecific variability between COI and other ten genes following Kress & Erickson [38].

## Supporting Information

Table S1 **Intraspecific generic divergences about 31 species of the** ***Bemisia tabaci*** **complex.**
(DOC)

Table S2 **Intraspecific generic divergences between nine species pairs.**
(DOC)

Table S3 **Genetic divergences of 11 mitochondrial genes in 20 hemipteran complete mitochondrial genomes.**
(DOC)

Table S4 **The list of 1059 individuals of** ***Bemisia tabaci*** **and 509 individuals of 153 hemipteran species, 53 genera, and 8 subfamilies.**
(DOC)

Table S5 **The list of 47 individuals of 34 aphid species.**
(DOC)

Table S6 **The list of 20 hemipterea mitochondrial genomes.**
(DOC)
