# Supplementary material for: Taxonomic Status of the Bemisia tabaci Complex (Hemiptera: Aleyrodidae) and Reassessment of the Number of Its Constituent Species
Source: PLoS One. 2013 May 13;8(5):e63817. doi: 10.1371/journal.pone.0063817 (PMC3652838; doi:10.1371/journal.pone.0063817)
Supplement: Table S1 — Intraspecific generic divergences about 31 species of the Bemisia tabaci complex. (DOC) [file pone.0063817.s001.doc]

**Table S1** Intraspecific generic divergences about 31 species of the *Bemisia tabaci* complex

|  | **Number of sequences** | ***p*-distance** | | | **K2P distance** | | | **N.C.^d^** |
| --- | --- | --- | --- | --- | --- | --- | --- | --- |
|  |  | **Avg.^a^(%)** | **Min.^b^(%)** | **Max.^c^(%)** | **Avg.(%)** | **Min.(%)** | **Max(%)** |  |
| *Asia I* | 24 | 0.8 | 0.2 | 3.3 | 0.8 | 0.2 | 3.4 | 276 |
| *Asia II 1* | 7 | 1.9 | 0.3 | 3.7 | 2.0 | 0.3 | 3.8 | 21 |
| *Asia II 2* | 1 |  |  |  |  |  |  |  |
| *Asia II 3* | 2 | 0.8 |  |  | 0.8 |  |  | 1 |
| *Asia II 4* | 1 |  |  |  |  |  |  |  |
| *Asia II 5* | 6 | 1.0 | 0.2 | 2.4 | 1.0 | 0.2 | 2.5 | 15 |
| *Asia II 6* | 2 | 3.1 |  |  | 3.2 |  |  | 1 |
| *Asia II 7* | 10 | 1.7 | 0.2 | 3.8 | 1.7 | 0.3 | 3.9 | 45 |
| *Asia II 8* | 7 | 0.6 | 0.2 | 1.1 | 0.6 | 0.2 | 1.1 | 21 |
| *Asia II 9* | 2 | 0.5 |  |  | 0.5 |  |  | 1 |
| *Asia II 10* | 2 | 0.5 |  |  | 0.5 |  |  | 1 |
| *Asia III* | 2 | 0.5 |  |  | 0.5 |  |  | 1 |
| *Australia* | 2 | 3.7 |  |  | 3.8 |  |  | 1 |
| *Australia/Indonesia* | 4 | 0.8 | 0.5 | 1.1 | 0.8 | 0.5 | 1.1 | 6 |
| *China 1* | 3 | 0.9 | 0.5 | 1.2 | 0.9 | 0.5 | 1.2 | 3 |
| *China 2* | 2 | 1.5 |  |  | 1.5 |  |  | 1 |
| *China 3* | 1 |  |  |  |  |  |  |  |
| *Indian Ocean* | 12 | 0.9 | 0.2 | 2.1 | 0.9 | 0.2 | 2.1 | 66 |
| *Italy* | 3 | 0.5 | 0.4 | 0.6 | 0.5 | 0.4 | 0.7 | 3 |
| *JpL* | 5 | 0.4 | 0.2 | 0.7 | 0.4 | 0.2 | 0.7 | 10 |
| *Mediterranean* | 45 | 1.4 | 0.2 | 3.9 | 1.4 | 0.2 | 4.0 | 990 |
| *Middle East Asia Minor 1* | 30 | 0.7 | 0.2 | 1.5 | 0.7 | 0.2 | 1.5 | 435 |
| *Middle East Asia Minor 2* | 1 |  |  |  |  |  |  |  |
| *New World* | 13 | 1.4 | 0.2 | 3.5 | 1.4 | 0.2 | 3.6 | 78 |
| *New World 2* | 2 | 0.5 |  |  | 0.5 |  |  | 1 |
| *Sub Saharan Africa 1* | 18 | 1.6 | 0.3 | 3.5 | 1.6 | 0.3 | 3.6 | 153 |
| *Sub Saharan Africa 2* | 9 | 0.9 | 0.2 | 2.3 | 0.9 | 0.2 | 2.3 | 36 |
| *Sub Saharan Africa 3* | 1 |  |  |  |  |  |  |  |
| *Sub Saharan Africa4* | 5 | 1.0 | 0.3 | 1.8 | 1.0 | 0.3 | 1.9 | 10 |
| *Uganda* | 1 |  |  |  |  |  |  |  |

^a^Average, ^b^Minimum, ^c^Maximum, and ^d^Number of comparisons.
