# Supplementary material for: Taxonomic Status of the Bemisia tabaci Complex (Hemiptera: Aleyrodidae) and Reassessment of the Number of Its Constituent Species
Source: PLoS One. 2013 May 13;8(5):e63817. doi: 10.1371/journal.pone.0063817 (PMC3652838; doi:10.1371/journal.pone.0063817)
Supplement: Table S2 — Intraspecific generic divergences between nine species pairs. (DOC) [file pone.0063817.s002.doc]

**Table S2 Intraspecific generic divergences between nine species pairs.**

|  | ***p*-distance** | | | **K2P distance** | | | **N.C.^d^** |
| --- | --- | --- | --- | --- | --- | --- | --- |
|  | **Avg.^a^(%)** | **Min.^b^(%)** | **Max.^c^(%)** | **Avg.(%)** | **Min.(%)** | **Max(%)** |  |
| *China 1 − China 2* | 4.4 | 4.0 | 5.0 | 4.5 | 4.2 | 5.2 | 6 |
| *Asia II 5 − Aisa I-India* | 8.6 | 8.1 | 9.9 | 9.3 | 8.7 | 10.8 | 6 |
| *Middle East Asia Minor 1 − Middle East Asia Minor 2* | 4.8 | 4.2 | 8.3 | 5.0 | 4.4 | 8.9 | 33 |
| *Asia II 3 − Asia II 4* | 4.9 | 4.7 | 5.2 | 5.1 | 4.8 | 5.4 | 2 |
| *New World − New World 2* | 5.5 | 4.7 | 6.4 | 5.8 | 4.90 | 6.8 | 26 |
| *Asia I − Asia III* | 7.7 | 6.9 | 9.9 | 8.2 | 7.3 | 10.8 | 72 |
| *Asia II 1 − Asia II 2* | 6.9 | 5.1 | 8.2 | 7.3 | 5.3 | 8.8 | 7 |
| *Sub Saharan Africa 2 − Sub Saharan Africa 4* | 7.4 | 7.0 | 8.1 | 7.9 | 7.4 | 8.6 | 45 |
| *Australia − Australiea/Indonesia* | 14.0 | 12.5 | 15.5 | 16.0 | 14.1 | 18.0 | 8 |

^a^Average, ^b^Minimum, ^c^Maximum, and ^d^Number of comparisons.
