# Supplementary material for: Taxonomic Status of the Bemisia tabaci Complex (Hemiptera: Aleyrodidae) and Reassessment of the Number of Its Constituent Species
Source: PLoS One. 2013 May 13;8(5):e63817. doi: 10.1371/journal.pone.0063817 (PMC3652838; doi:10.1371/journal.pone.0063817)
Supplement: Table S3 — Genetic divergences of 11 mitochondrial genes in 20 hemipteran complete mitochondrial genomes. (DOC) [file pone.0063817.s003.doc]

**Table S3** Genetic divergences of 11 mitochondrial genes in 20 hemipteran complete mitochondrial genomes.

|  | **Between genera of suborder** | | | **Bewteeen suborder** | | |
| --- | --- | --- | --- | --- | --- | --- |
|  | **Avg.^a^(%)** | **Min.^b^(%)** | **Max.^c^(%)** | **Avg.^a^(%)** | **Min.^b^(%)** | **Max.^c^(%)** |
| *COI* | 28.0 | 16.3 | 51.4 | 40.2 | 24.2 | 56.9 |
| *COII* | 35.5 | 19.0 | 60.8 | 49.8 | 29.7 | 71.3 |
| *CytB* | 32.6 | 21.2 | 55.2 | 47.4 | 28.7 | 62.3 |
| *ND1* | 36.1 | 19.0 | 84.9 | 58.3 | 32.8 | 84.1 |
| *ND2* | 48.7 | 22.1 | 93.3 | 65.0 | 28.3 | 95.7 |
| *ND3* | 46.5 | 23.2 | 88.8 | 64.8 | 40.7 | 93.4 |
| *ND4* | 45.2 | 21.3 | 82.5 | 66.3 | 36.0 | 95.2 |
| *ND5* | 42.4 | 22.5 | 80.2 | 65.2 | 35.7 | 94.8 |
| *ATP6* | 41.2 | 24.0 | 71.1 | 57.3 | 31.8 | 81.6 |
| *lrRNA* | 21.1 | 13.0 | 38.0 | 34.5 | 18.8 | 45.8 |
| *srRNA* | 21.3 | 10.0 | 41.8 | 33.9 | 15.9 | 53.5 |

^a^Average, ^b^Minimum, and ^c^Maximum.
