# Supplementary material for: Taxonomic Status of the Bemisia tabaci Complex (Hemiptera: Aleyrodidae) and Reassessment of the Number of Its Constituent Species
Source: PLoS One. 2013 May 13;8(5):e63817. doi: 10.1371/journal.pone.0063817 (PMC3652838; doi:10.1371/journal.pone.0063817)
Supplement: Table S4 — The list of 1059 individuals of Bemisia tabaci and 509 individuals of 153 hemipteran species, 53 genera, and 8 subfamilies. (DOC) [file pone.0063817.s004.doc]

**Table S4** The list of 1059 individuals of *Bemisia tabaci* and 509 individuals of 153 hemipteran species, 53 genera, and 8 subfamilies.

| **Suborder** | **Family** | **Species** | **Accession number** |
| --- | --- | --- | --- |
| Sternorrhyncha | Aleyrodidae | *Bemisia tabaci* | AM691059 |
| Sternorrhyncha | Aleyrodidae | *Bemisia tabaci* | AM691064 |
| Sternorrhyncha | Aleyrodidae | *Bemisia tabaci* | AM691062 |
| Sternorrhyncha | Aleyrodidae | *Bemisia tabaci* | AM176575 |
| Sternorrhyncha | Aleyrodidae | *Bemisia tabaci* | AY827579 |
| Sternorrhyncha | Aleyrodidae | *Bemisia tabaci* | AY827580 |
| Sternorrhyncha | Aleyrodidae | *Bemisia tabaci* | AY827588 |
| Sternorrhyncha | Aleyrodidae | *Bemisia tabaci* | AY827590 |
| Sternorrhyncha | Aleyrodidae | *Bemisia tabaci* | AY827582 |
| Sternorrhyncha | Aleyrodidae | *Bemisia tabaci* | AY827587 |
| Sternorrhyncha | Aleyrodidae | *Bemisia tabaci* | AY827589 |
| Sternorrhyncha | Aleyrodidae | *Bemisia tabaci* | AM691055 |
| Sternorrhyncha | Aleyrodidae | *Bemisia tabaci* | AM691063 |
| Sternorrhyncha | Aleyrodidae | *Bemisia tabaci* | AY827612 |
| Sternorrhyncha | Aleyrodidae | *Bemisia tabaci* | AY827614 |
| Sternorrhyncha | Aleyrodidae | *Bemisia tabaci* | AY903578 |
| Sternorrhyncha | Aleyrodidae | *Bemisia tabaci* | DQ133378 |
| Sternorrhyncha | Aleyrodidae | *Bemisia tabaci* | AY827617 |
| Sternorrhyncha | Aleyrodidae | *Bemisia tabaci* | AJ510081 |
| Sternorrhyncha | Aleyrodidae | *Bemisia tabaci* | AJ510076 |
| Sternorrhyncha | Aleyrodidae | *Bemisia tabaci* | AY057181 |
| Sternorrhyncha | Aleyrodidae | *Bemisia tabaci* | AF344267 |
| Sternorrhyncha | Aleyrodidae | *Bemisia tabaci* | AY827591 |
| Sternorrhyncha | Aleyrodidae | *Bemisia tabaci* | AY057182 |
| Sternorrhyncha | Aleyrodidae | *Bemisia tabaci* | AY057168 |
| Sternorrhyncha | Aleyrodidae | *Bemisia tabaci* | AY057146 |
| Sternorrhyncha | Aleyrodidae | *Bemisia tabaci* | AY057173 |
| Sternorrhyncha | Aleyrodidae | *Bemisia tabaci* | AY827605 |
| Sternorrhyncha | Aleyrodidae | *Bemisia tabaci* | AF344257 |
| Sternorrhyncha | Aleyrodidae | *Bemisia tabaci* | AF344252 |
| Sternorrhyncha | Aleyrodidae | *Bemisia tabaci* | AJ748361 |
| Sternorrhyncha | Aleyrodidae | *Bemisia tabaci* | AJ748365 |
| Sternorrhyncha | Aleyrodidae | *Bemisia tabaci* | AJ748370 |
| Sternorrhyncha | Aleyrodidae | *Bemisia tabaci* | AJ748376 |
| Sternorrhyncha | Aleyrodidae | *Bemisia tabaci* | AJ784261 |
| Sternorrhyncha | Aleyrodidae | *Bemisia tabaci* | AY057151 |
| Sternorrhyncha | Aleyrodidae | *Bemisia tabaci* | [AY827604.1](http://www.ncbi.nlm.nih.gov/nuccore/57790828) |
| Sternorrhyncha | Aleyrodidae | *Bemisia tabaci* | [DQ116641.1](http://www.ncbi.nlm.nih.gov/nuccore/76577918) |
| Sternorrhyncha | Aleyrodidae | *Bemisia tabaci* | [AY563685.1](http://www.ncbi.nlm.nih.gov/nuccore/58003858) |
| Sternorrhyncha | Aleyrodidae | *Bemisia tabaci* | DQ989548.1 |
| Sternorrhyncha | Aleyrodidae | *Bemisia tabaci* | AY563656.1 |
| Sternorrhyncha | Aleyrodidae | *Bemisia tabaci* | [EF566756.1](http://www.ncbi.nlm.nih.gov/nuccore/148575377) |
| Sternorrhyncha | Aleyrodidae | *Bemisia tabaci* | AY827596.1 |
| Sternorrhyncha | Aleyrodidae | *Bemisia tabaci* | DQ116664.1 |
| Sternorrhyncha | Aleyrodidae | *Bemisia tabaci* | AJ748374.1 |
| Sternorrhyncha | Aleyrodidae | *Bemisia tabaci* | AY057139.1 |
| Sternorrhyncha | Aleyrodidae | *Bemisia tabaci* | AY057129 |
| Sternorrhyncha | Aleyrodidae | *Bemisia tabaci* | DQ309075.1 |
| Sternorrhyncha | Aleyrodidae | *Bemisia tabaci* | AY686095 |
| Sternorrhyncha | Aleyrodidae | *Bemisia tabaci* | DQ989535.1 |
| Sternorrhyncha | Aleyrodidae | *Bemisia tabaci* | EU376977.1 |
| Sternorrhyncha | Aleyrodidae | *Bemisia tabaci* | AY827609.1 |
| Sternorrhyncha | Aleyrodidae | *Bemisia tabaci* | EF398107.1 |
| Sternorrhyncha | Aleyrodidae | *Bemisia tabaci* | DQ174527.1 |
| Sternorrhyncha | Aleyrodidae | *Bemisia tabaci* | DQ989544.1 |
| Sternorrhyncha | Aleyrodidae | *Bemisia tabaci* | EU376978.1 |
| Sternorrhyncha | Aleyrodidae | *Bemisia tabaci* | EF398112.1 |
| Sternorrhyncha | Aleyrodidae | *Bemisia tabaci* | AY686062.1 |
| Sternorrhyncha | Aleyrodidae | *Bemisia tabaci* | EF398095.1 |
| Sternorrhyncha | Aleyrodidae | *Bemisia tabaci* | AY057123.1 |
| Sternorrhyncha | Aleyrodidae | *Bemisia tabaci* | DQ365878.1 |
| Sternorrhyncha | Aleyrodidae | *Bemisia tabaci* | AY827613 |
| Sternorrhyncha | Aleyrodidae | *Bemisia tabaci* | AY686086.1 |
| Sternorrhyncha | Aleyrodidae | *Bemisia tabaci* | EF398090.1 |
| Sternorrhyncha | Aleyrodidae | *Bemisia tabaci* | AY563666.1 |
| Sternorrhyncha | Aleyrodidae | *Bemisia tabaci* | AJ510058.1 |
| Sternorrhyncha | Aleyrodidae | *Bemisia tabaci* | AY057144.1 |
| Sternorrhyncha | Aleyrodidae | *Bemisia tabaci* | AJ510065 |
| Sternorrhyncha | Aleyrodidae | *Bemisia tabaci* | AJ748358.1 |
| Sternorrhyncha | Aleyrodidae | *Bemisia tabaci* | DQ989528.1 |
| Sternorrhyncha | Aleyrodidae | *Bemisia tabaci* | AY827587.1 |
| Sternorrhyncha | Aleyrodidae | *Bemisia tabaci* | AY057184.1 |
| Sternorrhyncha | Aleyrodidae | *Bemisia tabaci* | EU000320.1 |
| Sternorrhyncha | Aleyrodidae | *Bemisia tabaci* | AB204583.1 |
| Sternorrhyncha | Aleyrodidae | *Bemisia tabaci* | AM944347.1 |
| Sternorrhyncha | Aleyrodidae | *Bemisia tabaci* | EU427724.1 |
| Sternorrhyncha | Aleyrodidae | *Bemisia tabaci* | AM040592.1 |
| Sternorrhyncha | Aleyrodidae | *Bemisia tabaci* | EU255283.1 |
| Sternorrhyncha | Aleyrodidae | *Bemisia tabaci* | AJ748378.1 |
| Sternorrhyncha | Aleyrodidae | *Bemisia tabaci* | EF694106.1 |
| Sternorrhyncha | Aleyrodidae | *Bemisia tabaci* | AF164671 |
| Sternorrhyncha | Aleyrodidae | *Bemisia tabaci* | [DQ989553.1](http://www.ncbi.nlm.nih.gov/nuccore/119352546) |
| Sternorrhyncha | Aleyrodidae | *Bemisia tabaci* | AY563670.1 |
| Sternorrhyncha | Aleyrodidae | *Bemisia tabaci* | DQ365875.1 |
| Sternorrhyncha | Aleyrodidae | *Bemisia tabaci* | EU000317.1 |
| Sternorrhyncha | Aleyrodidae | *Bemisia tabaci* | AJ550179.1 |
| Sternorrhyncha | Aleyrodidae | *Bemisia tabaci* | AY686064 |
| Sternorrhyncha | Aleyrodidae | *Bemisia tabaci* | AM176573 |
| Sternorrhyncha | Aleyrodidae | *Bemisia tabaci* | AY057163.1 |
| Sternorrhyncha | Aleyrodidae | *Bemisia tabaci* | AJ557145.1 |
| Sternorrhyncha | Aleyrodidae | *Bemisia tabaci* | EF694109.1 |
| Sternorrhyncha | Aleyrodidae | *Bemisia tabaci* | AY563662.1 |
| Sternorrhyncha | Aleyrodidae | *Bemisia tabaci* | AM040603.1 |
| Sternorrhyncha | Aleyrodidae | *Bemisia tabaci* | AY686077.1 |
| Sternorrhyncha | Aleyrodidae | *Bemisia tabaci* | EU000312.1 |
| Sternorrhyncha | Aleyrodidae | *Bemisia tabaci* | EF398120.1 |
| Sternorrhyncha | Aleyrodidae | *Bemisia tabaci* | AM040608.1 |
| Sternorrhyncha | Aleyrodidae | *Bemisia tabaci* | AJ748379.1 |
| Sternorrhyncha | Aleyrodidae | *Bemisia tabaci* | DQ174533.1 |
| Sternorrhyncha | Aleyrodidae | *Bemisia tabaci* | AY827616.1 |
| Sternorrhyncha | Aleyrodidae | *Bemisia tabaci* | AB204578 |
| Sternorrhyncha | Aleyrodidae | *Bemisia tabaci* | AY057136.1 |
| Sternorrhyncha | Aleyrodidae | *Bemisia tabaci* | AY686066.1 |
| Sternorrhyncha | Aleyrodidae | *Bemisia tabaci* | EF566757.1 |
| Sternorrhyncha | Aleyrodidae | *Bemisia tabaci* | AB204585.1 |
| Sternorrhyncha | Aleyrodidae | *Bemisia tabaci* | EF667474.1 |
| Sternorrhyncha | Aleyrodidae | *Bemisia tabaci* | DQ989552.1 |
| Sternorrhyncha | Aleyrodidae | *Bemisia tabaci* | AY563692.1 |
| Sternorrhyncha | Aleyrodidae | *Bemisia tabaci* | AY057180.1 |
| Sternorrhyncha | Aleyrodidae | *Bemisia tabaci* | AJ550174 |
| Sternorrhyncha | Aleyrodidae | *Bemisia tabaci* | DQ174522.1 |
| Sternorrhyncha | Aleyrodidae | *Bemisia tabaci* | AM408901.1 |
| Sternorrhyncha | Aleyrodidae | *Bemisia tabaci* | DQ116660.1 |
| Sternorrhyncha | Aleyrodidae | *Bemisia tabaci* | EF398089.1 |
| Sternorrhyncha | Aleyrodidae | *Bemisia tabaci* | DQ989529.1 |
| Sternorrhyncha | Aleyrodidae | *Bemisia tabaci* | DQ130058.1 |
| Sternorrhyncha | Aleyrodidae | *Bemisia tabaci* | DQ365874.1 |
| Sternorrhyncha | Aleyrodidae | *Bemisia tabaci* | DQ989522.1 |
| Sternorrhyncha | Aleyrodidae | *Bemisia tabaci* | EF398108.1 |
| Sternorrhyncha | Aleyrodidae | *Bemisia tabaci* | AY057171.1 |
| Sternorrhyncha | Aleyrodidae | *Bemisia tabaci* | AM408897.1 |
| Sternorrhyncha | Aleyrodidae | *Bemisia tabaci* | DQ365866.1 |
| Sternorrhyncha | Aleyrodidae | *Bemisia tabaci* | DQ989527.1 |
| Sternorrhyncha | Aleyrodidae | *Bemisia tabaci* | EF566760.1 |
| Sternorrhyncha | Aleyrodidae | *Bemisia tabaci* | EF398099.1 |
| Sternorrhyncha | Aleyrodidae | *Bemisia tabaci* | AY057189.1 |
| Sternorrhyncha | Aleyrodidae | *Bemisia tabaci* | DQ174518.1 |
| Sternorrhyncha | Aleyrodidae | *Bemisia tabaci* | AM944348.1 |
| Sternorrhyncha | Aleyrodidae | *Bemisia tabaci* | AF418664.2 |
| Sternorrhyncha | Aleyrodidae | *Bemisia tabaci* | EF398126.1 |
| Sternorrhyncha | Aleyrodidae | *Bemisia tabaci* | DQ130054.1 |
| Sternorrhyncha | Aleyrodidae | *Bemisia tabaci* | DQ174523.1 |
| Sternorrhyncha | Aleyrodidae | *Bemisia tabaci* | DQ989540.1 |
| Sternorrhyncha | Aleyrodidae | *Bemisia tabaci* | AY686075 |
| Sternorrhyncha | Aleyrodidae | *Bemisia tabaci* | AY563649.1 |
| Sternorrhyncha | Aleyrodidae | *Bemisia tabaci* | AY563658.1 |
| Sternorrhyncha | Aleyrodidae | *Bemisia tabaci* | DQ130063.1 |
| Sternorrhyncha | Aleyrodidae | *Bemisia tabaci* | AJ510078.1 |
| Sternorrhyncha | Aleyrodidae | *Bemisia tabaci* | AF340215.1 |
| Sternorrhyncha | Aleyrodidae | *Bemisia tabaci* | AJ748359 |
| Sternorrhyncha | Aleyrodidae | *Bemisia tabaci* | AF342775 |
| Sternorrhyncha | Aleyrodidae | *Bemisia tabaci* | AY563699.1 |
| Sternorrhyncha | Aleyrodidae | *Bemisia tabaci* | AY611642.1 |
| Sternorrhyncha | Aleyrodidae | *Bemisia tabaci* | DQ989534.1 |
| Sternorrhyncha | Aleyrodidae | *Bemisia tabaci* | DQ365858.1 |
| Sternorrhyncha | Aleyrodidae | *Bemisia tabaci* | AJ748371 |
| Sternorrhyncha | Aleyrodidae | *Bemisia tabaci* | EU427719.1 |
| Sternorrhyncha | Aleyrodidae | *Bemisia tabaci* | AJ550169.1 |
| Sternorrhyncha | Aleyrodidae | *Bemisia tabaci* | AJ748369 |
| Sternorrhyncha | Aleyrodidae | *Bemisia tabaci* | AY686074.1 |
| Sternorrhyncha | Aleyrodidae | *Bemisia tabaci* | EF398088.1 |
| Sternorrhyncha | Aleyrodidae | *Bemisia tabaci* | AM943517.1 |
| Sternorrhyncha | Aleyrodidae | *Bemisia tabaci* | [AJ510062.1](http://www.ncbi.nlm.nih.gov/nuccore/46016180) |
| Sternorrhyncha | Aleyrodidae | *Bemisia tabaci* | EF398103.1 |
| Sternorrhyncha | Aleyrodidae | *Bemisia tabaci* | AY057183.1 |
| Sternorrhyncha | Aleyrodidae | *Bemisia tabaci* | AF344254 |
| Sternorrhyncha | Aleyrodidae | *Bemisia tabaci* | AJ748381.1 |
| Sternorrhyncha | Aleyrodidae | *Bemisia tabaci* | EU376982.1 |
| Sternorrhyncha | Aleyrodidae | *Bemisia tabaci* | DQ130059.1 |
| Sternorrhyncha | Aleyrodidae | *Bemisia tabaci* | DQ133381.1 |
| Sternorrhyncha | Aleyrodidae | *Bemisia tabaci* | AY057210.1 |
| Sternorrhyncha | Aleyrodidae | *Bemisia tabaci* | DQ174536.1 |
| Sternorrhyncha | Aleyrodidae | *Bemisia tabaci* | DQ989550.1 |
| Sternorrhyncha | Aleyrodidae | *Bemisia tabaci* | EU376987.1 |
| Sternorrhyncha | Aleyrodidae | *Bemisia tabaci* | DQ130055.1 |
| Sternorrhyncha | Aleyrodidae | *Bemisia tabaci* | AY057169 |
| Sternorrhyncha | Aleyrodidae | *Bemisia tabaci* | AF344247.1 |
| Sternorrhyncha | Aleyrodidae | *Bemisia tabaci* | DQ174526.1 |
| Sternorrhyncha | Aleyrodidae | *Bemisia tabaci* | EU255277.1 |
| Sternorrhyncha | Aleyrodidae | *Bemisia tabaci* | AY057204.1 |
| Sternorrhyncha | Aleyrodidae | *Bemisia tabaci* | DQ130061.1 |
| Sternorrhyncha | Aleyrodidae | *Bemisia tabaci* | AJ550180.1 |
| Sternorrhyncha | Aleyrodidae | *Bemisia tabaci* | EU376984.1 |
| Sternorrhyncha | Aleyrodidae | *Bemisia tabaci* | DQ365856 |
| Sternorrhyncha | Aleyrodidae | *Bemisia tabaci* | AY057186.1 |
| Sternorrhyncha | Aleyrodidae | *Bemisia tabaci* | AY057186.1 |
| Sternorrhyncha | Aleyrodidae | *Bemisia tabaci* | AY057175.1 |
| Sternorrhyncha | Aleyrodidae | *Bemisia tabaci* | AJ510080.1 |
| Sternorrhyncha | Aleyrodidae | *Bemisia tabaci* | DQ130056.1 |
| Sternorrhyncha | Aleyrodidae | *Bemisia tabaci* | DQ989532.1 |
| Sternorrhyncha | Aleyrodidae | *Bemisia tabaci* | DQ989524.1 |
| Sternorrhyncha | Aleyrodidae | *Bemisia tabaci* | AM040596.1 |
| Sternorrhyncha | Aleyrodidae | *Bemisia tabaci* | DQ365864.1 |
| Sternorrhyncha | Aleyrodidae | *Bemisia tabaci* | AB248260 |
| Sternorrhyncha | Aleyrodidae | *Bemisia tabaci* | DQ174529.1 |
| Sternorrhyncha | Aleyrodidae | *Bemisia tabaci* | DQ989545.1 |
| Sternorrhyncha | Aleyrodidae | *Bemisia tabaci* | DQ989525.1 |
| Sternorrhyncha | Aleyrodidae | *Bemisia tabaci* | DQ473394.1 |
| Sternorrhyncha | Aleyrodidae | *Bemisia tabaci* | AJ557144.1 |
| Sternorrhyncha | Aleyrodidae | *Bemisia tabaci* | AY686091 |
| Sternorrhyncha | Aleyrodidae | *Bemisia tabaci* | AJ517769 |
| Sternorrhyncha | Aleyrodidae | *Bemisia tabaci* | [AY563641.1](http://www.ncbi.nlm.nih.gov/nuccore/58003790) |
| Sternorrhyncha | Aleyrodidae | *Bemisia tabaci* | AY563696.1 |
| Sternorrhyncha | Aleyrodidae | *Bemisia tabaci* | AB248262.1 |
| Sternorrhyncha | Aleyrodidae | *Bemisia tabaci* | EU376995.1 |
| Sternorrhyncha | Aleyrodidae | *Bemisia tabaci* | EU255286.1 |
| Sternorrhyncha | Aleyrodidae | *Bemisia tabaci* | AJ748362.1 |
| Sternorrhyncha | Aleyrodidae | *Bemisia tabaci* | AJ550171.1 |
| Sternorrhyncha | Aleyrodidae | *Bemisia tabaci* | [DQ116673.1](http://www.ncbi.nlm.nih.gov/nuccore/76577961) |
| Sternorrhyncha | Aleyrodidae | *Bemisia tabaci* | DQ116666.1 |
| Sternorrhyncha | Aleyrodidae | *Bemisia tabaci* | EF694105.1 |
| Sternorrhyncha | Aleyrodidae | *Bemisia tabaci* | AF344284.1 |
| Sternorrhyncha | Aleyrodidae | *Bemisia tabaci* | DQ365861.1 |
| Sternorrhyncha | Aleyrodidae | *Bemisia tabaci* | DQ116646.1 |
| Sternorrhyncha | Aleyrodidae | *Bemisia tabaci* | EF398116.1 |
| Sternorrhyncha | Aleyrodidae | *Bemisia tabaci* | AM040602.1 |
| Sternorrhyncha | Aleyrodidae | *Bemisia tabaci* | EF398102.1 |
| Sternorrhyncha | Aleyrodidae | *Bemisia tabaci* | AY686070.1 |
| Sternorrhyncha | Aleyrodidae | *Bemisia tabaci* | AY686063.1 |
| Sternorrhyncha | Aleyrodidae | *Bemisia tabaci* | AY563704.1 |
| Sternorrhyncha | Aleyrodidae | *Bemisia tabaci* | AY057138 |
| Sternorrhyncha | Aleyrodidae | *Bemisia tabaci* | EF398117.1 |
| Sternorrhyncha | Aleyrodidae | *Bemisia tabaci* | AY563655.1 |
| Sternorrhyncha | Aleyrodidae | *Bemisia tabaci* | DQ462585.1 |
| Sternorrhyncha | Aleyrodidae | *Bemisia tabaci* | EU000313.1 |
| Sternorrhyncha | Aleyrodidae | *Bemisia tabaci* | EF398094.1 |
| Sternorrhyncha | Aleyrodidae | *Bemisia tabaci* | AY563682.1 |
| Sternorrhyncha | Aleyrodidae | *Bemisia tabaci* | AY563698.1 |
| Sternorrhyncha | Aleyrodidae | *Bemisia tabaci* | AY686094 |
| Sternorrhyncha | Aleyrodidae | *Bemisia tabaci* | [EF566758.1](http://www.ncbi.nlm.nih.gov/nuccore/148575383) |
| Sternorrhyncha | Aleyrodidae | *Bemisia tabaci* | AM040609.1 |
| Sternorrhyncha | Aleyrodidae | *Bemisia tabaci* | [DQ989549.1](http://www.ncbi.nlm.nih.gov/nuccore/119352538) |
| Sternorrhyncha | Aleyrodidae | *Bemisia tabaci* | [DQ989520.1](http://www.ncbi.nlm.nih.gov/nuccore/119352480) |
| Sternorrhyncha | Aleyrodidae | *Bemisia tabaci* | [AY563669.1](http://www.ncbi.nlm.nih.gov/nuccore/58003835) |
| Sternorrhyncha | Aleyrodidae | *Bemisia tabaci* | [EF398086.1](http://www.ncbi.nlm.nih.gov/nuccore/148791842) |
| Sternorrhyncha | Aleyrodidae | *Bemisia tabaci* | AJ510061.1 |
| Sternorrhyncha | Aleyrodidae | *Bemisia tabaci* | AY057207.1 |
| Sternorrhyncha | Aleyrodidae | *Bemisia tabaci* | EU255279.1 |
| Sternorrhyncha | Aleyrodidae | *Bemisia tabaci* | [DQ116661.1](http://www.ncbi.nlm.nih.gov/nuccore/76577944) |
| Sternorrhyncha | Aleyrodidae | *Bemisia tabaci* | EF398097.1 |
| Sternorrhyncha | Aleyrodidae | *Bemisia tabaci* | AM040591.1 |
| Sternorrhyncha | Aleyrodidae | *Bemisia tabaci* | DQ309078.1 |
| Sternorrhyncha | Aleyrodidae | *Bemisia tabaci* | DQ174520.1 |
| Sternorrhyncha | Aleyrodidae | *Bemisia tabaci* | AY057178 |
| Sternorrhyncha | Aleyrodidae | *Bemisia tabaci* | AJ510057 |
| Sternorrhyncha | Aleyrodidae | *Bemisia tabaci* | AF164675.1 |
| Sternorrhyncha | Aleyrodidae | *Bemisia tabaci* | DQ989538.1 |
| Sternorrhyncha | Aleyrodidae | *Bemisia tabaci* | AY057128 |
| Sternorrhyncha | Aleyrodidae | *Bemisia tabaci* | DQ989526.1 |
| Sternorrhyncha | Aleyrodidae | *Bemisia tabaci* | EU000318.1 |
| Sternorrhyncha | Aleyrodidae | *Bemisia tabaci* | DQ174540 |
| Sternorrhyncha | Aleyrodidae | *Bemisia tabaci* | EF398092.1 |
| Sternorrhyncha | Aleyrodidae | *Bemisia tabaci* | DQ309077.1 |
| Sternorrhyncha | Aleyrodidae | *Bemisia tabaci* | AF344278 |
| Sternorrhyncha | Aleyrodidae | *Bemisia tabaci* | EU000311.1 |
| Sternorrhyncha | Aleyrodidae | *Bemisia tabaci* | AJ510070.1 |
| Sternorrhyncha | Aleyrodidae | *Bemisia tabaci* | DQ462583.1 |
| Sternorrhyncha | Aleyrodidae | *Bemisia tabaci* | EU255282.1 |
| Sternorrhyncha | Aleyrodidae | *Bemisia tabaci* | AJ550177.1 |
| Sternorrhyncha | Aleyrodidae | *Bemisia tabaci* | AY563674.1 |
| Sternorrhyncha | Aleyrodidae | *Bemisia tabaci* | AY686085 |
| Sternorrhyncha | Aleyrodidae | *Bemisia tabaci* | EF398125.1 |
| Sternorrhyncha | Aleyrodidae | *Bemisia tabaci* | AB204586.1 |
| Sternorrhyncha | Aleyrodidae | *Bemisia tabaci* | AB297897 |
| Sternorrhyncha | Aleyrodidae | *Bemisia tabaci* | AM945181.1 |
| Sternorrhyncha | Aleyrodidae | *Bemisia tabaci* | AF418668 |
| Sternorrhyncha | Aleyrodidae | *Bemisia tabaci* | AY686076.1 |
| Sternorrhyncha | Aleyrodidae | *Bemisia tabaci* | EF398082.1 |
| Sternorrhyncha | Aleyrodidae | *Bemisia tabaci* | DQ989551.1 |
| Sternorrhyncha | Aleyrodidae | *Bemisia tabaci* | AJ510074 |
| Sternorrhyncha | Aleyrodidae | *Bemisia tabaci* | AJ867556 |
| Sternorrhyncha | Aleyrodidae | *Bemisia tabaci* | DQ365877.1 |
| Sternorrhyncha | Aleyrodidae | *Bemisia tabaci* | AB204581 |
| Sternorrhyncha | Aleyrodidae | *Bemisia tabaci* | EU255284.1 |
| Sternorrhyncha | Aleyrodidae | *Bemisia tabaci* | DQ989530.1 |
| Sternorrhyncha | Aleyrodidae | *Bemisia tabaci* | AJ550173 |
| Sternorrhyncha | Aleyrodidae | *Bemisia tabaci* | AF418671.3 |
| Sternorrhyncha | Aleyrodidae | *Bemisia tabaci* | DQ174539 |
| Sternorrhyncha | Aleyrodidae | *Bemisia tabaci* | AM943062.1 |
| Sternorrhyncha | Aleyrodidae | *Bemisia tabaci* | AY686067.1 |
| Sternorrhyncha | Aleyrodidae | *Bemisia tabaci* | [AJ510068.1](http://www.ncbi.nlm.nih.gov/nuccore/46016192) |
| Sternorrhyncha | Aleyrodidae | *Bemisia tabaci* | EF667476.1 |
| Sternorrhyncha | Aleyrodidae | *Bemisia tabaci* | EU547769.1 |
| Sternorrhyncha | Aleyrodidae | *Bemisia tabaci* | AY686069.1 |
| Sternorrhyncha | Aleyrodidae | *Bemisia tabaci* | DQ989547.1 |
| Sternorrhyncha | Aleyrodidae | *Bemisia tabaci* | DQ174537.1 |
| Sternorrhyncha | Aleyrodidae | *Bemisia tabaci* | DQ116650.1 |
| Sternorrhyncha | Aleyrodidae | *Bemisia tabaci* | AB248265.1 |
| Sternorrhyncha | Aleyrodidae | *Bemisia tabaci* | EF566754.1 |
| Sternorrhyncha | Aleyrodidae | *Bemisia tabaci* | AM408896.1 |
| Sternorrhyncha | Aleyrodidae | *Bemisia tabaci* | DQ116655.1 |
| Sternorrhyncha | Aleyrodidae | *Bemisia tabaci* | AY563650.1 |
| Sternorrhyncha | Aleyrodidae | *Bemisia tabaci* | AM408899.1 |
| Sternorrhyncha | Aleyrodidae | *Bemisia tabaci* | AY686065.1 |
| Sternorrhyncha | Aleyrodidae | *Bemisia tabaci* | DQ133373.1 |
| Sternorrhyncha | Aleyrodidae | *Bemisia tabaci* | AY057170.1 |
| Sternorrhyncha | Aleyrodidae | *Bemisia tabaci* | EF566753.1 |
| Sternorrhyncha | Aleyrodidae | *Bemisia tabaci* | [EF398110.1](http://www.ncbi.nlm.nih.gov/nuccore/148791890) |
| Sternorrhyncha | Aleyrodidae | *Bemisia tabaci* | EU255287.1 |
| Sternorrhyncha | Aleyrodidae | *Bemisia tabaci* | DQ989521.1 |
| Sternorrhyncha | Aleyrodidae | *Bemisia tabaci* | EF694112.1 |
| Sternorrhyncha | Aleyrodidae | *Bemisia tabaci* | AJ510079.1 |
| Sternorrhyncha | Aleyrodidae | *Bemisia tabaci* | AB248264.1 |
| Sternorrhyncha | Aleyrodidae | *Bemisia tabaci* | AY563648.1 |
| Sternorrhyncha | Aleyrodidae | *Bemisia tabaci* | DQ365859.1 |
| Sternorrhyncha | Aleyrodidae | *Bemisia tabaci* | DQ989523.1 |
| Sternorrhyncha | Aleyrodidae | *Bemisia tabaci* | AJ510071.1 |
| Sternorrhyncha | Aleyrodidae | *Bemisia tabaci* | AM176570.1 |
| Sternorrhyncha | Aleyrodidae | *Bemisia tabaci* | EU255281.1 |
| Sternorrhyncha | Aleyrodidae | *Bemisia tabaci* | AF344245 |
| Sternorrhyncha | Aleyrodidae | *Bemisia tabaci* | AY686083 |
| Sternorrhyncha | Aleyrodidae | *Bemisia tabaci* | AY057194.1 |
| Sternorrhyncha | Aleyrodidae | *Bemisia tabaci* | AJ510072.1 |
| Sternorrhyncha | Aleyrodidae | *Bemisia tabaci* | [AY686084.1](http://www.ncbi.nlm.nih.gov/nuccore/51339252) |
| Sternorrhyncha | Aleyrodidae | *Bemisia tabaci* | AF344251 |
| Sternorrhyncha | Aleyrodidae | *Bemisia tabaci* | EF398111.1 |
| Sternorrhyncha | Aleyrodidae | *Bemisia tabaci* | AM180064.1 |
| Sternorrhyncha | Aleyrodidae | *Bemisia tabaci* | AM408895.1 |
| Sternorrhyncha | Aleyrodidae | *Bemisia tabaci* | EU376994.1 |
| Sternorrhyncha | Aleyrodidae | *Bemisia tabaci* | AJ510069.1 |
| Sternorrhyncha | Aleyrodidae | *Bemisia tabaci* | EF398096.1 |
| Sternorrhyncha | Aleyrodidae | *Bemisia tabaci* | AJ550172.1 |
| Sternorrhyncha | Aleyrodidae | *Bemisia tabaci* | EF398106.1 |
| Sternorrhyncha | Aleyrodidae | *Bemisia tabaci* | AF418665 |
| Sternorrhyncha | Aleyrodidae | *Bemisia tabaci* | EU376991.1 |
| Sternorrhyncha | Aleyrodidae | *Bemisia tabaci* | EF398084.1 |
| Sternorrhyncha | Aleyrodidae | *Bemisia tabaci* | AJ510073.1 |
| Sternorrhyncha | Aleyrodidae | *Bemisia tabaci* | AB297895.1 |
| Sternorrhyncha | Aleyrodidae | *Bemisia tabaci* | [DQ130053.1](http://www.ncbi.nlm.nih.gov/nuccore/73921501) |
| Sternorrhyncha | Aleyrodidae | *Bemisia tabaci* | AF344246 |
| Sternorrhyncha | Aleyrodidae | *Bemisia tabaci* | [AF344274.1](http://www.ncbi.nlm.nih.gov/nuccore/16565753) |
| Sternorrhyncha | Aleyrodidae | *Bemisia tabaci* | EF398105.1 |
| Sternorrhyncha | Aleyrodidae | *Bemisia tabaci* | AF418670.2 |
| Sternorrhyncha | Aleyrodidae | *Bemisia tabaci* | AJ550167 |
| Sternorrhyncha | Aleyrodidae | *Bemisia tabaci* | EU000316.1 |
| Sternorrhyncha | Aleyrodidae | *Bemisia tabaci* | AF340216.1 |
| Sternorrhyncha | Aleyrodidae | *Bemisia tabaci* | AY057196.1 |
| Sternorrhyncha | Aleyrodidae | *Bemisia tabaci* | EU376980.1 |
| Sternorrhyncha | Aleyrodidae | *Bemisia tabaci* | AJ867557 |
| Sternorrhyncha | Aleyrodidae | *Bemisia tabaci* | AJ557143.1 |
| Sternorrhyncha | Aleyrodidae | *Bemisia tabaci* | DQ130057.1 |
| Sternorrhyncha | Aleyrodidae | *Bemisia tabaci* | EF398101.1 |
| Sternorrhyncha | Aleyrodidae | *Bemisia tabaci* | DQ365873.1 |
| Sternorrhyncha | Aleyrodidae | *Bemisia tabaci* | AY057126.1 |
| Sternorrhyncha | Aleyrodidae | *Bemisia tabaci* | EF398123.1 |
| Sternorrhyncha | Aleyrodidae | *Bemisia tabaci* | AM945182.1 |
| Sternorrhyncha | Aleyrodidae | *Bemisia tabaci* | AF418667 |
| Sternorrhyncha | Aleyrodidae | *Bemisia tabaci* | DQ174530.1 |
| Sternorrhyncha | Aleyrodidae | *Bemisia tabaci* | EF080823.1 |
| Sternorrhyncha | Aleyrodidae | *Bemisia tabaci* | DQ365865.1 |
| Sternorrhyncha | Aleyrodidae | *Bemisia tabaci* | AY057162 |
| Sternorrhyncha | Aleyrodidae | *Bemisia tabaci* | EU376986.1 |
| Sternorrhyncha | Aleyrodidae | *Bemisia tabaci* | AY057198.1 |
| Sternorrhyncha | Aleyrodidae | *Bemisia tabaci* | AY563653.1 |
| Sternorrhyncha | Aleyrodidae | *Bemisia tabaci* | AY686079.1 |
| Sternorrhyncha | Aleyrodidae | *Bemisia tabaci* | AY686071.1 |
| Sternorrhyncha | Aleyrodidae | *Bemisia tabaci* | AY057208.1 |
| Sternorrhyncha | Aleyrodidae | *Bemisia tabaci* | AB204580 |
| Sternorrhyncha | Aleyrodidae | *Bemisia tabaci* | AY686073.1 |
| Sternorrhyncha | Aleyrodidae | *Bemisia tabaci* | AM696208.1 |
| Sternorrhyncha | Aleyrodidae | *Bemisia tabaci* | EU376979.1 |
| Sternorrhyncha | Aleyrodidae | *Bemisia tabaci* | AJ748394.1 |
| Sternorrhyncha | Aleyrodidae | *Bemisia tabaci* | AJ510075 |
| Sternorrhyncha | Aleyrodidae | *Bemisia tabaci* | DQ365870.1 |
| Sternorrhyncha | Aleyrodidae | *Bemisia tabaci* | AM945179.1 |
| Sternorrhyncha | Aleyrodidae | *Bemisia tabaci* | AY057174.1 |
| Sternorrhyncha | Aleyrodidae | *Bemisia tabaci* | EF694104.1 |
| Sternorrhyncha | Aleyrodidae | *Bemisia tabaci* | AJ557147.1 |
| Sternorrhyncha | Aleyrodidae | *Bemisia tabaci* | DQ174541.1 |
| Sternorrhyncha | Aleyrodidae | *Bemisia tabaci* | AY563665.1 |
| Sternorrhyncha | Aleyrodidae | *Bemisia tabaci* | EU000315.1 |
| Sternorrhyncha | Aleyrodidae | *Bemisia tabaci* | AY686089 |
| Sternorrhyncha | Aleyrodidae | *Bemisia tabaci* | AY563675.1 |
| Sternorrhyncha | Aleyrodidae | *Bemisia tabaci* | DQ116658.1 |
| Sternorrhyncha | Aleyrodidae | *Bemisia tabaci* | EF398085.1 |
| Sternorrhyncha | Aleyrodidae | *Bemisia tabaci* | AM040593.1 |
| Sternorrhyncha | Aleyrodidae | *Bemisia tabaci* | DQ174521.1 |
| Sternorrhyncha | Aleyrodidae | *Bemisia tabaci* | EF398122.1 |
| Sternorrhyncha | Aleyrodidae | *Bemisia tabaci* | DQ989531.1 |
| Sternorrhyncha | Aleyrodidae | *Bemisia tabaci* | AF342770.1 |
| Sternorrhyncha | Aleyrodidae | *Bemisia tabaci* | EU376988.1 |
| Sternorrhyncha | Aleyrodidae | *Bemisia tabaci* | AY057202.1 |
| Sternorrhyncha | Aleyrodidae | *Bemisia tabaci* | EU376976.1 |
| Sternorrhyncha | Aleyrodidae | *Bemisia tabaci* | AY563686.1 |
| Sternorrhyncha | Aleyrodidae | *Bemisia tabaci* | DQ989554.1 |
| Sternorrhyncha | Aleyrodidae | *Bemisia tabaci* | AY563646.1 |
| Sternorrhyncha | Aleyrodidae | *Bemisia tabaci* | DQ365876.1 |
| Sternorrhyncha | Aleyrodidae | *Bemisia tabaci* | EF398098.1 |
| Sternorrhyncha | Aleyrodidae | *Bemisia tabaci* | AJ550175.1 |
| Sternorrhyncha | Aleyrodidae | *Bemisia tabaci* | AB204587.1 |
| Sternorrhyncha | Aleyrodidae | *Bemisia tabaci* | EU376992.1 |
| Sternorrhyncha | Aleyrodidae | *Bemisia tabaci* | AY686092.1 |
| Sternorrhyncha | Aleyrodidae | *Bemisia tabaci* | DQ174535.1 |
| Sternorrhyncha | Aleyrodidae | *Bemisia tabaci* | AY686082.1 |
| Sternorrhyncha | Aleyrodidae | *Bemisia tabaci* | AJ510063.1 |
| Sternorrhyncha | Aleyrodidae | *Bemisia tabaci* | AB297896.1 |
| Sternorrhyncha | Aleyrodidae | *Bemisia tabaci* | AY827608.1 |
| Sternorrhyncha | Aleyrodidae | *Bemisia tabaci* | EU427721.1 |
| Sternorrhyncha | Aleyrodidae | *Bemisia tabaci* | AJ557146.1 |
| Sternorrhyncha | Aleyrodidae | *Bemisia tabaci* | AJ867555.1 |
| Sternorrhyncha | Aleyrodidae | *Bemisia tabaci* | DQ309074.1 |
| Sternorrhyncha | Aleyrodidae | *Bemisia tabaci* | DQ174538.1 |
| Sternorrhyncha | Aleyrodidae | *Bemisia tabaci* | AJ510067.1 |
| Sternorrhyncha | Aleyrodidae | *Bemisia tabaci* | AY057209.1 |
| Sternorrhyncha | Aleyrodidae | *Bemisia tabaci* | AY563652.1 |
| Sternorrhyncha | Aleyrodidae | *Bemisia tabaci* | AY057195.1 |
| Sternorrhyncha | Aleyrodidae | *Bemisia tabaci* | AY686078.1 |
| Sternorrhyncha | Aleyrodidae | *Bemisia tabaci* | AM408900.1 |
| Sternorrhyncha | Aleyrodidae | *Bemisia tabaci* | AY563643.1 |
| Sternorrhyncha | Aleyrodidae | *Bemisia tabaci* | AJ510059.1 |
| Sternorrhyncha | Aleyrodidae | *Bemisia tabaci* | AY563703.1 |
| Sternorrhyncha | Aleyrodidae | *Bemisia tabaci* | AY827588.1 |
| Sternorrhyncha | Aleyrodidae | *Bemisia tabaci* | DQ174531.1 |
| Sternorrhyncha | Aleyrodidae | *Bemisia tabaci* | AY827600.1 |
| Sternorrhyncha | Aleyrodidae | *Bemisia tabaci* | EF694107.1 |
| Sternorrhyncha | Aleyrodidae | *Bemisia tabaci* | DQ365867.1 |
| Sternorrhyncha | Aleyrodidae | *Bemisia tabaci* | AJ748395.1 |
| Sternorrhyncha | Aleyrodidae | *Bemisia tabaci* | AY827619 |
| Sternorrhyncha | Aleyrodidae | *Bemisia tabaci* | AY057149.1 |
| Sternorrhyncha | Aleyrodidae | *Bemisia tabaci* | AY563684.1 |
| Sternorrhyncha | Aleyrodidae | *Bemisia tabaci* | AJ550176.1 |
| Sternorrhyncha | Aleyrodidae | *Bemisia tabaci* | DQ116663.1 |
| Sternorrhyncha | Aleyrodidae | *Bemisia tabaci* | AM408898.1 |
| Sternorrhyncha | Aleyrodidae | *Bemisia tabaci* | EF398104.1 |
| Sternorrhyncha | Aleyrodidae | *Bemisia tabaci* | AM945180.1 |
| Sternorrhyncha | Aleyrodidae | *Bemisia tabaci* | EF398083.1 |
| Sternorrhyncha | Aleyrodidae | *Bemisia tabaci* | [EF566759.1](http://www.ncbi.nlm.nih.gov/nuccore/148575387) |
| Sternorrhyncha | Aleyrodidae | *Bemisia tabaci* | DQ365863.1 |
| Sternorrhyncha | Aleyrodidae | *Bemisia tabaci* | AB204582.1 |
| Sternorrhyncha | Aleyrodidae | *Bemisia tabaci* | AJ748396.1 |
| Sternorrhyncha | Aleyrodidae | *Bemisia tabaci* | [AY563660.1](http://www.ncbi.nlm.nih.gov/nuccore/58003821) |
| Sternorrhyncha | Aleyrodidae | *Bemisia tabaci* | AJ748373.1 |
| Sternorrhyncha | Aleyrodidae | *Bemisia tabaci* | DQ174532.1 |
| Sternorrhyncha | Aleyrodidae | *Bemisia tabaci* | DQ302946.1 |
| Sternorrhyncha | Aleyrodidae | *Bemisia tabaci* | AY057133 |
| Sternorrhyncha | Aleyrodidae | *Bemisia tabaci* | DQ989542.1 |
| Sternorrhyncha | Aleyrodidae | *Bemisia tabaci* | AY827603.1 |
| Sternorrhyncha | Aleyrodidae | *Bemisia tabaci* | AJ510077.1 |
| Sternorrhyncha | Aleyrodidae | *Bemisia tabaci* | AY057143.1 |
| Sternorrhyncha | Aleyrodidae | *Bemisia tabaci* | EU000321.1 |
| Sternorrhyncha | Aleyrodidae | *Bemisia tabaci* | AY563638.1 |
| Sternorrhyncha | Aleyrodidae | *Bemisia tabaci* | DQ989541.1 |
| Sternorrhyncha | Aleyrodidae | *Bemisia tabaci* | AY686081.1 |
| Sternorrhyncha | Aleyrodidae | *Bemisia tabaci* | AY827598.1 |
| Sternorrhyncha | Aleyrodidae | *Bemisia tabaci* | EU255278.1 |
| Sternorrhyncha | Aleyrodidae | *Bemisia tabaci* | AJ748367.1 |
| Sternorrhyncha | Aleyrodidae | *Bemisia tabaci* | AB204584.1 |
| Sternorrhyncha | Aleyrodidae | *Bemisia tabaci* | AJ748389.1 |
| Sternorrhyncha | Aleyrodidae | *Bemisia tabaci* | DQ174525.1 |
| Sternorrhyncha | Aleyrodidae | *Bemisia tabaci* | AJ510066 |
| Sternorrhyncha | Aleyrodidae | *Bemisia tabaci* | EU376975.1 |
| Sternorrhyncha | Aleyrodidae | *Bemisia tabaci* | EU376996.1 |
| Sternorrhyncha | Aleyrodidae | *Bemisia tabaci* | DQ309076.1 |
| Sternorrhyncha | Aleyrodidae | *Bemisia tabaci* | AY827590.1 |
| Sternorrhyncha | Aleyrodidae | *Bemisia tabaci* | AF418666 |
| Sternorrhyncha | Aleyrodidae | *Bemisia tabaci* | AJ783706 |
| Sternorrhyncha | Aleyrodidae | *Bemisia tabaci* | AY686093 |
| Sternorrhyncha | Aleyrodidae | *Bemisia tabaci* | AY057192.1 |
| Sternorrhyncha | Aleyrodidae | *Bemisia tabaci* | AJ748386.1 |
| Sternorrhyncha | Aleyrodidae | *Bemisia tabaci* | EF566761.1 |
| Sternorrhyncha | Aleyrodidae | *Bemisia tabaci* | AJ748375.1 |
| Sternorrhyncha | Aleyrodidae | *Bemisia tabaci* | AY057141 |
| Sternorrhyncha | Aleyrodidae | *Bemisia tabaci* | EF398113.1 |
| Sternorrhyncha | Aleyrodidae | *Bemisia tabaci* | EF398119.1 |
| Sternorrhyncha | Aleyrodidae | *Bemisia tabaci* | AY686087.1 |
| Sternorrhyncha | Aleyrodidae | *Bemisia tabaci* | AY827618.1 |
| Sternorrhyncha | Aleyrodidae | *Bemisia tabaci* | EU376990.1 |
| Sternorrhyncha | Aleyrodidae | *Bemisia tabaci* | DQ133382 |
| Sternorrhyncha | Aleyrodidae | *Bemisia tabaci* | [AB248263.1](http://www.ncbi.nlm.nih.gov/nuccore/86262109) |
| Sternorrhyncha | Aleyrodidae | *Bemisia tabaci* | DQ989539.1 |
| Sternorrhyncha | Aleyrodidae | *Bemisia tabaci* | EF694111.1 |
| Sternorrhyncha | Aleyrodidae | *Bemisia tabaci* | EF398091.1 |
| Sternorrhyncha | Aleyrodidae | *Bemisia tabaci* | EF398115.1 |
| Sternorrhyncha | Aleyrodidae | *Bemisia tabaci* | DQ462584.1 |
| Sternorrhyncha | Aleyrodidae | *Bemisia tabaci* | AJ748360.1 |
| Sternorrhyncha | Aleyrodidae | *Bemisia tabaci* | AY563702.1 |
| Sternorrhyncha | Aleyrodidae | *Bemisia tabaci* | AY563689.1 |
| Sternorrhyncha | Aleyrodidae | *Bemisia tabaci* | EU376983.1 |
| Sternorrhyncha | Aleyrodidae | *Bemisia tabaci* | AY563651.1 |
| Sternorrhyncha | Aleyrodidae | *Bemisia tabaci* | DQ365871.1 |
| Sternorrhyncha | Aleyrodidae | *Bemisia tabaci* | AB204577.1 |
| Sternorrhyncha | Aleyrodidae | *Bemisia tabaci* | EF398128.1 |
| Sternorrhyncha | Aleyrodidae | *Bemisia tabaci* | AY686088 |
| Sternorrhyncha | Aleyrodidae | *Bemisia tabaci* | [AB240967.1](http://www.ncbi.nlm.nih.gov/nuccore/81295280) |
| Sternorrhyncha | Aleyrodidae | *Bemisia tabaci* | DQ174542.1 |
| Sternorrhyncha | Aleyrodidae | *Bemisia tabaci* | AJ550168 |
| Sternorrhyncha | Aleyrodidae | *Bemisia tabaci* | [DQ989546.1](http://www.ncbi.nlm.nih.gov/nuccore/119352532) |
| Sternorrhyncha | Aleyrodidae | *Bemisia tabaci* | AF418669 |
| Sternorrhyncha | Aleyrodidae | *Bemisia tabaci* | AM040604.1 |
| Sternorrhyncha | Aleyrodidae | *Bemisia tabaci* | EF398087.1 |
| Sternorrhyncha | Aleyrodidae | *Bemisia tabaci* | AY827584.1 |
| Sternorrhyncha | Aleyrodidae | *Bemisia tabaci* | DQ130060.1 |
| Sternorrhyncha | Aleyrodidae | *Bemisia tabaci* | AF342776.1 |
| Sternorrhyncha | Aleyrodidae | *Bemisia tabaci* | AY686080.1 |
| Sternorrhyncha | Aleyrodidae | *Bemisia tabaci* | DQ989543.1 |
| Sternorrhyncha | Aleyrodidae | *Bemisia tabaci* | DQ365872.1 |
| Sternorrhyncha | Aleyrodidae | *Bemisia tabaci* | AY057197.1 |
| Sternorrhyncha | Aleyrodidae | *Bemisia tabaci* | AY057145.1 |
| Sternorrhyncha | Aleyrodidae | *Bemisia tabaci* | AB297898.1 |
| Sternorrhyncha | Aleyrodidae | *Bemisia tabaci* | AY057185.1 |
| Sternorrhyncha | Aleyrodidae | *Bemisia tabaci* | DQ989537.1 |
| Sternorrhyncha | Aleyrodidae | *Bemisia tabaci* | EF667477.1 |
| Sternorrhyncha | Aleyrodidae | *Bemisia tabaci* | AM180063 |
| Sternorrhyncha | Aleyrodidae | *Bemisia tabaci* | AY827607 |
| Sternorrhyncha | Aleyrodidae | *Bemisia tabaci* | EF398093.1 |
| Sternorrhyncha | Aleyrodidae | *Bemisia tabaci* | DQ462587.1 |
| Sternorrhyncha | Aleyrodidae | *Bemisia tabaci* | AY057179 |
| Sternorrhyncha | Aleyrodidae | *Bemisia tabaci* | DQ365857 |
| Sternorrhyncha | Aleyrodidae | *Bemisia tabaci* | AB204588.1 |
| Sternorrhyncha | Aleyrodidae | *Bemisia tabaci* | [EU376989.1](http://www.ncbi.nlm.nih.gov/nuccore/166153808) |
| Sternorrhyncha | Aleyrodidae | *Bemisia tabaci* | AY057199.1 |
| Sternorrhyncha | Aleyrodidae | *Bemisia tabaci* | AY057134 |
| Sternorrhyncha | Aleyrodidae | *Bemisia tabaci* | AM040607.1 |
| Sternorrhyncha | Aleyrodidae | *Bemisia tabaci* | EF398109.1 |
| Sternorrhyncha | Aleyrodidae | *Bemisia tabaci* | AB204579.1 |
| Sternorrhyncha | Aleyrodidae | *Bemisia tabaci* | EF566755.1 |
| Sternorrhyncha | Aleyrodidae | *Bemisia tabaci* | DQ462586.1 |
| Sternorrhyncha | Aleyrodidae | *Bemisia tabaci* | AJ550170.1 |
| Sternorrhyncha | Aleyrodidae | *Bemisia tabaci* | EF398114.1 |
| Sternorrhyncha | Aleyrodidae | *Bemisia tabaci* | EU000314.1 |
| Sternorrhyncha | Aleyrodidae | *Bemisia tabaci* | [AY563663.1](http://www.ncbi.nlm.nih.gov/nuccore/58003826) |
| Sternorrhyncha | Aleyrodidae | *Bemisia tabaci* | EU376981.1 |
| Sternorrhyncha | Aleyrodidae | *Bemisia tabaci* | AJ550182.1 |
| Sternorrhyncha | Aleyrodidae | *Bemisia tabaci* | DQ174519.1 |
| Sternorrhyncha | Aleyrodidae | *Bemisia tabaci* | EU255280.1 |
| Sternorrhyncha | Aleyrodidae | *Bemisia tabaci* | AM040606.1 |
| Sternorrhyncha | Aleyrodidae | *Bemisia tabaci* | EU376993.1 |
| Sternorrhyncha | Aleyrodidae | *Bemisia tabaci* | EU255285.1 |
| Sternorrhyncha | Aleyrodidae | *Bemisia tabaci* | DQ174534.1 |
| Sternorrhyncha | Aleyrodidae | *Bemisia tabaci* | [AM040594.1](http://www.ncbi.nlm.nih.gov/nuccore/109637815) |
| Sternorrhyncha | Aleyrodidae | *Bemisia tabaci* | AJ748391.1 |
| Sternorrhyncha | Aleyrodidae | *Bemisia tabaci* | AJ748385.1 |
| Sternorrhyncha | Aleyrodidae | *Bemisia tabaci* | DQ302947.1 |
| Sternorrhyncha | Aleyrodidae | *Bemisia tabaci* | EU000319.1 |
| Sternorrhyncha | Aleyrodidae | *Bemisia tabaci* | AY686072 |
| Sternorrhyncha | Aleyrodidae | *Bemisia tabaci* | AF342773 |
| Sternorrhyncha | Aleyrodidae | *Bemisia tabaci* | EF667475.1 |
| Sternorrhyncha | Aleyrodidae | *Bemisia tabaci* | EF398118.1 |
| Sternorrhyncha | Aleyrodidae | *Bemisia tabaci* | AM176574 |
| Sternorrhyncha | Aleyrodidae | *Bemisia tabaci* | DQ365862.1 |
| Sternorrhyncha | Aleyrodidae | *Bemisia tabaci* | DQ174528.1 |
| Sternorrhyncha | Aleyrodidae | *Bemisia tabaci* | AY563688.1 |
| Sternorrhyncha | Aleyrodidae | *Bemisia tabaci* | AM040597.1 |
| Sternorrhyncha | Aleyrodidae | *Bemisia tabaci* | DQ989536.1 |
| Sternorrhyncha | Aleyrodidae | *Bemisia tabaci* | AJ748398.1 |
| Sternorrhyncha | Aleyrodidae | *Bemisia tabaci* | DQ130052.1 |
| Sternorrhyncha | Aleyrodidae | *Bemisia tabaci* | EF398100.1 |
| Sternorrhyncha | Aleyrodidae | *Bemisia tabaci* | AM176571 |
| Sternorrhyncha | Aleyrodidae | *Bemisia tabaci* | AJ517768 |
| Sternorrhyncha | Aleyrodidae | *Bemisia tabaci* | AY563679.1 |
| Sternorrhyncha | Aleyrodidae | *Bemisia tabaci* | AY686068.1 |
| Sternorrhyncha | Aleyrodidae | *Bemisia tabaci* | DQ365860.1 |
| Sternorrhyncha | Aleyrodidae | *Bemisia tabaci* | AB248261.1 |
| Sternorrhyncha | Aleyrodidae | *Bemisia tabaci* | EF398121.1 |
| Sternorrhyncha | Aleyrodidae | *Bemisia tabaci* | EF398127.1 |
| Sternorrhyncha | Aleyrodidae | *Bemisia tabaci* | AY057216.1 |
| Sternorrhyncha | Aleyrodidae | *Bemisia tabaci* | AJ550178.1 |
| Sternorrhyncha | Aleyrodidae | *Bemisia tabaci* | EU376985.1 |
| Sternorrhyncha | Aleyrodidae | *Bemisia tabaci* | DQ116662.1 |
| Sternorrhyncha | Aleyrodidae | *Bemisia tabaci* | DQ174524.1 |
| Sternorrhyncha | Aleyrodidae | *Bemisia tabaci* | AJ784262.1 |
| Sternorrhyncha | Aleyrodidae | *Bemisia tabaci* | AF342769.1 |
| Sternorrhyncha | Aleyrodidae | *Bemisia tabaci* | DQ130062.1 |
| Sternorrhyncha | Aleyrodidae | *Bemisia tabaci* | EF398124.1 |
| Sternorrhyncha | Aleyrodidae | *Bemisia tabaci* | EU547771.1 |
| Sternorrhyncha | Aleyrodidae | *Bemisia tabaci* | DQ365868.1 |
| Sternorrhyncha | Aleyrodidae | *Bemisia tabaci* | EU427722.1 |
| Sternorrhyncha | Aleyrodidae | *Bemisia tabaci* | AM945183.1 |
| Sternorrhyncha | Aleyrodidae | *Bemisia tabaci* | AY563695.1 |
| Sternorrhyncha | Aleyrodidae | *Bemisia tabaci* | EF667473.1 |
| Sternorrhyncha | Aleyrodidae | *Bemisia tabaci* | DQ989533.1 |
| Sternorrhyncha | Aleyrodidae | *Bemisia tabaci* | [AY827602.1](http://www.ncbi.nlm.nih.gov/nuccore/57790824) |
| Sternorrhyncha | Aleyrodidae | *Bemisia tabaci* | [DQ365869.1](http://www.ncbi.nlm.nih.gov/nuccore/86560122) |
| Sternorrhyncha | Aleyrodidae | *Bemisia tabaci* | AF344276 |
| Sternorrhyncha | Aleyrodidae | *Bemisia tabaci* | AB204581.1 |
| Sternorrhyncha | Aleyrodidae | *Bemisia tabaci* | AB204580.1 |
| Sternorrhyncha | Aleyrodidae | *Bemisia tabaci* | GU086353.1 |
| Sternorrhyncha | Aleyrodidae | *Bemisia tabaci* | GU086352.1 |
| Sternorrhyncha | Aleyrodidae | *Bemisia tabaci* | GU086351.1 |
| Sternorrhyncha | Aleyrodidae | *Bemisia tabaci* | GU086350.1 |
| Sternorrhyncha | Aleyrodidae | *Bemisia tabaci* | GU086349.1 |
| Sternorrhyncha | Aleyrodidae | *Bemisia tabaci* | GU086328.1 |
| Sternorrhyncha | Aleyrodidae | *Bemisia tabaci* | GU086332.1 |
| Sternorrhyncha | Aleyrodidae | *Bemisia tabaci* | GU086327.1 |
| Sternorrhyncha | Aleyrodidae | *Bemisia tabaci* | AY057122.1 |
| Sternorrhyncha | Aleyrodidae | *Bemisia tabaci* | AY057124.1 |
| Sternorrhyncha | Aleyrodidae | *Bemisia tabaci* | AJ550168.1 |
| Sternorrhyncha | Aleyrodidae | *Bemisia tabaci* | AJ550167.1 |
| Sternorrhyncha | Aleyrodidae | *Bemisia tabaci* | EF080823.1 |
| Sternorrhyncha | Aleyrodidae | *Bemisia tabaci* | AB204578.1 |
| Sternorrhyncha | Aleyrodidae | *Bemisia tabaci* | AB204577.1 |
| Sternorrhyncha | Aleyrodidae | *Bemisia tabaci* | AJ867555.1 |
| Sternorrhyncha | Aleyrodidae | *Bemisia tabaci* | AY611642.1 |
| Sternorrhyncha | Aleyrodidae | *Bemisia tabaci* | AJ748364.1 |
| Sternorrhyncha | Aleyrodidae | *Bemisia tabaci* | AJ748361.1 |
| Sternorrhyncha | Aleyrodidae | *Bemisia tabaci* | AJ748360.1 |
| Sternorrhyncha | Aleyrodidae | *Bemisia tabaci* | AJ748359.1 |
| Sternorrhyncha | Aleyrodidae | *Bemisia tabaci* | AJ748374.1 |
| Sternorrhyncha | Aleyrodidae | *Bemisia tabaci* | AY057140.1 |
| Sternorrhyncha | Aleyrodidae | *Bemisia tabaci* | AJ748363.1 |
| Sternorrhyncha | Aleyrodidae | *Bemisia tabaci* | AY686080.1 |
| Sternorrhyncha | Aleyrodidae | *Bemisia tabaci* | AB204585.1 |
| Sternorrhyncha | Aleyrodidae | *Bemisia tabaci* | AB204584.1 |
| Sternorrhyncha | Aleyrodidae | *Bemisia tabaci* | AB204583.1 |
| Sternorrhyncha | Aleyrodidae | *Bemisia tabaci* | AB204582.1 |
| Sternorrhyncha | Aleyrodidae | *Bemisia tabaci* | AJ550178.1 |
| Sternorrhyncha | Aleyrodidae | *Bemisia tabaci* | AY827612.1 |
| Sternorrhyncha | Aleyrodidae | *Bemisia tabaci* | AJ517769.1 |
| Sternorrhyncha | Aleyrodidae | *Bemisia tabaci* | AJ550182.1 |
| Sternorrhyncha | Aleyrodidae | *Bemisia tabaci* | AJ550171.1 |
| Sternorrhyncha | Aleyrodidae | *Bemisia tabaci* | AJ550172.1 |
| Sternorrhyncha | Aleyrodidae | *Bemisia tabaci* | AJ550180.1 |
| Sternorrhyncha | Aleyrodidae | *Bemisia tabaci* | AJ550179.1 |
| Sternorrhyncha | Aleyrodidae | *Bemisia tabaci* | AB204586.1 |
| Sternorrhyncha | Aleyrodidae | *Bemisia tabaci* | AB204579.1 |
| Sternorrhyncha | Aleyrodidae | *Bemisia tabaci* | AY827615.1 |
| Sternorrhyncha | Aleyrodidae | *Bemisia tabaci* | AY827614.1 |
| Sternorrhyncha | Aleyrodidae | *Bemisia tabaci* | AY827613.1 |
| Sternorrhyncha | Aleyrodidae | *Bemisia tabaci* | AB204588.1 |
| Sternorrhyncha | Aleyrodidae | *Bemisia tabaci* | AB204587.1 |
| Sternorrhyncha | Aleyrodidae | *Bemisia tabaci* | AJ550170.1 |
| Sternorrhyncha | Aleyrodidae | *Bemisia tabaci* | AJ550169.1 |
| Sternorrhyncha | Aleyrodidae | *Bemisia tabaci* | AY766369.1 |
| Sternorrhyncha | Aleyrodidae | *Bemisia tabaci* | AF418671.3 |
| Sternorrhyncha | Aleyrodidae | *Bemisia tabaci* | AJ748375.1 |
| Sternorrhyncha | Aleyrodidae | *Bemisia tabaci* | AJ748372.1 |
| Sternorrhyncha | Aleyrodidae | *Bemisia tabaci* | AJ748367.1 |
| Sternorrhyncha | Aleyrodidae | *Bemisia tabaci* | GU086326.1 |
| Sternorrhyncha | Aleyrodidae | *Bemisia tabaci* | GU086325.1 |
| Sternorrhyncha | Aleyrodidae | *Bemisia tabaci* | EF080824.1 |
| Sternorrhyncha | Aleyrodidae | *Bemisia tabaci* | AY057123.1 |
| Sternorrhyncha | Aleyrodidae | *Bemisia tabaci* | AJ550177.1 |
| Sternorrhyncha | Aleyrodidae | *Bemisia tabaci* | AJ550176.1 |
| Sternorrhyncha | Aleyrodidae | *Bemisia tabaci* | AJ550175.1 |
| Sternorrhyncha | Aleyrodidae | *Bemisia tabaci* | AJ877264.1 |
| Sternorrhyncha | Aleyrodidae | *Bemisia tabaci* | AJ877263.1 |
| Sternorrhyncha | Aleyrodidae | *Bemisia tabaci* | EF080821.1 |
| Sternorrhyncha | Aleyrodidae | *Bemisia tabaci* | EF080822.1 |
| Sternorrhyncha | Aleyrodidae | *Bemisia tabaci* | AJ748362.1 |
| Sternorrhyncha | Aleyrodidae | *Bemisia tabaci* | AJ748358.1 |
| Sternorrhyncha | Aleyrodidae | *Bemisia tabaci* | AJ748357.1 |
| Sternorrhyncha | Aleyrodidae | *Bemisia tabaci* | AJ748378.1 |
| Sternorrhyncha | Aleyrodidae | *Bemisia tabaci* | AJ748376.1 |
| Sternorrhyncha | Aleyrodidae | *Bemisia tabaci* | AJ550174.1 |
| Sternorrhyncha | Aleyrodidae | *Bemisia tabaci* | AJ877260.1 |
| Sternorrhyncha | Aleyrodidae | *Bemisia tabaci* | AJ550173.1 |
| Sternorrhyncha | Aleyrodidae | *Bemisia tabaci* | GU086331.1 |
| Sternorrhyncha | Aleyrodidae | *Bemisia tabaci* | GU086330.1 |
| Sternorrhyncha | Aleyrodidae | *Bemisia tabaci* | GU086329.1 |
| Sternorrhyncha | Aleyrodidae | *Bemisia tabaci* | GU086333.1 |
| Sternorrhyncha | Aleyrodidae | *Bemisia tabaci* | GU086347.1 |
| Sternorrhyncha | Aleyrodidae | *Bemisia tabaci* | GU086346.1 |
| Sternorrhyncha | Aleyrodidae | *Bemisia tabaci* | AY827616.1 |
| Sternorrhyncha | Aleyrodidae | *Bemisia tabaci* | AJ748366.1 |
| Sternorrhyncha | Aleyrodidae | *Bemisia tabaci* | AJ748368.1 |
| Sternorrhyncha | Aleyrodidae | *Bemisia tabaci* | GU086342.1 |
| Sternorrhyncha | Aleyrodidae | *Bemisia tabaci* | GU086341.1 |
| Sternorrhyncha | Aleyrodidae | *Bemisia tabaci* | GU086340.1 |
| Sternorrhyncha | Aleyrodidae | *Bemisia tabaci* | AJ748371.1 |
| Sternorrhyncha | Aleyrodidae | *Bemisia tabaci* | AJ748370.1 |
| Sternorrhyncha | Aleyrodidae | *Bemisia tabaci* | AJ748369.1 |
| Sternorrhyncha | Aleyrodidae | *Bemisia tabaci* | AJ748365.1 |
| Sternorrhyncha | Aleyrodidae | *Bemisia tabaci* | AJ748373.1 |
| Sternorrhyncha | Aleyrodidae | *Bemisia tabaci* | GU086360.1 |
| Sternorrhyncha | Aleyrodidae | *Bemisia tabaci* | GU086359.1 |
| Sternorrhyncha | Aleyrodidae | *Bemisia tabaci* | GU086358.1 |
| Sternorrhyncha | Aleyrodidae | *Bemisia tabaci* | GU086348.1 |
| Sternorrhyncha | Aleyrodidae | *Bemisia tabaci* | AJ517768.1 |
| Sternorrhyncha | Aleyrodidae | *Bemisia tabaci* | AY827595.1 |
| Sternorrhyncha | Aleyrodidae | *Bemisia tabaci* | AY827597.1 |
| Sternorrhyncha | Aleyrodidae | *Bemisia tabaci* | AY827600.1 |
| Sternorrhyncha | Aleyrodidae | *Bemisia tabaci* | AY827601.1 |
| Sternorrhyncha | Aleyrodidae | *Bemisia tabaci* | AY827602.1 |
| Sternorrhyncha | Aleyrodidae | *Bemisia tabaci* | GU086354.1 |
| Sternorrhyncha | Aleyrodidae | *Bemisia tabaci* | GU086361.1 |
| Sternorrhyncha | Aleyrodidae | *Bemisia tabaci* | GU086345.1 |
| Sternorrhyncha | Aleyrodidae | *Bemisia tabaci* | GU086344.1 |
| Sternorrhyncha | Aleyrodidae | *Bemisia tabaci* | GU086343.1 |
| Sternorrhyncha | Aleyrodidae | *Bemisia tabaci* | GU086339.1 |
| Sternorrhyncha | Aleyrodidae | *Bemisia tabaci* | AY057134.1 |
| Sternorrhyncha | Aleyrodidae | *Bemisia tabaci* | AY827603.1 |
| Sternorrhyncha | Aleyrodidae | *Bemisia tabaci* | GU086338.1 |
| Sternorrhyncha | Aleyrodidae | *Bemisia tabaci* | GU086336.1 |
| Sternorrhyncha | Aleyrodidae | *Bemisia tabaci* | GU086335.1 |
| Sternorrhyncha | Aleyrodidae | *Bemisia tabaci* | GU086334.1 |
| Sternorrhyncha | Aleyrodidae | *Bemisia tabaci* | GU086337.1 |
| Sternorrhyncha | Aleyrodidae | *Bemisia tabaci* | GU086357.1 |
| Sternorrhyncha | Aleyrodidae | *Bemisia tabaci* | GU086356.1 |
| Sternorrhyncha | Aleyrodidae | *Bemisia tabaci* | GU086355.1 |
| Sternorrhyncha | Aleyrodidae | *Bemisia tabaci* | HQ703595.1 |
| Sternorrhyncha | Aleyrodidae | *Bemisia tabaci* | GU585369.1 |
| Sternorrhyncha | Aleyrodidae | *Bemisia tabaci* | GU585370.1 |
| Sternorrhyncha | Aleyrodidae | *Bemisia tabaci* | GU585372.1 |
| Sternorrhyncha | Aleyrodidae | *Bemisia tabaci* | GU585373.1 |
| Sternorrhyncha | Aleyrodidae | *Bemisia tabaci* | GU585375.1 |
| Sternorrhyncha | Aleyrodidae | *Bemisia tabaci* | GU585377.1 |
| Sternorrhyncha | Aleyrodidae | *Bemisia tabaci* | HQ198596.1 |
| Sternorrhyncha | Aleyrodidae | *Bemisia tabaci* | HQ198597.1 |
| Sternorrhyncha | Aleyrodidae | *Bemisia tabaci* | HQ198598.1 |
| Sternorrhyncha | Aleyrodidae | *Bemisia tabaci* | HQ198599.1 |
| Sternorrhyncha | Aleyrodidae | *Bemisia tabaci* | HQ198600.1 |
| Sternorrhyncha | Aleyrodidae | *Bemisia tabaci* | HQ198601.1 |
| Sternorrhyncha | Aleyrodidae | *Bemisia tabaci* | HQ198602.1 |
| Sternorrhyncha | Aleyrodidae | *Bemisia tabaci* | HQ198603.1 |
| Sternorrhyncha | Aleyrodidae | *Bemisia tabaci* | HQ198604.1 |
| Sternorrhyncha | Aleyrodidae | *Bemisia tabaci* | HQ198605.1 |
| Sternorrhyncha | Aleyrodidae | *Bemisia tabaci* | HQ198606.1 |
| Sternorrhyncha | Aleyrodidae | *Bemisia tabaci* | HQ198607.1 |
| Sternorrhyncha | Aleyrodidae | *Bemisia tabaci* | HQ198608.1 |
| Sternorrhyncha | Aleyrodidae | *Bemisia tabaci* | HQ198609.1 |
| Sternorrhyncha | Aleyrodidae | *Bemisia tabaci* | HQ198610.1 |
| Sternorrhyncha | Aleyrodidae | *Bemisia tabaci* | HQ198611.1 |
| Sternorrhyncha | Aleyrodidae | *Bemisia tabaci* | HQ198612.1 |
| Sternorrhyncha | Aleyrodidae | *Bemisia tabaci* | HQ198613.1 |
| Sternorrhyncha | Aleyrodidae | *Bemisia tabaci* | HQ198614.1 |
| Sternorrhyncha | Aleyrodidae | *Bemisia tabaci* | HQ198615.1 |
| Sternorrhyncha | Aleyrodidae | *Bemisia tabaci* | HQ198616.1 |
| Sternorrhyncha | Aleyrodidae | *Bemisia tabaci* | HQ198617.1 |
| Sternorrhyncha | Aleyrodidae | *Bemisia tabaci* | HQ198618.1 |
| Sternorrhyncha | Aleyrodidae | *Bemisia tabaci* | HQ198619.1 |
| Sternorrhyncha | Aleyrodidae | *Bemisia tabaci* | HQ198620.1 |
| Sternorrhyncha | Aleyrodidae | *Bemisia tabaci* | HQ198621.1 |
| Sternorrhyncha | Aleyrodidae | *Bemisia tabaci* | HQ198622.1 |
| Sternorrhyncha | Aleyrodidae | *Bemisia tabaci* | HQ198623.1 |
| Sternorrhyncha | Aleyrodidae | *Bemisia tabaci* | HQ198624.1 |
| Sternorrhyncha | Aleyrodidae | *Bemisia tabaci* | HQ198625.1 |
| Sternorrhyncha | Aleyrodidae | *Bemisia tabaci* | HQ198626.1 |
| Sternorrhyncha | Aleyrodidae | *Bemisia tabaci* | HQ198627.1 |
| Sternorrhyncha | Aleyrodidae | *Bemisia tabaci* | HQ198628.1 |
| Sternorrhyncha | Aleyrodidae | *Bemisia tabaci* | HQ198629.1 |
| Sternorrhyncha | Aleyrodidae | *Bemisia tabaci* | HQ198630.1 |
| Sternorrhyncha | Aleyrodidae | *Bemisia tabaci* | HQ198631.1 |
| Sternorrhyncha | Aleyrodidae | *Bemisia tabaci* | HQ198632.1 |
| Sternorrhyncha | Aleyrodidae | *Bemisia tabaci* | HQ198633.1 |
| Sternorrhyncha | Aleyrodidae | *Bemisia tabaci* | HQ198634.1 |
| Sternorrhyncha | Aleyrodidae | *Bemisia tabaci* | HQ198635.1 |
| Sternorrhyncha | Aleyrodidae | *Bemisia tabaci* | HQ198636.1 |
| Sternorrhyncha | Aleyrodidae | *Bemisia tabaci* | HQ198637.1 |
| Sternorrhyncha | Aleyrodidae | *Bemisia tabaci* | HQ198638.1 |
| Sternorrhyncha | Aleyrodidae | *Bemisia tabaci* | HQ198639.1 |
| Sternorrhyncha | Aleyrodidae | *Bemisia tabaci* | HQ198640.1 |
| Sternorrhyncha | Aleyrodidae | *Bemisia tabaci* | HQ198642.1 |
| Sternorrhyncha | Aleyrodidae | *Bemisia tabaci* | HQ198643.1 |
| Sternorrhyncha | Aleyrodidae | *Bemisia tabaci* | HQ198645.1 |
| Sternorrhyncha | Aleyrodidae | *Bemisia tabaci* | HQ198647.1 |
| Sternorrhyncha | Aleyrodidae | *Bemisia tabaci* | HQ198648.1 |
| Sternorrhyncha | Aleyrodidae | *Bemisia tabaci* | HQ198649.1 |
| Sternorrhyncha | Aleyrodidae | *Bemisia tabaci* | HQ198650.1 |
| Sternorrhyncha | Aleyrodidae | *Bemisia tabaci* | HQ198652.1 |
| Sternorrhyncha | Aleyrodidae | *Bemisia tabaci* | HQ198653.1 |
| Sternorrhyncha | Aleyrodidae | *Bemisia tabaci* | HQ198654.1 |
| Sternorrhyncha | Aleyrodidae | *Bemisia tabaci* | HQ198655.1 |
| Sternorrhyncha | Aleyrodidae | *Bemisia tabaci* | HQ198656.1 |
| Sternorrhyncha | Aleyrodidae | *Bemisia tabaci* | HQ198657.1 |
| Sternorrhyncha | Aleyrodidae | *Bemisia tabaci* | HQ198658.1 |
| Sternorrhyncha | Aleyrodidae | *Bemisia tabaci* | HQ198659.1 |
| Sternorrhyncha | Aleyrodidae | *Bemisia tabaci* | HQ198660.1 |
| Sternorrhyncha | Aleyrodidae | *Bemisia tabaci* | HQ198661.1 |
| Sternorrhyncha | Aleyrodidae | *Bemisia tabaci* | HQ198662.1 |
| Sternorrhyncha | Aleyrodidae | *Bemisia tabaci* | HQ198663.1 |
| Sternorrhyncha | Aleyrodidae | *Bemisia tabaci* | HQ198664.1 |
| Sternorrhyncha | Aleyrodidae | *Bemisia tabaci* | HQ198665.1 |
| Sternorrhyncha | Aleyrodidae | *Bemisia tabaci* | HQ198666.1 |
| Sternorrhyncha | Aleyrodidae | *Bemisia tabaci* | HQ198667.1 |
| Sternorrhyncha | Aleyrodidae | *Bemisia tabaci* | HQ198668.1 |
| Sternorrhyncha | Aleyrodidae | *Bemisia tabaci* | HQ198669.1 |
| Sternorrhyncha | Aleyrodidae | *Bemisia tabaci* | HQ198670.1 |
| Sternorrhyncha | Aleyrodidae | *Bemisia tabaci* | HQ198671.1 |
| Sternorrhyncha | Aleyrodidae | *Bemisia tabaci* | HQ198672.1 |
| Sternorrhyncha | Aleyrodidae | *Bemisia tabaci* | HQ198673.1 |
| Sternorrhyncha | Aleyrodidae | *Bemisia tabaci* | HQ198674.1 |
| Sternorrhyncha | Aleyrodidae | *Bemisia tabaci* | HQ198675.1 |
| Sternorrhyncha | Aleyrodidae | *Bemisia tabaci* | HQ198676.1 |
| Sternorrhyncha | Aleyrodidae | *Bemisia tabaci* | HQ198677.1 |
| Sternorrhyncha | Aleyrodidae | *Bemisia tabaci* | HQ198678.1 |
| Sternorrhyncha | Aleyrodidae | *Bemisia tabaci* | HQ198679.1 |
| Sternorrhyncha | Aleyrodidae | *Bemisia tabaci* | HQ198680.1 |
| Sternorrhyncha | Aleyrodidae | *Bemisia tabaci* | HQ198681.1 |
| Sternorrhyncha | Aleyrodidae | *Bemisia tabaci* | HQ198682.1 |
| Sternorrhyncha | Aleyrodidae | *Bemisia tabaci* | HQ198683.1 |
| Sternorrhyncha | Aleyrodidae | *Bemisia tabaci* | HQ198684.1 |
| Sternorrhyncha | Aleyrodidae | *Bemisia tabaci* | HQ198685.1 |
| Sternorrhyncha | Aleyrodidae | *Bemisia tabaci* | HQ198686.1 |
| Sternorrhyncha | Aleyrodidae | *Bemisia tabaci* | HQ198687.1 |
| Sternorrhyncha | Aleyrodidae | *Bemisia tabaci* | HQ198688.1 |
| Sternorrhyncha | Aleyrodidae | *Bemisia tabaci* | HQ198689.1 |
| Sternorrhyncha | Aleyrodidae | *Bemisia tabaci* | HQ198690.1 |
| Sternorrhyncha | Aleyrodidae | *Bemisia tabaci* | HQ198691.1 |
| Sternorrhyncha | Aleyrodidae | *Bemisia tabaci* | HQ198692.1 |
| Sternorrhyncha | Aleyrodidae | *Bemisia tabaci* | HQ198693.1 |
| Sternorrhyncha | Aleyrodidae | *Bemisia tabaci* | HQ198694.1 |
| Sternorrhyncha | Aleyrodidae | *Bemisia tabaci* | HQ198695.1 |
| Sternorrhyncha | Aleyrodidae | *Bemisia tabaci* | HQ198696.1 |
| Sternorrhyncha | Aleyrodidae | *Bemisia tabaci* | HQ198697.1 |
| Sternorrhyncha | Aleyrodidae | *Bemisia tabaci* | HQ198698.1 |
| Sternorrhyncha | Aleyrodidae | *Bemisia tabaci* | HQ198699.1 |
| Sternorrhyncha | Aleyrodidae | *Bemisia tabaci* | HQ198700.1 |
| Sternorrhyncha | Aleyrodidae | *Bemisia tabaci* | HQ198701.1 |
| Sternorrhyncha | Aleyrodidae | *Bemisia tabaci* | HQ198702.1 |
| Sternorrhyncha | Aleyrodidae | *Bemisia tabaci* | HQ198703.1 |
| Sternorrhyncha | Aleyrodidae | *Bemisia tabaci* | HQ198707.1 |
| Sternorrhyncha | Aleyrodidae | *Bemisia tabaci* | HQ198708.1 |
| Sternorrhyncha | Aleyrodidae | *Bemisia tabaci* | HQ198709.1 |
| Sternorrhyncha | Aleyrodidae | *Bemisia tabaci* | HQ198710.1 |
| Sternorrhyncha | Aleyrodidae | *Bemisia tabaci* | HQ198711.1 |
| Sternorrhyncha | Aleyrodidae | *Bemisia tabaci* | HQ198712.1 |
| Sternorrhyncha | Aleyrodidae | *Bemisia tabaci* | HQ198714.1 |
| Sternorrhyncha | Aleyrodidae | *Bemisia tabaci* | HQ198715.1 |
| Sternorrhyncha | Aleyrodidae | *Bemisia tabaci* | HQ198716.1 |
| Sternorrhyncha | Aleyrodidae | *Bemisia tabaci* | HQ198717.1 |
| Sternorrhyncha | Aleyrodidae | *Bemisia tabaci* | HQ198718.1 |
| Sternorrhyncha | Aleyrodidae | *Bemisia tabaci* | HQ198719.1 |
| Sternorrhyncha | Aleyrodidae | *Bemisia tabaci* | HQ198720.1 |
| Sternorrhyncha | Aleyrodidae | *Bemisia tabaci* | HQ198721.1 |
| Sternorrhyncha | Aleyrodidae | *Bemisia tabaci* | HQ198722.1 |
| Sternorrhyncha | Aleyrodidae | *Bemisia tabaci* | HQ198723.1 |
| Sternorrhyncha | Aleyrodidae | *Bemisia tabaci* | HQ198724.1 |
| Sternorrhyncha | Aleyrodidae | *Bemisia tabaci* | HQ198725.1 |
| Sternorrhyncha | Aleyrodidae | *Bemisia tabaci* | HQ198726.1 |
| Sternorrhyncha | Aleyrodidae | *Bemisia tabaci* | HQ198727.1 |
| Sternorrhyncha | Aleyrodidae | *Bemisia tabaci* | HQ198728.1 |
| Sternorrhyncha | Aleyrodidae | *Bemisia tabaci* | HQ198729.1 |
| Sternorrhyncha | Aleyrodidae | *Bemisia tabaci* | HQ198730.1 |
| Sternorrhyncha | Aleyrodidae | *Bemisia tabaci* | HQ198732.1 |
| Sternorrhyncha | Aleyrodidae | *Bemisia tabaci* | HQ198733.1 |
| Sternorrhyncha | Aleyrodidae | *Bemisia tabaci* | HQ198734.1 |
| Sternorrhyncha | Aleyrodidae | *Bemisia tabaci* | HQ198735.1 |
| Sternorrhyncha | Aleyrodidae | *Bemisia tabaci* | HQ198736.1 |
| Sternorrhyncha | Aleyrodidae | *Bemisia tabaci* | HQ198737.1 |
| Sternorrhyncha | Aleyrodidae | *Bemisia tabaci* | HQ198738.1 |
| Sternorrhyncha | Aleyrodidae | *Bemisia tabaci* | HQ198739.1 |
| Sternorrhyncha | Aleyrodidae | *Bemisia tabaci* | HQ198740.1 |
| Sternorrhyncha | Aleyrodidae | *Bemisia tabaci* | HQ198741.1 |
| Sternorrhyncha | Aleyrodidae | *Bemisia tabaci* | HQ198742.1 |
| Sternorrhyncha | Aleyrodidae | *Bemisia tabaci* | HQ198743.1 |
| Sternorrhyncha | Aleyrodidae | *Bemisia tabaci* | HQ198744.1 |
| Sternorrhyncha | Aleyrodidae | *Bemisia tabaci* | HQ198745.1 |
| Sternorrhyncha | Aleyrodidae | *Bemisia tabaci* | HQ198746.1 |
| Sternorrhyncha | Aleyrodidae | *Bemisia tabaci* | HQ198747.1 |
| Sternorrhyncha | Aleyrodidae | *Bemisia tabaci* | HQ198748.1 |
| Sternorrhyncha | Aleyrodidae | *Bemisia tabaci* | HQ198749.1 |
| Sternorrhyncha | Aleyrodidae | *Bemisia tabaci* | HQ198750.1 |
| Sternorrhyncha | Aleyrodidae | *Bemisia tabaci* | HQ198751.1 |
| Sternorrhyncha | Aleyrodidae | *Bemisia tabaci* | HQ198752.1 |
| Sternorrhyncha | Aleyrodidae | *Bemisia tabaci* | HQ198753.1 |
| Sternorrhyncha | Aleyrodidae | *Bemisia tabaci* | HQ198754.1 |
| Sternorrhyncha | Aleyrodidae | *Bemisia tabaci* | HQ198755.1 |
| Sternorrhyncha | Aleyrodidae | *Bemisia tabaci* | HQ198756.1 |
| Sternorrhyncha | Aleyrodidae | *Bemisia tabaci* | HQ198757.1 |
| Sternorrhyncha | Aleyrodidae | *Bemisia tabaci* | HQ198758.1 |
| Sternorrhyncha | Aleyrodidae | *Bemisia tabaci* | HQ198759.1 |
| Sternorrhyncha | Aleyrodidae | *Bemisia tabaci* | HQ198760.1 |
| Sternorrhyncha | Aleyrodidae | *Bemisia tabaci* | HQ198761.1 |
| Sternorrhyncha | Aleyrodidae | *Bemisia tabaci* | HQ198762.1 |
| Sternorrhyncha | Aleyrodidae | *Bemisia tabaci* | HQ198763.1 |
| Sternorrhyncha | Aleyrodidae | *Bemisia tabaci* | HQ198764.1 |
| Sternorrhyncha | Aleyrodidae | *Bemisia tabaci* | HQ198765.1 |
| Sternorrhyncha | Aleyrodidae | *Bemisia tabaci* | HQ198766.1 |
| Sternorrhyncha | Aleyrodidae | *Bemisia tabaci* | HQ198767.1 |
| Sternorrhyncha | Aleyrodidae | *Bemisia tabaci* | HQ198768.1 |
| Sternorrhyncha | Aleyrodidae | *Bemisia tabaci* | HQ198769.1 |
| Sternorrhyncha | Aleyrodidae | *Bemisia tabaci* | HQ198770.1 |
| Sternorrhyncha | Aleyrodidae | *Bemisia tabaci* | HQ198771.1 |
| Sternorrhyncha | Aleyrodidae | *Bemisia tabaci* | HQ198772.1 |
| Sternorrhyncha | Aleyrodidae | *Bemisia tabaci* | HQ198773.1 |
| Sternorrhyncha | Aleyrodidae | *Bemisia tabaci* | HQ198774.1 |
| Sternorrhyncha | Aleyrodidae | *Bemisia tabaci* | HQ198775.1 |
| Sternorrhyncha | Aleyrodidae | *Bemisia tabaci* | HQ198776.1 |
| Sternorrhyncha | Aleyrodidae | *Bemisia tabaci* | HQ198777.1 |
| Sternorrhyncha | Aleyrodidae | *Bemisia tabaci* | HQ198778.1 |
| Sternorrhyncha | Aleyrodidae | *Bemisia tabaci* | HQ198779.1 |
| Sternorrhyncha | Aleyrodidae | *Bemisia tabaci* | HQ198780.1 |
| Sternorrhyncha | Aleyrodidae | *Bemisia tabaci* | HQ198781.1 |
| Sternorrhyncha | Aleyrodidae | *Bemisia tabaci* | HQ198782.1 |
| Sternorrhyncha | Aleyrodidae | *Bemisia tabaci* | HQ198783.1 |
| Sternorrhyncha | Aleyrodidae | *Bemisia tabaci* | HQ198784.1 |
| Sternorrhyncha | Aleyrodidae | *Bemisia tabaci* | HQ198785.1 |
| Sternorrhyncha | Aleyrodidae | *Bemisia tabaci* | HQ198786.1 |
| Sternorrhyncha | Aleyrodidae | *Bemisia tabaci* | HQ198787.1 |
| Sternorrhyncha | Aleyrodidae | *Bemisia tabaci* | HQ198788.1 |
| Sternorrhyncha | Aleyrodidae | *Bemisia tabaci* | HQ198789.1 |
| Sternorrhyncha | Aleyrodidae | *Bemisia tabaci* | HQ198790.1 |
| Sternorrhyncha | Aleyrodidae | *Bemisia tabaci* | HQ198791.1 |
| Sternorrhyncha | Aleyrodidae | *Bemisia tabaci* | HQ198792.1 |
| Sternorrhyncha | Aleyrodidae | *Bemisia tabaci* | HQ198793.1 |
| Sternorrhyncha | Aleyrodidae | *Bemisia tabaci* | HQ198794.1 |
| Sternorrhyncha | Aleyrodidae | *Bemisia tabaci* | HQ198795.1 |
| Sternorrhyncha | Aleyrodidae | *Bemisia tabaci* | HQ198796.1 |
| Sternorrhyncha | Aleyrodidae | *Bemisia tabaci* | HQ198797.1 |
| Sternorrhyncha | Aleyrodidae | *Bemisia tabaci* | HQ198798.1 |
| Sternorrhyncha | Aleyrodidae | *Bemisia tabaci* | HQ198799.1 |
| Sternorrhyncha | Aleyrodidae | *Bemisia tabaci* | HQ198800.1 |
| Sternorrhyncha | Aleyrodidae | *Bemisia tabaci* | HQ198801.1 |
| Sternorrhyncha | Aleyrodidae | *Bemisia tabaci* | HQ198802.1 |
| Sternorrhyncha | Aleyrodidae | *Bemisia tabaci* | HQ198803.1 |
| Sternorrhyncha | Aleyrodidae | *Bemisia tabaci* | HQ198804.1 |
| Sternorrhyncha | Aleyrodidae | *Bemisia tabaci* | HQ198805.1 |
| Sternorrhyncha | Aleyrodidae | *Bemisia tabaci* | HQ198806.1 |
| Sternorrhyncha | Aleyrodidae | *Bemisia tabaci* | HQ198704.1 |
| Sternorrhyncha | Aleyrodidae | *Bemisia tabaci* | HQ198705.1 |
| Sternorrhyncha | Aleyrodidae | *Bemisia tabaci* | HQ198706.1 |
| Sternorrhyncha | Aleyrodidae | *Bemisia tabaci* | HQ992953.1 |
| Sternorrhyncha | Aleyrodidae | *Bemisia tabaci* | HQ992954.1 |
| Sternorrhyncha | Aleyrodidae | *Bemisia tabaci* | HQ992955.1 |
| Sternorrhyncha | Aleyrodidae | *Bemisia tabaci* | HQ992956.1 |
| Sternorrhyncha | Aleyrodidae | *Bemisia tabaci* | HQ992957.1 |
| Sternorrhyncha | Aleyrodidae | *Bemisia tabaci* | HQ992958.1 |
| Sternorrhyncha | Aleyrodidae | *Bemisia tabaci* | HQ992959.1 |
| Sternorrhyncha | Aleyrodidae | *Bemisia tabaci* | HQ992960.1 |
| Sternorrhyncha | Aleyrodidae | *Bemisia tabaci* | HQ992961.1 |
| Sternorrhyncha | Aleyrodidae | *Bemisia tabaci* | HQ992962.1 |
| Sternorrhyncha | Aleyrodidae | *Bemisia tabaci* | HQ992963.1 |
| Sternorrhyncha | Aleyrodidae | *Bemisia tabaci* | HQ621746.1 |
| Sternorrhyncha | Aleyrodidae | *Bemisia tabaci* | HQ621747.1 |
| Sternorrhyncha | Aleyrodidae | *Bemisia tabaci* | HQ621748.1 |
| Sternorrhyncha | Aleyrodidae | *Bemisia tabaci* | HQ621749.1 |
| Sternorrhyncha | Aleyrodidae | *Bemisia tabaci* | HQ621750.1 |
| Sternorrhyncha | Aleyrodidae | *Bemisia tabaci* | HQ621751.1 |
| Sternorrhyncha | Aleyrodidae | *Bemisia tabaci* | HQ621752.1 |
| Sternorrhyncha | Aleyrodidae | *Bemisia tabaci* | HQ621753.1 |
| Sternorrhyncha | Aleyrodidae | *Bemisia tabaci* | HQ621754.1 |
| Sternorrhyncha | Aleyrodidae | *Bemisia tabaci* | HQ621755.1 |
| Sternorrhyncha | Aleyrodidae | *Bemisia tabaci* | HQ621756.1 |
| Sternorrhyncha | Aleyrodidae | *Bemisia tabaci* | HQ621757.1 |
| Sternorrhyncha | Aleyrodidae | *Bemisia tabaci* | HQ621758.1 |
| Sternorrhyncha | Aleyrodidae | *Bemisia tabaci* | HQ621759.1 |
| Sternorrhyncha | Aleyrodidae | *Bemisia tabaci* | HQ198713.1 |
| Sternorrhyncha | Aleyrodidae | *Bemisia tabaci* | JF754907.1 |
| Sternorrhyncha | Aleyrodidae | *Bemisia tabaci* | JF754908.1 |
| Sternorrhyncha | Aleyrodidae | *Bemisia tabaci* | JF754909.1 |
| Sternorrhyncha | Aleyrodidae | *Bemisia tabaci* | JF754910.1 |
| Sternorrhyncha | Aleyrodidae | *Bemisia tabaci* | JF754911.1 |
| Sternorrhyncha | Aleyrodidae | *Bemisia tabaci* | JF754912.1 |
| Sternorrhyncha | Aleyrodidae | *Bemisia tabaci* | JF754913.1 |
| Sternorrhyncha | Aleyrodidae | *Bemisia tabaci* | JF754914.1 |
| Sternorrhyncha | Aleyrodidae | *Bemisia tabaci* | JF754915.1 |
| Sternorrhyncha | Aleyrodidae | *Bemisia tabaci* | JF754916.1 |
| Sternorrhyncha | Aleyrodidae | *Bemisia tabaci* | JF754917.1 |
| Sternorrhyncha | Aleyrodidae | *Bemisia tabaci* | JF754918.1 |
| Sternorrhyncha | Aleyrodidae | *Bemisia tabaci* | JF754919.1 |
| Sternorrhyncha | Aleyrodidae | *Bemisia tabaci* | JF754920.1 |
| Sternorrhyncha | Aleyrodidae | *Bemisia tabaci* | JF754921.1 |
| Sternorrhyncha | Aleyrodidae | *Bemisia tabaci* | JF754922.1 |
| Sternorrhyncha | Aleyrodidae | *Bemisia tabaci* | JF754923.1 |
| Sternorrhyncha | Aleyrodidae | *Bemisia tabaci* | JF754924.1 |
| Sternorrhyncha | Aleyrodidae | *Bemisia tabaci* | JF754925.1 |
| Sternorrhyncha | Aleyrodidae | *Bemisia tabaci* | HQ622819.1 |
| Sternorrhyncha | Aleyrodidae | *Bemisia tabaci* | HQ622820.1 |
| Sternorrhyncha | Aleyrodidae | *Bemisia tabaci* | HQ622821.1 |
| Sternorrhyncha | Aleyrodidae | *Bemisia tabaci* | HQ622822.1 |
| Sternorrhyncha | Aleyrodidae | *Bemisia tabaci* | HQ622823.1 |
| Sternorrhyncha | Aleyrodidae | *Bemisia tabaci* | HQ622824.1 |
| Sternorrhyncha | Aleyrodidae | *Bemisia tabaci* | HQ622825.1 |
| Sternorrhyncha | Aleyrodidae | *Bemisia tabaci* | HQ622826.1 |
| Sternorrhyncha | Aleyrodidae | *Bemisia tabaci* | HQ622827.1 |
| Sternorrhyncha | Aleyrodidae | *Bemisia tabaci* | HQ622828.1 |
| Sternorrhyncha | Aleyrodidae | *Bemisia tabaci* | HQ622829.1 |
| Sternorrhyncha | Aleyrodidae | *Bemisia tabaci* | HQ622830.1 |
| Sternorrhyncha | Aleyrodidae | *Bemisia tabaci* | HQ622831.1 |
| Sternorrhyncha | Aleyrodidae | *Bemisia tabaci* | HQ622832.1 |
| Sternorrhyncha | Aleyrodidae | *Bemisia tabaci* | HQ622833.1 |
| Sternorrhyncha | Aleyrodidae | *Bemisia tabaci* | HQ622834.1 |
| Sternorrhyncha | Aleyrodidae | *Bemisia tabaci* | HQ622835.1 |
| Sternorrhyncha | Aleyrodidae | *Bemisia tabaci* | HQ622836.1 |
| Sternorrhyncha | Aleyrodidae | *Bemisia tabaci* | HQ622837.1 |
| Sternorrhyncha | Aleyrodidae | *Bemisia tabaci* | HQ622838.1 |
| Sternorrhyncha | Aleyrodidae | *Bemisia tabaci* | HQ622839.1 |
| Sternorrhyncha | Aleyrodidae | *Bemisia tabaci* | HQ622840.1 |
| Sternorrhyncha | Aleyrodidae | *Bemisia tabaci* | HQ622841.1 |
| Sternorrhyncha | Aleyrodidae | *Bemisia tabaci* | HQ622842.1 |
| Sternorrhyncha | Aleyrodidae | *Bemisia tabaci* | HQ622843.1 |
| Sternorrhyncha | Aleyrodidae | *Bemisia tabaci* | HQ622844.1 |
| Sternorrhyncha | Aleyrodidae | *Bemisia tabaci* | HQ622845.1 |
| Sternorrhyncha | Aleyrodidae | *Bemisia tabaci* | HQ622846.1 |
| Sternorrhyncha | Aleyrodidae | *Bemisia tabaci* | HQ622847.1 |
| Sternorrhyncha | Aleyrodidae | *Bemisia tabaci* | HQ622848.1 |
| Sternorrhyncha | Aleyrodidae | *Bemisia tabaci* | HQ622849.1 |
| Sternorrhyncha | Aleyrodidae | *Bemisia tabaci* | HQ622850.1 |
| Sternorrhyncha | Aleyrodidae | *Bemisia tabaci* | HQ622851.1 |
| Sternorrhyncha | Aleyrodidae | *Bemisia tabaci* | HQ622852.1 |
| Sternorrhyncha | Aleyrodidae | *Bemisia tabaci* | HQ622853.1 |
| Sternorrhyncha | Aleyrodidae | *Bemisia tabaci* | HQ622854.1 |
| Sternorrhyncha | Aleyrodidae | *Bemisia tabaci* | HQ622855.1 |
| Sternorrhyncha | Aleyrodidae | *Bemisia tabaci* | HQ622856.1 |
| Sternorrhyncha | Aleyrodidae | *Bemisia tabaci* | HQ622857.1 |
| Sternorrhyncha | Aleyrodidae | *Bemisia tabaci* | HQ622858.1 |
| Sternorrhyncha | Aleyrodidae | *Bemisia tabaci* | HQ622859.1 |
| Sternorrhyncha | Aleyrodidae | *Bemisia tabaci* | HQ622860.1 |
| Sternorrhyncha | Aleyrodidae | *Bemisia tabaci* | HQ622861.1 |
| Sternorrhyncha | Aleyrodidae | *Bemisia tabaci* | HQ622862.1 |
| Sternorrhyncha | Aleyrodidae | *Bemisia tabaci* | HQ622863.1 |
| Sternorrhyncha | Aleyrodidae | *Bemisia tabaci* | HQ622864.1 |
| Sternorrhyncha | Aleyrodidae | *Bemisia tabaci* | HQ622865.1 |
| Sternorrhyncha | Aleyrodidae | *Bemisia tabaci* | HQ622866.1 |
| Sternorrhyncha | Aleyrodidae | *Bemisia tabaci* | HQ622867.1 |
| Sternorrhyncha | Aleyrodidae | *Bemisia tabaci* | HQ622868.1 |
| Sternorrhyncha | Aleyrodidae | *Bemisia tabaci* | HQ622869.1 |
| Sternorrhyncha | Aleyrodidae | *Bemisia tabaci* | HQ622870.1 |
| Sternorrhyncha | Aleyrodidae | *Bemisia tabaci* | HQ622871.1 |
| Sternorrhyncha | Aleyrodidae | *Bemisia tabaci* | HQ622872.1 |
| Sternorrhyncha | Aleyrodidae | *Bemisia tabaci* | HQ622873.1 |
| Sternorrhyncha | Aleyrodidae | *Bemisia tabaci* | HQ622874.1 |
| Sternorrhyncha | Aleyrodidae | *Bemisia tabaci* | HQ622875.1 |
| Sternorrhyncha | Aleyrodidae | *Bemisia tabaci* | HQ622876.1 |
| Sternorrhyncha | Aleyrodidae | *Bemisia tabaci* | HQ622877.1 |
| Sternorrhyncha | Aleyrodidae | *Bemisia tabaci* | HQ622878.1 |
| Sternorrhyncha | Aleyrodidae | *Bemisia tabaci* | HQ622879.1 |
| Sternorrhyncha | Aleyrodidae | *Bemisia tabaci* | HQ622880.1 |
| Sternorrhyncha | Aleyrodidae | *Bemisia tabaci* | HQ622881.1 |
| Sternorrhyncha | Aleyrodidae | *Bemisia tabaci* | HQ622882.1 |
| Sternorrhyncha | Aleyrodidae | *Bemisia tabaci* | HQ622883.1 |
| Sternorrhyncha | Aleyrodidae | *Bemisia tabaci* | HQ622884.1 |
| Sternorrhyncha | Aleyrodidae | *Bemisia tabaci* | HQ622885.1 |
| Sternorrhyncha | Aleyrodidae | *Bemisia tabaci* | HQ622886.1 |
| Sternorrhyncha | Aleyrodidae | *Bemisia tabaci* | HQ622887.1 |
| Sternorrhyncha | Aleyrodidae | *Bemisia tabaci* | HQ622888.1 |
| Sternorrhyncha | Aleyrodidae | *Bemisia tabaci* | HQ622889.1 |
| Sternorrhyncha | Aleyrodidae | *Bemisia tabaci* | HQ622890.1 |
| Sternorrhyncha | Aleyrodidae | *Bemisia tabaci* | HQ622891.1 |
| Sternorrhyncha | Aleyrodidae | *Bemisia tabaci* | HQ622892.1 |
| Sternorrhyncha | Aleyrodidae | *Bemisia tabaci* | HQ622893.1 |
| Sternorrhyncha | Aleyrodidae | *Bemisia tabaci* | HQ622894.1 |
| Sternorrhyncha | Aleyrodidae | *Bemisia tabaci* | HQ622895.1 |
| Sternorrhyncha | Aleyrodidae | *Bemisia tabaci* | HQ622896.1 |
| Sternorrhyncha | Aleyrodidae | *Bemisia tabaci* | HQ622897.1 |
| Sternorrhyncha | Aleyrodidae | *Bemisia tabaci* | HQ622898.1 |
| Sternorrhyncha | Aleyrodidae | *Bemisia tabaci* | HQ622899.1 |
| Sternorrhyncha | Aleyrodidae | *Bemisia tabaci* | HQ622900.1 |
| Sternorrhyncha | Aleyrodidae | *Bemisia tabaci* | HQ622901.1 |
| Sternorrhyncha | Aleyrodidae | *Bemisia tabaci* | HQ622902.1 |
| Sternorrhyncha | Aleyrodidae | *Bemisia tabaci* | HQ622903.1 |
| Sternorrhyncha | Aleyrodidae | *Bemisia tabaci* | GQ281714.1 |
| Sternorrhyncha | Aleyrodidae | *Bemisia tabaci* | GQ281715.1 |
| Sternorrhyncha | Aleyrodidae | *Bemisia tabaci* | GQ281716.1 |
| Sternorrhyncha | Aleyrodidae | *Bemisia tabaci* | GQ281717.1 |
| Sternorrhyncha | Aleyrodidae | *Bemisia tabaci* | GQ281718.1 |
| Sternorrhyncha | Aleyrodidae | *Bemisia tabaci* | GQ281719.1 |
| Sternorrhyncha | Aleyrodidae | *Bemisia tabaci* | GQ281720.1 |
| Sternorrhyncha | Aleyrodidae | *Bemisia tabaci* | GQ281721.1 |
| Sternorrhyncha | Aleyrodidae | *Bemisia tabaci* | GQ281722.1 |
| Sternorrhyncha | Aleyrodidae | *Bemisia tabaci* | GQ281723.1 |
| Sternorrhyncha | Aleyrodidae | *Bemisia tabaci* | GQ281724.1 |
| Sternorrhyncha | Aleyrodidae | *Bemisia tabaci* | GQ281725.1 |
| Sternorrhyncha | Aleyrodidae | *Bemisia tabaci* | GQ281726.1 |
| Sternorrhyncha | Aleyrodidae | *Bemisia tabaci* | GQ281727.1 |
| Sternorrhyncha | Aleyrodidae | *Bemisia tabaci* | GQ281728.1 |
| Sternorrhyncha | Aleyrodidae | *Bemisia tabaci* | GQ281729.1 |
| Sternorrhyncha | Aleyrodidae | *Bemisia tabaci* | GQ281730.1 |
| Sternorrhyncha | Aleyrodidae | *Bemisia tabaci* | GQ281731.1 |
| Sternorrhyncha | Aleyrodidae | *Bemisia tabaci* | GQ281732.1 |
| Sternorrhyncha | Aleyrodidae | *Bemisia tabaci* | GQ281733.1 |
| Sternorrhyncha | Aleyrodidae | *Bemisia tabaci* | GQ281734.1 |
| Sternorrhyncha | Aleyrodidae | *Bemisia tabaci* | GQ281735.1 |
| Sternorrhyncha | Aleyrodidae | *Bemisia tabaci* | GQ281736.1 |
| Sternorrhyncha | Aleyrodidae | *Bemisia tabaci* | GQ281737.1 |
| Sternorrhyncha | Aleyrodidae | *Bemisia tabaci* | GQ281738.1 |
| Sternorrhyncha | Aleyrodidae | *Bemisia tabaci* | HQ916813.1 |
| Sternorrhyncha | Aleyrodidae | *Bemisia tabaci* | HQ916814.1 |
| Sternorrhyncha | Aleyrodidae | *Bemisia tabaci* | HQ916815.1 |
| Sternorrhyncha | Aleyrodidae | *Bemisia tabaci* | HQ916816.1 |
| Sternorrhyncha | Aleyrodidae | *Bemisia tabaci* | HQ916817.1 |
| Sternorrhyncha | Aleyrodidae | *Bemisia tabaci* | HQ916818.1 |
| Sternorrhyncha | Aleyrodidae | *Bemisia tabaci* | HQ916819.1 |
| Sternorrhyncha | Aleyrodidae | *Bemisia tabaci* | HQ916820.1 |
| Sternorrhyncha | Aleyrodidae | *Bemisia tabaci* | HQ916821.1 |
| Sternorrhyncha | Aleyrodidae | *Bemisia tabaci* | HM488011.1 |
| Sternorrhyncha | Aleyrodidae | *Bemisia tabaci* | HM488018.1 |
| Sternorrhyncha | Aleyrodidae | *Bemisia tabaci* | HM488019.1 |
| Sternorrhyncha | Aleyrodidae | *Bemisia tabaci* | GU086354.1 |
| Sternorrhyncha | Aleyrodidae | *Bemisia tabaci* | GU086353.1 |
| Sternorrhyncha | Aleyrodidae | *Bemisia atriplex* | GU086363 |
| Sternorrhyncha | Aleyrodidae | *Bemisia subdecipiens* | GU220056 |
| Sternorrhyncha | Aleyrodidae | *Bemisia afer* | GU220055 |
| Archaeorrhyncha | Cixiidae | *Tachycixius pilosus* | AAO13759.1 |
| Heteroptera | Corixidae | *Sigara potamius* | ABY84606.1 |
| Heteroptera | Corixidae | *Sigara potamius* | EU176845.1 |
| Heteroptera | Corixidae | *Sigara potamius* | ABY84623.1 |
| Heteroptera | Corixidae | *Sigara potamius* | ABY84613.1 |
| Archaeorrhyncha | Delphacidae | *Anakelisia fasciata* | AAO13763.1 |
| Archaeorrhyncha | Delphacidae | *Asiraca clavicornis* | AAO13761.1 |
| Archaeorrhyncha | Delphacidae | *Chloriona glaucescens* | AAO13780.1 |
| Archaeorrhyncha | Delphacidae | *Chloriona unicolor* | AAO13769.1 |
| Archaeorrhyncha | Delphacidae | *Conomelus sp. Dijkstra-56c* | AAO13765.1 |
| Archaeorrhyncha | Delphacidae | *Javesella obscurella* | AAO13767.1 |
| Archaeorrhyncha | Delphacidae | *Javesella pellucida* | AAO13768.1 |
| Archaeorrhyncha | Delphacidae | *Kelisia sabulicola* | AAO13762.1 |
| Archaeorrhyncha | Delphacidae | *Nilaparvata lugens* | AB325705.1 |
| Archaeorrhyncha | Delphacidae | *Nilaparvata lugens* | BAF64289.1 |
| Archaeorrhyncha | Delphacidae | *Nilaparvata lugens* | AAF27934.1 |
| Archaeorrhyncha | Delphacidae | *Nilaparvata lugens* | BAF64287.1 |
| Archaeorrhyncha | Delphacidae | *Nilaparvata lugens* | AAF27935.1 |
| Archaeorrhyncha | Delphacidae | *Nilaparvata lugens* | AAF27936.1 |
| Archaeorrhyncha | Delphacidae | *Nilaparvata lugens* | AAF27937.1 |
| Archaeorrhyncha | Delphacidae | *Nilaparvata lugens* | BAF64288.1 |
| Archaeorrhyncha | Delphacidae | *Stenocranus major* | AAO13764.1 |
| Archaeorrhyncha | Delphacidae | *Ugyops* sp. Dijkstra-58 | AAO13760.1 |
| Sternorrhyncha | Eriococcidae | *Cryptococcus fagisuga* | ABA47208.1 |
| Sternorrhyncha | Eriococcidae | *Cryptococcus fagisuga* | ABA47216.1 |
| Sternorrhyncha | Eriococcidae | *Cryptococcus fagisuga* | ABA47215.1 |
| Sternorrhyncha | Eriococcidae | *Cryptococcus fagisuga* | ABA47212.1 |
| Sternorrhyncha | Eriococcidae | *Cryptococcus fagisuga* | ABA47221.1 |
| Sternorrhyncha | Eriococcidae | *Cryptococcus fagisuga* | ABA47220.1 |
| Sternorrhyncha | Eriococcidae | *Cryptococcus fagisuga* | ABA47209.1 |
| Sternorrhyncha | Eriococcidae | *Cryptococcus fagisuga* | ABA47211.1 |
| Sternorrhyncha | Eriococcidae | *Cryptococcus fagisuga* | ABA47210.1 |
| Sternorrhyncha | Eriococcidae | *Cryptococcus fagisuga* | ABA47214.1 |
| Sternorrhyncha | Eriococcidae | *Cryptococcus fagisuga* | ABA47213.1 |
| Sternorrhyncha | Eriococcidae | *Cryptococcus fagisuga* | ABA47217.1 |
| Sternorrhyncha | Eriococcidae | *Cryptococcus fagisuga* | ABA47207.1 |
| Sternorrhyncha | Eriococcidae | *Cryptococcus fagisuga* | ABA47219.1 |
| Sternorrhyncha | Eriococcidae | *Cryptococcus fagisuga* | ABA47218.1 |
| Archaeorrhyncha | Membracidae | *Procyrta pectoralis* | AAT11336.1 |
| Archaeorrhyncha | Membracidae | *Micrutalis calva* | AAT11338.1 |
| Archaeorrhyncha | Membracidae | *Acutalis* sp. CPL-2004 | AAT11340.1 |
| Archaeorrhyncha | Membracidae | *Notocera* sp. CPL-2004a | AY513402.1 |
| Archaeorrhyncha | Membracidae | *Notocera* sp. CPL-2004b | AAT11344.1 |
| Archaeorrhyncha | Membracidae | *Notocera* sp. CPL-2004c | AAT11346.1 |
| Archaeorrhyncha | Membracidae | *Notocera* sp. CPL-2004d | AAT11348.1 |
| Archaeorrhyncha | Membracidae | *Philya* sp. CPL-2004 | AAT11350.1 |
| Archaeorrhyncha | Membracidae | *Hypsoprora* sp. CPL-2004 | AAT11352.1 |
| Archaeorrhyncha | Membracidae | *Cladonota biclavata* | AAT11354.1 |
| Archaeorrhyncha | Membracidae | *Cladonota* sp. CPL-2004 | AAT11356.1 |
| Archaeorrhyncha | Membracidae | *Aconophora compressa* | AAT11358.1 |
| Archaeorrhyncha | Membracidae | *Aconophora mexicana* | AAT11360.1 |
| Sternorrhyncha | Aphididae | *Acyrthosiphon pisum* | ABD46924.1 |
| Sternorrhyncha | Aphididae | *Aphis craccivora* | AAW32102.1 |
| Sternorrhyncha | Aphididae | *Aphis glycines* | AAW32101.1 |
| Sternorrhyncha | Aphididae | *Aphis gossypii* | AAW32100.1 |
| Sternorrhyncha | Aphididae | *Cinara* aff*. apini* INHS 16456-16457 | AAP57145.1 |
| Sternorrhyncha | Aphididae | *Cinara* aff*. apini* INHS 16458 | AAP60173.1 |
| Sternorrhyncha | Aphididae | *Cinara* aff*. pinivora* CF-2003 | AAP57163.1 |
| Sternorrhyncha | Aphididae | *Cinara arizonica* | AAP57166.1 |
| Sternorrhyncha | Aphididae | *Cinara arizonica* | AAP57165.1 |
| Sternorrhyncha | Aphididae | *Cinara arizonica* | AAP57158.1 |
| Sternorrhyncha | Aphididae | *Cinara atlantica* | AAP57161.1 |
| Sternorrhyncha | Aphididae | *Cinara atlantica* | AAP57134.1 |
| Sternorrhyncha | Aphididae | *Cinara atra* | AAP57111.1 |
| Sternorrhyncha | Aphididae | *Cinara atra* | AAP57112.1 |
| Sternorrhyncha | Aphididae | *Cinara atra* | AAP57113.1 |
| Sternorrhyncha | Aphididae | *Cinara atra* | AAP57109.1 |
| Sternorrhyncha | Aphididae | *Cinara atra* | AAP60129.1 |
| Sternorrhyncha | Aphididae | *Cinara atra* | AAP57110.1 |
| Sternorrhyncha | Aphididae | *Cinara atra* | AAP57114.1 |
| Sternorrhyncha | Aphididae | *Cinara caliente* | AAP60174.1 |
| Sternorrhyncha | Aphididae | *Cinara caliente* | AAP57146.1 |
| Sternorrhyncha | Aphididae | *Cinara* cf. *hirsuta/kuchea* CF-2003 | AAP57137.1 |
| Sternorrhyncha | Aphididae | *Cinara* cf*. hirsuta/kuchea* INHS 16468 | AAP57135.1 |
| Sternorrhyncha | Aphididae | *Cinara* cf*. hirsuta/kuchea* INHS 16469-16470 | AAP57136.1 |
| Sternorrhyncha | Aphididae | *Cinara coloradensis* | AAP57155.1 |
| Sternorrhyncha | Aphididae | *Cinara contortae* | AAP57133.1 |
| Sternorrhyncha | Aphididae | *Cinara cronartii* | AAP57152.1 |
| Sternorrhyncha | Aphididae | *Cinara curvipes* | AAP57154.1 |
| Sternorrhyncha | Aphididae | *Cinara edulis* | AAP57105.1 |
| Sternorrhyncha | Aphididae | *Cinara edulis* | AAP60119.1 |
| Sternorrhyncha | Aphididae | *Cinara edulis* | AAP60130.1 |
| Sternorrhyncha | Aphididae | *Cinara edulis* | AAP57117.1 |
| Sternorrhyncha | Aphididae | *Cinara edulis* | AAP57104.1 |
| Sternorrhyncha | Aphididae | *Cinara edulis* | AAP60128.1 |
| Sternorrhyncha | Aphididae | *Cinara edulis* | AAP57107.1 |
| Sternorrhyncha | Aphididae | *Cinara edulis* | AAP60112.1 |
| Sternorrhyncha | Aphididae | *Cinara edulis* | AAP57101.1 |
| Sternorrhyncha | Aphididae | *Cinara edulis* | AAP60127.1 |
| Sternorrhyncha | Aphididae | *Cinara edulis* | AAP60115.1 |
| Sternorrhyncha | Aphididae | *Cinara edulis* | AAP60117.1 |
| Sternorrhyncha | Aphididae | *Cinara edulis* | AAP57102.1 |
| Sternorrhyncha | Aphididae | *Cinara edulis* | AAP60122.1 |
| Sternorrhyncha | Aphididae | *Cinara edulis* | AAP57106.1 |
| Sternorrhyncha | Aphididae | *Cinara edulis* | AAP60134.1 |
| Sternorrhyncha | Aphididae | *Cinara edulis* | AY300180.1 |
| Sternorrhyncha | Aphididae | *Cinara edulis* | AAP60116.1 |
| Sternorrhyncha | Aphididae | *Cinara edulis* | AAP57100.1 |
| Sternorrhyncha | Aphididae | *Cinara edulis* | AAP60131.1 |
| Sternorrhyncha | Aphididae | *Cinara edulis* | AAP60132.1 |
| Sternorrhyncha | Aphididae | *Cinara edulis* | AAP60120.1 |
| Sternorrhyncha | Aphididae | *Cinara edulis* | AAP57115.1 |
| Sternorrhyncha | Aphididae | *Cinara edulis* | AAP60137.1 |
| Sternorrhyncha | Aphididae | *Cinara edulis* | AAP57108.1 |
| Sternorrhyncha | Aphididae | *Cinara edulis* | AAP60125.1 |
| Sternorrhyncha | Aphididae | *Cinara edulis* | AAP60124.1 |
| Sternorrhyncha | Aphididae | *Cinara edulis* | AAP60114.1 |
| Sternorrhyncha | Aphididae | *Cinara edulis* | AAP60123.1 |
| Sternorrhyncha | Aphididae | *Cinara edulis* | AAP57103.1 |
| Sternorrhyncha | Aphididae | *Cinara edulis* | AAP60126.1 |
| Sternorrhyncha | Aphididae | *Cinara edulis* | AAP60135.1 |
| Sternorrhyncha | Aphididae | *Cinara edulis* | AAP60136.1 |
| Sternorrhyncha | Aphididae | *Cinara edulis* | AAP60133.1 |
| Sternorrhyncha | Aphididae | *Cinara edulis* | AAP60118.1 |
| Sternorrhyncha | Aphididae | *Cinara edulis* | AAP60113.1 |
| Sternorrhyncha | Aphididae | *Cinara edulis* | AAP60138.1 |
| Sternorrhyncha | Aphididae | *Cinara edulis* | AAP60139.1 |
| Sternorrhyncha | Aphididae | *Cinara edulis* | AAP60121.1 |
| Sternorrhyncha | Aphididae | *Cinara hottesi* | AAP57156.1 |
| Sternorrhyncha | Aphididae | *Cinara juniperivora* | AAP57157.1 |
| Sternorrhyncha | Aphididae | *Cinara murrayanae* | AAP57164.1 |
| Sternorrhyncha | Aphididae | *Cinara nigra* | AAP57153.1 |
| Sternorrhyncha | Aphididae | *Cinara nigra* | AY302056.1 |
| Sternorrhyncha | Aphididae | *Cinara pergandei* | AAP57150.1 |
| Sternorrhyncha | Aphididae | *Cinara pinivora* | AAP57162.1 |
| Sternorrhyncha | Aphididae | *Cinara ponderosae* | AAP57130.1 |
| Sternorrhyncha | Aphididae | *Cinara ponderosae* | AAP57131.1 |
| Sternorrhyncha | Aphididae | *Cinara ponderosae* | AAP57132.1 |
| Sternorrhyncha | Aphididae | *Cinara ponderosae* | AAP60152.1 |
| Sternorrhyncha | Aphididae | *Cinara ponderosae* | AAP60153.1 |
| Sternorrhyncha | Aphididae | *Cinara pseudotaxifoliae* | AAP57147.1 |
| Sternorrhyncha | Aphididae | *Cinara pseudotaxifoliae* | AAP57148.1 |
| Sternorrhyncha | Aphididae | *Cinara puerca* | AAP57149.1 |
| Sternorrhyncha | Aphididae | *Cinara schwarzii* | AAP60140.1 |
| Sternorrhyncha | Aphididae | *Cinara schwarzii* | AAP57118.1 |
| Sternorrhyncha | Aphididae | *Cinara schwarzii* | AAP60141.1 |
| Sternorrhyncha | Aphididae | *Cinara schwarzii* | AAP57119.1 |
| Sternorrhyncha | Aphididae | *Cinara strobi* | AAP57167.1 |
| Sternorrhyncha | Aphididae | *Cinara terminalis* | AAP57121.1 |
| Sternorrhyncha | Aphididae | *Cinara terminalis* | AAP60150.1 |
| Sternorrhyncha | Aphididae | *Cinara terminalis* | AAP57129.1 |
| Sternorrhyncha | Aphididae | *Cinara terminalis* | AAP60142.1 |
| Sternorrhyncha | Aphididae | *Cinara terminalis* | AAP57128.1 |
| Sternorrhyncha | Aphididae | *Cinara terminalis* | AAP60149.1 |
| Sternorrhyncha | Aphididae | *Cinara terminalis* | AAP57125.1 |
| Sternorrhyncha | Aphididae | *Cinara terminalis* | AAP57120.1 |
| Sternorrhyncha | Aphididae | *Cinara terminalis* | AAP57123.1 |
| Sternorrhyncha | Aphididae | *Cinara terminalis* | AAP60146.1 |
| Sternorrhyncha | Aphididae | *Cinara terminalis* | AAP60143.1 |
| Sternorrhyncha | Aphididae | *Cinara terminalis* | AAP60144.1 |
| Sternorrhyncha | Aphididae | *Cinara terminalis* | AAP57122.1 |
| Sternorrhyncha | Aphididae | *Cinara terminalis* | AAP60151.1 |
| Sternorrhyncha | Aphididae | *Cinara terminalis* | AAP60145.1 |
| Sternorrhyncha | Aphididae | *Cinara terminalis* | AAP57127.1 |
| Sternorrhyncha | Aphididae | *Cinara terminalis* | AAP60147.1 |
| Sternorrhyncha | Aphididae | *Cinara terminalis* | AAP57124.1 |
| Sternorrhyncha | Aphididae | *Cinara terminalis* | AAP60148.1 |
| Sternorrhyncha | Aphididae | *Cinara terminalis* | AAP57126.1 |
| Sternorrhyncha | Aphididae | *Cinara villosa* | AAP57144.1 |
| Sternorrhyncha | Aphididae | *Cinara villosa* | AAP57143.1 |
| Sternorrhyncha | Aphididae | *Cinara wahtolca* | AAP60165.1 |
| Sternorrhyncha | Aphididae | *Cinara wahtolca* | AAP60158.1 |
| Sternorrhyncha | Aphididae | *Cinara wahtolca* | AAP60168.1 |
| Sternorrhyncha | Aphididae | *Cinara wahtolca* | AAP60162.1 |
| Sternorrhyncha | Aphididae | *Cinara wahtolca* | AAP60163.1 |
| Sternorrhyncha | Aphididae | *Cinara wahtolca* | AAP57140.1 |
| Sternorrhyncha | Aphididae | *Cinara wahtolca* | AAP57139.1 |
| Sternorrhyncha | Aphididae | *Cinara wahtolca* | AAP60171.1 |
| Sternorrhyncha | Aphididae | *Cinara wahtolca* | AAP60164.1 |
| Sternorrhyncha | Aphididae | *Cinara wahtolca* | AAP60160.1 |
| Sternorrhyncha | Aphididae | *Cinara wahtolca* | AAP60157.1 |
| Sternorrhyncha | Aphididae | *Cinara wahtolca* | AAP60166.1 |
| Sternorrhyncha | Aphididae | *Cinara wahtolca* | AAP57142.1 |
| Sternorrhyncha | Aphididae | *Cinara wahtolca* | AAP60172.1 |
| Sternorrhyncha | Aphididae | *Cinara wahtolca* | AAP60154.1 |
| Sternorrhyncha | Aphididae | *Cinara wahtolca* | AAP60169.1 |
| Sternorrhyncha | Aphididae | *Cinara wahtolca* | AAP60155.1 |
| Sternorrhyncha | Aphididae | *Cinara wahtolca* | AAP60161.1 |
| Sternorrhyncha | Aphididae | *Cinara wahtolca* | AAP60159.1 |
| Sternorrhyncha | Aphididae | *Cinara wahtolca* | AAP60167.1 |
| Sternorrhyncha | Aphididae | *Cinara wahtolca* | AAP60170.1 |
| Sternorrhyncha | Aphididae | *Cinara wahtolca* | AAP57138.1 |
| Sternorrhyncha | Aphididae | *Cinara wahtolca* | AAP60156.1 |
| Sternorrhyncha | Aphididae | *Cinara wahtolca* | AAP57141.1 |
| Sternorrhyncha | Aphididae | *Cinara watsoni* | AAP57151.1 |
| Sternorrhyncha | Aphididae | *Diuraphis noxia* | AAN59929.1 |
| Sternorrhyncha | Aphididae | *Diuraphis noxia* | AAN59928.1 |
| Sternorrhyncha | Aphididae | *Epipemphigus niisimae* | ABF59799.1 |
| Sternorrhyncha | Aphididae | *Eulachnus rileyi* | AAP57160.1 |
| Sternorrhyncha | Aphididae | *Forda formicaria* | ABF59800.1 |
| Sternorrhyncha | Aphididae | *Geoica wertheimae* | ABF59801.1 |
| Sternorrhyncha | Aphididae | *Kaburagia rhusicola ensigallis* | ABF59802.1 |
| Sternorrhyncha | Aphididae | *Kaburagia rhusicola ovatirhusicola* | ABF59803.1 |
| Sternorrhyncha | Aphididae | *Kaburagia rhusicola ovogallis* | ABF59804.1 |
| Sternorrhyncha | Aphididae | *Kaburagia rhusicola rhusicola* | ABF59805.1 |
| Sternorrhyncha | Aphididae | *Longistigma caryae* | AAP57168.1 |
| Sternorrhyncha | Aphididae | *Meitanaphis elongallis* | ABF59806.1 |
| Sternorrhyncha | Aphididae | *Melaphis rhois* | AAL84752.1 |
| Sternorrhyncha | Aphididae | *Mindarus abietinus* | AAP57159.1 |
| Sternorrhyncha | Aphididae | *Pemphigus obesinymphae* | AAM21901.1 |
| Sternorrhyncha | Aphididae | *Pemphigus obesinymphae* | AAM21903.1 |
| Sternorrhyncha | Aphididae | *Pemphigus obesinymphae* | AAP40199.1 |
| Sternorrhyncha | Aphididae | *Schizaphis graminum* | AAK97044.1 |
| Sternorrhyncha | Aphididae | *Schizaphis graminum* | AAK97030.1 |
| Sternorrhyncha | Aphididae | *Schizaphis graminum* | AAK97042.1 |
| Sternorrhyncha | Aphididae | *Schizaphis graminum* | AAK97032.1 |
| Sternorrhyncha | Aphididae | *Schizaphis graminum* | AAK97040.1 |
| Sternorrhyncha | Aphididae | *Schizaphis graminum* | AAK97034.1 |
| Sternorrhyncha | Aphididae | *Schizaphis graminum* | AAK97025.1 |
| Sternorrhyncha | Aphididae | *Schizaphis graminum* | AAK97039.1 |
| Sternorrhyncha | Aphididae | *Schizaphis graminum* | AAK97028.1 |
| Sternorrhyncha | Aphididae | *Schizaphis graminum* | AAK97046.1 |
| Sternorrhyncha | Aphididae | *Schizaphis graminum* | AAK97029.1 |
| Sternorrhyncha | Aphididae | *Schizaphis graminum* | AAK97024.1 |
| Sternorrhyncha | Aphididae | *Schizaphis graminum* | AAK97036.1 |
| Sternorrhyncha | Aphididae | *Schizaphis graminum* | AAK97043.1 |
| Sternorrhyncha | Aphididae | *Schizaphis graminum* | AAK97033.1 |
| Sternorrhyncha | Aphididae | *Schizaphis graminum* | AAK97031.1 |
| Sternorrhyncha | Aphididae | *Schizaphis graminum* | AAK97026.1 |
| Sternorrhyncha | Aphididae | *Schizaphis graminum* | AAK97027.1 |
| Sternorrhyncha | Aphididae | *Schizaphis graminum* | AAK97035.1 |
| Sternorrhyncha | Aphididae | *Schizaphis graminum* | AAK97037.1 |
| Sternorrhyncha | Aphididae | *Schizaphis graminum* | AAK97045.1 |
| Sternorrhyncha | Aphididae | *Schizaphis graminum* | AAK97041.1 |
| Sternorrhyncha | Aphididae | *Schizaphis graminum* | AAK97038.1 |
| Sternorrhyncha | Aphididae | *Schizaphis graminum* | AAK97023.1 |
| Sternorrhyncha | Aphididae | *Schizaphis graminum* | AAF69689.1 |
| Sternorrhyncha | Aphididae | *Schizaphis graminum* | AAF69687.1 |
| Sternorrhyncha | Aphididae | *Schizaphis graminum* | AAF69694.1 |
| Sternorrhyncha | Aphididae | *Schizaphis graminum* | AAF69688.1 |
| Sternorrhyncha | Aphididae | *Schizaphis graminum* | AAF69691.1 |
| Sternorrhyncha | Aphididae | *Schizaphis graminum* | AAF69692.1 |
| Sternorrhyncha | Aphididae | *Schizaphis graminum* | AAF69685.1 |
| Sternorrhyncha | Aphididae | *Schizaphis graminum* | AAF69693.1 |
| Sternorrhyncha | Aphididae | *Schizaphis graminum* | AAF69686.1 |
| Sternorrhyncha | Aphididae | *Schizaphis graminum* | AAF69684.1 |
| Sternorrhyncha | Aphididae | *Schizaphis graminum* | AAF69690.1 |
| Sternorrhyncha | Aphididae | *Schizaphis graminum* | AAF69695.1 |
| Sternorrhyncha | Aphididae | *Schizaphis rotundiventris* | AAF69683.1 |
| Sternorrhyncha | Aphididae | *Schlechtendalia chinensis* | AAL84753.1 |
| Sternorrhyncha | Aphididae | *Schlechtendalia chinensis* | ABF59807.1 |
| Sternorrhyncha | Aphididae | *Schlechtendalia peitan* | ABF59808.1 |
| Sternorrhyncha | Aphididae | *Thecabius beijingensis* | ABF59809.1 |
| Sternorrhyncha | Aphididae | *Tuberculatus* sp. B Yao-2006 | ABD46928.1 |
| Sternorrhyncha | Aphididae | *Tuberculatus* sp. B Yao-2006 | ABD46927.1 |
| Sternorrhyncha | Aphididae | *Uroleucon* aff. *atripes* PHD-2007 | ABW81770.1 |
| Sternorrhyncha | Aphididae | *Uroleucon ambrosiae* | AAK21687.1 |
| Sternorrhyncha | Aphididae | *Uroleucon ambrosiae* | AAK21672.1 |
| Sternorrhyncha | Aphididae | *Uroleucon ambrosiae* | AAK21691.1 |
| Sternorrhyncha | Aphididae | *Uroleucon ambrosiae* | AAK21668.1 |
| Sternorrhyncha | Aphididae | *Uroleucon ambrosiae* | AAK21698.1 |
| Sternorrhyncha | Aphididae | *Uroleucon ambrosiae* | AAK21679.1 |
| Sternorrhyncha | Aphididae | *Uroleucon ambrosiae* | AAK21662.1 |
| Sternorrhyncha | Aphididae | *Uroleucon ambrosiae* | AAK21694.1 |
| Sternorrhyncha | Aphididae | *Uroleucon ambrosiae* | AAK21684.1 |
| Sternorrhyncha | Aphididae | *Uroleucon ambrosiae* | AAK21664.1 |
| Sternorrhyncha | Aphididae | *Uroleucon ambrosiae* | AAK21670.1 |
| Sternorrhyncha | Aphididae | *Uroleucon ambrosiae* | AAK21685.1 |
| Sternorrhyncha | Aphididae | *Uroleucon ambrosiae* | AAK21696.1 |
| Sternorrhyncha | Aphididae | *Uroleucon escalantii* | ABW81778.1 |
| Sternorrhyncha | Aphididae | *Uroleucon nigrotuberculatum* | ABW81776.1 |
| Sternorrhyncha | Aphididae | *Uroleucon pieloui* | ABW81774.1 |
| Sternorrhyncha | Aphididae | *Uroleucon reynoldense* | ABW81772.1 |
| Heteroptera | Gerridae | *Aquarius adelaidis* | AAG34286.1 |
| Heteroptera | Gerridae | *Aquarius amplus* | AAG34281.1 |
| Heteroptera | Gerridae | *Aquarius chilensis* | AAG34276.1 |
| Heteroptera | Gerridae | *Aquarius cinereus* | ABB19351.1 |
| Heteroptera | Gerridae | *Aquarius cinereus* | ABB19353.1 |
| Heteroptera | Gerridae | *Aquarius cinereus* | ABB19354.1 |
| Heteroptera | Gerridae | *Aquarius cinereus* | ABB19355.1 |
| Heteroptera | Gerridae | *Aquarius cinereus* | ABB19352.1 |
| Heteroptera | Gerridae | *Aquarius elongatus* | AAX40632.1 |
| Heteroptera | Gerridae | *Aquarius najas* | ABB19328.1 |
| Heteroptera | Gerridae | *Aquarius najas* | ABB19307.1 |
| Heteroptera | Gerridae | *Aquarius najas* | ABB19327.1 |
| Heteroptera | Gerridae | *Aquarius najas* | ABB19317.1 |
| Heteroptera | Gerridae | *Aquarius najas* | ABB19349.1 |
| Heteroptera | Gerridae | *Aquarius najas* | ABB19348.1 |
| Heteroptera | Gerridae | *Aquarius najas* | ABB19337.1 |
| Heteroptera | Gerridae | *Aquarius najas* | ABB19335.1 |
| Heteroptera | Gerridae | *Aquarius najas* | ABB19333.1 |
| Heteroptera | Gerridae | *Aquarius najas* | ABB19339.1 |
| Heteroptera | Gerridae | *Aquarius najas* | ABB19331.1 |
| Heteroptera | Gerridae | *Aquarius najas* | ABB19323.1 |
| Heteroptera | Gerridae | *Aquarius najas* | ABB19294.1 |
| Heteroptera | Gerridae | *Aquarius najas* | ABB19299.1 |
| Heteroptera | Gerridae | *Aquarius najas* | ABB19341.1 |
| Heteroptera | Gerridae | *Aquarius najas* | ABB19295.1 |
| Heteroptera | Gerridae | *Aquarius najas* | ABB19321.1 |
| Heteroptera | Gerridae | *Aquarius najas* | ABB19310.1 |
| Heteroptera | Gerridae | *Aquarius najas* | ABB19319.1 |
| Heteroptera | Gerridae | *Aquarius najas* | ABB19315.1 |
| Heteroptera | Gerridae | *Aquarius najas* | ABB19302.1 |
| Heteroptera | Gerridae | *Aquarius najas* | ABB19346.1 |
| Heteroptera | Gerridae | *Aquarius najas* | ABB19311.1 |
| Heteroptera | Gerridae | *Aquarius najas* | ABB19306.1 |
| Heteroptera | Gerridae | *Aquarius najas* | ABB19347.1 |
| Heteroptera | Gerridae | *Aquarius najas* | ABB19340.1 |
| Heteroptera | Gerridae | *Aquarius najas* | ABB19336.1 |
| Heteroptera | Gerridae | *Aquarius najas* | ABB19325.1 |
| Heteroptera | Gerridae | *Aquarius najas* | ABB19320.1 |
| Heteroptera | Gerridae | *Aquarius najas* | ABB19290.1 |
| Heteroptera | Gerridae | *Aquarius najas* | ABB19308.1 |
| Heteroptera | Gerridae | *Aquarius najas* | ABB19293.1 |
| Heteroptera | Gerridae | *Aquarius najas* | ABB19322.1 |
| Heteroptera | Gerridae | *Aquarius najas* | ABB19350.1 |
| Heteroptera | Gerridae | *Aquarius najas* | ABB19343.1 |
| Heteroptera | Gerridae | *Aquarius najas* | ABB19296.1 |
| Heteroptera | Gerridae | *Aquarius najas* | ABB19338.1 |
| Heteroptera | Gerridae | *Aquarius najas* | ABB19316.1 |
| Heteroptera | Gerridae | *Aquarius najas* | ABB19329.1 |
| Heteroptera | Gerridae | *Aquarius najas* | ABB19300.1 |
| Heteroptera | Gerridae | *Aquarius najas* | ABB19314.1 |
| Heteroptera | Gerridae | *Aquarius najas* | ABB19318.1 |
| Heteroptera | Gerridae | *Aquarius najas* | ABB19334.1 |
| Heteroptera | Gerridae | *Aquarius najas* | ABB19309.1 |
| Heteroptera | Gerridae | *Aquarius najas* | ABB19313.1 |
| Heteroptera | Gerridae | *Aquarius najas* | ABB19298.1 |
| Heteroptera | Gerridae | *Aquarius najas* | ABB19330.1 |
| Heteroptera | Gerridae | *Aquarius najas* | ABB19303.1 |
| Heteroptera | Gerridae | *Aquarius najas* | ABB19291.1 |
| Heteroptera | Gerridae | *Aquarius najas* | ABB19344.1 |
| Heteroptera | Gerridae | *Aquarius najas* | ABB19345.1 |
| Heteroptera | Gerridae | *Aquarius najas* | ABB19312.1 |
| Heteroptera | Gerridae | *Aquarius najas* | ABB19332.1 |
| Heteroptera | Gerridae | *Aquarius najas* | DQ231173.1 |
| Heteroptera | Gerridae | *Aquarius najas* | DQ231182.1 |
| Heteroptera | Gerridae | *Aquarius najas* | DQ231178.1 |
| Heteroptera | Gerridae | *Aquarius najas* | DQ231186.1 |
| Heteroptera | Gerridae | *Aquarius najas* | DQ231205.1 |
| Heteroptera | Gerridae | *Aquarius najas* | DQ231185.1 |
| Heteroptera | Gerridae | *Aquarius najas* | DQ231207.1 |
| Heteroptera | Gerridae | *Aquarius najas* | DQ231223.1 |
| Heteroptera | Gerridae | *Aquarius nyctalis* | AF200250.1 |
| Heteroptera | Gerridae | *Aquarius philippinensis* | AY336948.2 |
| Heteroptera | Gerridae | *Aquarius remigis* | U83342.1 |
| Heteroptera | Gerridae | *Aquarius ventralis* | DQ231238.1 |
| Heteroptera | Gerridae | *Aquarius ventralis* | AF200246.1 |
| Heteroptera | Gerridae | *Aquarius ventralis* | DQ231237.1 |
| Heteroptera | Gerridae | *Asclepios annandalei* | AF200282.1 |
| Heteroptera | Gerridae | *Austrobates rivularis* | AF200283.1 |
| Heteroptera | Gerridae | *Eurymetra natalensis* | AF200281.1 |
| Heteroptera | Gerridae | *Gerris argenticollis* | AF251111.1 |
| Heteroptera | Gerridae | *Gerris brasili* | ABN59270.1 |
| Heteroptera | Gerridae | *Gerris brasili* | ABN59273.1 |
| Heteroptera | Gerridae | *Gerris brasili* | ABN59275.1 |
| Heteroptera | Gerridae | *Gerris brasili* | ABN59271.1 |
| Heteroptera | Gerridae | *Gerris brasili* | ABN59274.1 |
| Heteroptera | Gerridae | *Gerris brasili* | ABN59272.1 |
| Heteroptera | Gerridae | *Gerris buenoi* | AAB66678.1 |
| Heteroptera | Gerridae | *Gerris comatus* | AAB66679.1 |
| Heteroptera | Gerridae | *Gerris costae* | ABH04935.1 |
| Heteroptera | Gerridae | *Gerris costae* | ABH04924.1 |
| Heteroptera | Gerridae | *Gerris costae* | ABH04932.1 |
| Heteroptera | Gerridae | *Gerris costae* | ABH04928.1 |
| Heteroptera | Gerridae | *Gerris costae* | ABH04923.1 |
| Heteroptera | Gerridae | *Gerris gibbifer* | ABN59263.1 |
| Heteroptera | Gerridae | *Gerris gibbifer* | ABN59258.1 |
| Heteroptera | Gerridae | *Gerris gibbifer* | ABN59260.1 |
| Heteroptera | Gerridae | *Gerris gibbifer* | ABN59256.1 |
| Heteroptera | Gerridae | *Gerris gibbifer* | ABN59264.1 |
| Heteroptera | Gerridae | *Gerris gibbifer* | ABN59268.1 |
| Heteroptera | Gerridae | *Gerris gibbifer* | ABN59265.1 |
| Heteroptera | Gerridae | *Gerris gibbifer* | ABN59266.1 |
| Heteroptera | Gerridae | *Gerris gibbifer* | ABN59259.1 |
| Heteroptera | Gerridae | *Gerris gibbifer* | ABN59269.1 |
| Heteroptera | Gerridae | *Gerris gibbifer* | ABN59255.1 |
| Heteroptera | Gerridae | *Gerris gibbifer* | ABN59267.1 |
| Heteroptera | Gerridae | *Gerris gibbifer* | ABN59261.1 |
| Heteroptera | Gerridae | *Gerris gibbifer* | ABN59262.1 |
| Heteroptera | Gerridae | *Gerris gibbifer* | ABN59257.1 |
| Heteroptera | Gerridae | *Gerris gracilicornis* | AAK29261.1 |
| Heteroptera | Gerridae | *Gerris incognitus* | AAK29265.1 |
| Heteroptera | Gerridae | *Gerris lacustris* | ABN59239.1 |
| Heteroptera | Gerridae | *Gerris lacustris* | ABN59231.1 |
| Heteroptera | Gerridae | *Gerris lacustris* | ABN59227.1 |
| Heteroptera | Gerridae | *Gerris lacustris* | ABN59249.1 |
| Heteroptera | Gerridae | *Gerris lacustris* | ABN59252.1 |
| Heteroptera | Gerridae | *Gerris lacustris* | ABN59232.1 |
| Heteroptera | Gerridae | *Gerris lacustris* | ABN59236.1 |
| Heteroptera | Gerridae | *Gerris lacustris* | ABN59234.1 |
| Heteroptera | Gerridae | *Gerris lacustris* | ABN59242.1 |
| Heteroptera | Gerridae | *Gerris lacustris* | AAG34291.1 |
| Heteroptera | Gerridae | *Gerris lacustris* | ABN59243.1 |
| Heteroptera | Gerridae | *Gerris lacustris* | ABN59223.1 |
| Heteroptera | Gerridae | *Gerris lacustris* | ABN59245.1 |
| Heteroptera | Gerridae | *Gerris lacustris* | ABN59240.1 |
| Heteroptera | Gerridae | *Gerris lacustris* | ABN59253.1 |
| Heteroptera | Gerridae | *Gerris lacustris* | ABN59228.1 |
| Heteroptera | Gerridae | *Gerris lacustris* | ABN59244.1 |
| Heteroptera | Gerridae | *Gerris lacustris* | ABN59233.1 |
| Heteroptera | Gerridae | *Gerris lacustris* | ABN59238.1 |
| Heteroptera | Gerridae | *Gerris lacustris* | ABN59226.1 |
| Heteroptera | Gerridae | *Gerris lacustris* | ABN59241.1 |
| Heteroptera | Gerridae | *Gerris lacustris* | ABN59251.1 |
| Heteroptera | Gerridae | *Gerris lacustris* | ABN59254.1 |
| Heteroptera | Gerridae | *Gerris lacustris* | ABN59224.1 |
| Heteroptera | Gerridae | *Gerris lacustris* | ABN59235.1 |
| Heteroptera | Gerridae | *Gerris lacustris* | ABN59248.1 |
| Heteroptera | Gerridae | *Gerris lacustris* | ABN59247.1 |
| Heteroptera | Gerridae | *Gerris lacustris* | ABN59246.1 |
| Heteroptera | Gerridae | *Gerris lacustris* | ABN59229.1 |
| Heteroptera | Gerridae | *Gerris lacustris* | ABN59237.1 |
| Heteroptera | Gerridae | *Gerris lacustris* | ABN59222.1 |
| Heteroptera | Gerridae | *Gerris lacustris* | ABN59225.1 |
| Heteroptera | Gerridae | *Gerris lacustris* | ABN59230.1 |
| Heteroptera | Gerridae | *Gerris lacustris* | ABN59250.1 |
| Heteroptera | Gerridae | *Gerris maculatus* | ABN59281.1 |
| Heteroptera | Gerridae | *Gerris maculatus* | ABN59282.1 |
| Heteroptera | Gerridae | *Gerris maculatus* | ABN59280.1 |
| Heteroptera | Gerridae | *Gerris maculatus* | ABN59277.1 |
| Heteroptera | Gerridae | *Gerris maculatus* | ABN59278.1 |
| Heteroptera | Gerridae | *Gerris maculatus* | ABN59279.1 |
| Heteroptera | Gerridae | *Gerris maculatus* | ABN59276.1 |
| Heteroptera | Gerridae | *Gerris pingreensis* | AAB66680.1 |
| Heteroptera | Gerridae | *Gigantometra gigas* | AAG34274.1 |
| Heteroptera | Gerridae | *Halobates alluaudi* | AAF13935.1 |
| Heteroptera | Gerridae | *Halobates bryani* | AAF13936.1 |
| Heteroptera | Gerridae | *Halobates flaviventris* | AAF13937.1 |
| Heteroptera | Gerridae | *Halobates germanus* | AAF13938.1 |
| Heteroptera | Gerridae | *Halobates hawaiiensis* | AAF13939.1 |
| Heteroptera | Gerridae | *Halobates hayanus* | AAF13940.1 |
| Heteroptera | Gerridae | *Halobates mariannarum* | AAF13949.1 |
| Heteroptera | Gerridae | *Halobates micans* | AAF13941.1 |
| Heteroptera | Gerridae | *Halobates mjobergi* | AAF13942.1 |
| Heteroptera | Gerridae | *Halobates nereis* | AAF13943.1 |
| Heteroptera | Gerridae | *Halobates poseidon* | AAF13950.1 |
| Heteroptera | Gerridae | *Halobates proavus* | AAF13951.1 |
| Heteroptera | Gerridae | *Halobates robustus* | AAF13952.1 |
| Heteroptera | Gerridae | *Halobates salotae* | AAF13953.1 |
| Heteroptera | Gerridae | *Halobates sericeus* | AAF13944.1 |
| Heteroptera | Gerridae | *Halobates sexualis* | AAF13945.1 |
| Heteroptera | Gerridae | *Halobates sobrinus* | AAF13946.1 |
| Heteroptera | Gerridae | *Halobates splendens* | AAF13947.1 |
| Heteroptera | Gerridae | *Halobates whiteleggei* | AAF13948.1 |
| Heteroptera | Gerridae | *Limnoporus canaliculatus* | AAB66681.1 |
| Heteroptera | Gerridae | *Limnoporus dissortis* | AAB66682.1 |
| Heteroptera | Gerridae | *Limnoporus dissortis* | AAB66683.1 |
| Heteroptera | Gerridae | *Limnoporus esakii* | [U83341.1](http://www.ncbi.nlm.nih.gov/nuccore/2149234) |
| Heteroptera | Gerridae | *Limnoporus genitalis* | [U83339.1](http://www.ncbi.nlm.nih.gov/nuccore/2149236) |
| Heteroptera | Gerridae | *Limnoporus notabilis* | AAB66686.1 |
| Heteroptera | Gerridae | *Limnoporus notabilis* | AAB66687.1 |
| Heteroptera | Gerridae | *Limnoporus rufoscutellatus* | AAB66689.1 |
| Heteroptera | Gerridae | *Limnoporus rufoscutellatus* | AAB66688.1 |
| Sternorrhyncha | Pseudococcidae | *Dysmicoccus brevipes* | AAM76023.1 |
| Sternorrhyncha | Pseudococcidae | *Maconellicoccus hirsutus* | AAM76029.1 |
| Sternorrhyncha | Pseudococcidae | *Melanococcus albizziae* | AAM76025.1 |
| Sternorrhyncha | Pseudococcidae | *Planococcus citri* | ABY74650.1 |
| Sternorrhyncha | Pseudococcidae | *Planococcus citri* | ABY74642.1 |
| Sternorrhyncha | Pseudococcidae | *Planococcus citri* | ABY74622.1 |
| Sternorrhyncha | Pseudococcidae | *Planococcus citri* | ABY74619.1 |
| Sternorrhyncha | Pseudococcidae | *Planococcus citri* | ABY74631.1 |
| Sternorrhyncha | Pseudococcidae | *Planococcus citri* | ABY74653.1 |
| Sternorrhyncha | Pseudococcidae | *Planococcus citri* | ABY74640.1 |
| Sternorrhyncha | Pseudococcidae | *Planococcus citri* | EU250571.1 |
| Sternorrhyncha | Pseudococcidae | *Planococcus citri* | [EU250538.1](http://www.ncbi.nlm.nih.gov/nuccore/165934505) |
| Sternorrhyncha | Pseudococcidae | *Planococcus citri* | EU250563.1 |
| Sternorrhyncha | Pseudococcidae | *Planococcus citri* | EU250541.1 |
| Sternorrhyncha | Pseudococcidae | *Planococcus citri* | EU250551.1 |
| Sternorrhyncha | Pseudococcidae | *Planococcus citri* | EU250572.1 |
| Sternorrhyncha | Pseudococcidae | *Planococcus citri* | [EU250561.1](http://www.ncbi.nlm.nih.gov/nuccore/165934551) |
| Sternorrhyncha | Pseudococcidae | *Planococcus citri* | EU250540.1 |
| Sternorrhyncha | Pseudococcidae | *Planococcus citri* | ABY74643.1 |
| Sternorrhyncha | Pseudococcidae | *Planococcus citri* | ABY74629.1 |
| Sternorrhyncha | Pseudococcidae | *Planococcus citri* | ABY74652.1 |
| Sternorrhyncha | Pseudococcidae | *Planococcus citri* | ABY74637.1 |
| Sternorrhyncha | Pseudococcidae | *Planococcus citri* | EU250566.1 |
| Sternorrhyncha | Pseudococcidae | *Planococcus citri* | ABY74645.1 |
| Sternorrhyncha | Pseudococcidae | *Planococcus citri* | ABY74620.1 |
| Sternorrhyncha | Pseudococcidae | *Planococcus citri* | ABY74651.1 |
| Sternorrhyncha | Pseudococcidae | *Planococcus citri* | ABY74641.1 |
| Sternorrhyncha | Pseudococcidae | *Planococcus citri* | ABY74628.1 |
| Sternorrhyncha | Pseudococcidae | *Planococcus citri* | EU250542.1 |
| Sternorrhyncha | Pseudococcidae | *Planococcus citri* | ABY74636.1 |
| Sternorrhyncha | Pseudococcidae | *Planococcus citri* | ABY74633.1 |
| Sternorrhyncha | Pseudococcidae | *Planococcus citri* | ABY74635.1 |
| Sternorrhyncha | Pseudococcidae | *Planococcus citri* | ABY74648.1 |
| Sternorrhyncha | Pseudococcidae | *Planococcus citri* | ABY74647.1 |
| Sternorrhyncha | Pseudococcidae | *Planococcus citri* | EU250549.1 |
| Sternorrhyncha | Pseudococcidae | *Planococcus citri* | ABY74618.1 |
| Sternorrhyncha | Pseudococcidae | *Planococcus citri* | ABY74626.1 |
| Sternorrhyncha | Pseudococcidae | *Planococcus citri* | ABY74630.1 |
| Sternorrhyncha | Pseudococcidae | *Planococcus citri* | ABY74627.1 |
| Sternorrhyncha | Pseudococcidae | *Planococcus citri* | ABY74638.1 |
| Sternorrhyncha | Pseudococcidae | *Planococcus ficus* | ABY74656.1 |
| Sternorrhyncha | Pseudococcidae | *Planococcus halli* | ABY74657.1 |
| Sternorrhyncha | Pseudococcidae | *Planococcus minor* | ABY74601.1 |
| Sternorrhyncha | Pseudococcidae | *Planococcus minor* | ABY74608.1 |
| Sternorrhyncha | Pseudococcidae | *Planococcus minor* | ABY74606.1 |
| Sternorrhyncha | Pseudococcidae | *Planococcus minor* | EU250519.1 |
| Sternorrhyncha | Pseudococcidae | *Planococcus minor* | ABY74600.1 |
| Sternorrhyncha | Pseudococcidae | *Planococcus minor* | ABY74612.1 |
| Sternorrhyncha | Pseudococcidae | *Planococcus minor* | ABY74607.1 |
| Sternorrhyncha | Pseudococcidae | *Planococcus minor* | ABY74604.1 |
| Sternorrhyncha | Pseudococcidae | *Planococcus minor* | ABY74610.1 |
| Sternorrhyncha | Pseudococcidae | *Planococcus minor* | ABY74613.1 |
| Sternorrhyncha | Pseudococcidae | *Planococcus minor* | EU250522.1 |
| Sternorrhyncha | Pseudococcidae | *Planococcus minor* | [EU250520.1](http://www.ncbi.nlm.nih.gov/nuccore/165934469) |
| Sternorrhyncha | Pseudococcidae | *Planococcus* sp. MB34 | [EU250526.1](http://www.ncbi.nlm.nih.gov/nuccore/165934481) |
| Sternorrhyncha | Pseudococcidae | *Planococcus* sp. MB44 | [EU250534.1](http://www.ncbi.nlm.nih.gov/nuccore/165934497) |
| Sternorrhyncha | Pseudococcidae | *Planococcus* sp. MB44 | [EU250531.1](http://www.ncbi.nlm.nih.gov/nuccore/165934491) |
| Sternorrhyncha | Pseudococcidae | *Planococcus* sp. MB44 | [EU250533.1](http://www.ncbi.nlm.nih.gov/nuccore/165934495) |
| Sternorrhyncha | Pseudococcidae | *Planococcus* sp. MB4*9* | [EU250532.1](http://www.ncbi.nlm.nih.gov/nuccore/165934493) |
| Sternorrhyncha | Pseudococcidae | *Planococcus* sp. MB58 | [EU250528.1](http://www.ncbi.nlm.nih.gov/nuccore/165934485) |
| Sternorrhyncha | Pseudococcidae | *Planococcus* sp. MB59 | [EU250556.1](http://www.ncbi.nlm.nih.gov/nuccore/165934541) |
| Sternorrhyncha | Pseudococcidae | *Pseudococcus longispinus* | [DQ238222.1](http://www.ncbi.nlm.nih.gov/nuccore/78369515) |
