# Supplementary material for: Taxonomic Status of the Bemisia tabaci Complex (Hemiptera: Aleyrodidae) and Reassessment of the Number of Its Constituent Species
Source: PLoS One. 2013 May 13;8(5):e63817. doi: 10.1371/journal.pone.0063817 (PMC3652838; doi:10.1371/journal.pone.0063817)
Supplement: Table S5 — The list of 47 individuals of 34 aphid species. (DOC) [file pone.0063817.s005.doc]

**Table S5 The list of 47 individuals of 34 aphid species.**

| **Species** | **Accession number** |
| --- | --- |
| *Aploneura lentisci* | AY227083.1 |
| *Asiphonella dactylonii* | AY227084.1 |
| *Epipemphigus niisimae* | DQ499620.1 |
| *Epipemphigus niisimae* | DQ779160.1 |
| *Epipemphigus niisimae* | DQ779161.1 |
| *Eriosoma lanigerum* | EF534360.1 |
| *Eriosoma ulmi* | EF534362.1 |
| *Floraphis choui* | FJ589215.1 |
| *Floraphis meitanensis* | FJ589216.1 |
| *Forda formicaria* | AY227086.1 |
| *Forda marginata* | AY227091.1 |
| *Formosaphis micheliae* | DQ779162.1 |
| *Geoica wertheimae* | DQ499622.1 |
| *Kaburagia rhusicola* | DQ499623.1 |
| *Kaburagia rhusicola* | DQ499624.1 |
| *Kaburagia rhusicola* | DQ499625.1 |
| *Kaburagia rhusicola* | DQ499626.1 |
| *Meitanaphis elongallis* | FJ589217.1 |
| *Meitanaphis elongallis* | DQ499627.1 |
| *Meitanaphis flavogallis* | FJ589220.1 |
| *Meitanaphis microgallis* | FJ589221.1 |
| *Melaphis rhois* | AF469804.1 |
| *Nurudea shiraii* | FJ589225.1 |
| *Nurudea yanoniella* | FJ589222.1 |
| *Paracletus cimiciformis* | AY227089.1 |
| *Pemphigus borealis* | DQ779163.1 |
| *Pemphigus bursarius* | DQ779164.1 |
| *Pemphigus immunis* | DQ779165.1 |
| *Pemphigus matsumurai* | DQ779166.1 |
| *Pemphigus matsumurai* | DQ779167.1 |
| *Pemphigus monophagus* | DQ779168.1 |
| *Pemphigus mordwilkoi* | DQ779169.1 |
| *Pemphigus populi-transversus* | DQ779170.1 |
| *Pemphigus tibetensis* | DQ779171.1 |
| *Prociphilus pini* | DQ779172.1 |
| *Schlechtendalia chinensis* | AF469805.1 |
| *Schlechtendalia chinensis* | FJ589226.1 |
| *Schlechtendalia chinensis* | DQ499628.1 |
| *Schlechtendalia peitan* | DQ499629.1 |
| *Tetraneura akinire* | EF534363.1 |
| *Tetraneura chinensis* | EF534365.1 |
| *Tetraneura chinensis* | EF534366.1 |
| *Tetraneura chinensis* | EF534367.1 |
| *Tetraneura chinensis* | EF534368.1 |
| *Tetraneura sorini* | EF534364.1 |
| *Thecabius beijingensis* | DQ499630.1 |
| *Thecabius beijingensis* | DQ779173.1 |
