# Supplementary material for: Taxonomic Status of the Bemisia tabaci Complex (Hemiptera: Aleyrodidae) and Reassessment of the Number of Its Constituent Species
Source: PLoS One. 2013 May 13;8(5):e63817. doi: 10.1371/journal.pone.0063817 (PMC3652838; doi:10.1371/journal.pone.0063817)
Supplement: Table S6 — The list of 20 hemipterea mitochondrial genomes. (DOC) [file pone.0063817.s006.doc]

**Table S6** The list of 20 hemipterea mitochondrial genomes.

| **Suborder** | **Family** | **Species** | **Accession number** |
| --- | --- | --- | --- |
| [Auchenorrhyncha](http://www.dropdata.org/entomology/Hemiptera2_s.pdf) | [Aphrophoridae](http://www.ncbi.nlm.nih.gov/Taxonomy/Browser/wwwtax.cgi?mode=Undef&id=36666&lvl=3&keep=1&srchmode=1&unlock) | *Philaenus spumarius* | NC 005944 |
| [Heteroptera](http://www.ncbi.nlm.nih.gov/Taxonomy/Browser/wwwtax.cgi?mode=Undef&id=33345&lvl=3&keep=1&srchmode=1&unlock) | [Reduviidae](http://www.ncbi.nlm.nih.gov/Taxonomy/Browser/wwwtax.cgi?mode=Undef&id=27479&lvl=3&keep=1&srchmode=1&unlock) | *Triatoma dimidiata* | NC 002609 |
| [Heteroptera](http://www.ncbi.nlm.nih.gov/Taxonomy/Browser/wwwtax.cgi?mode=Undef&id=33345&lvl=3&keep=1&srchmode=1&unlock) | [Pentatomidae](http://www.ncbi.nlm.nih.gov/Taxonomy/Browser/wwwtax.cgi?mode=Undef&id=160513&lvl=3&keep=1&srchmode=1&unlock) | *Nezara viridula* | NC 011755 |
| [Heteroptera](http://www.ncbi.nlm.nih.gov/Taxonomy/Browser/wwwtax.cgi?mode=Undef&id=33345&lvl=3&keep=1&srchmode=1&unlock) | [Plataspidae](http://www.ncbi.nlm.nih.gov/Taxonomy/Browser/wwwtax.cgi?mode=Undef&id=236385&lvl=3&keep=1&srchmode=1&unlock) | *Coptosoma bifaria* | EU427334 |
| [Heteroptera](http://www.ncbi.nlm.nih.gov/Taxonomy/Browser/wwwtax.cgi?mode=Undef&id=33345&lvl=3&keep=1&srchmode=1&unlock) | [Berytidae](http://www.ncbi.nlm.nih.gov/Taxonomy/Browser/wwwtax.cgi?mode=Undef&id=236388&lvl=3&keep=1&srchmode=1&unlock) | *Yemmalysus parallelus* | EU427346 |
| [Heteroptera](http://www.ncbi.nlm.nih.gov/Taxonomy/Browser/wwwtax.cgi?mode=Undef&id=33345&lvl=3&keep=1&srchmode=1&unlock) | [Malcidae](http://www.ncbi.nlm.nih.gov/Taxonomy/Browser/wwwtax.cgi?mode=Undef&id=236391&lvl=3&keep=1&srchmode=1&unlock) | *Malcus inconspicuus* | EU427339 |
| Heteroptera | *Coreidae* | *Hydaropsis longirostris* | EU427337 |
| Heteroptera | [Alydidae](http://www.ncbi.nlm.nih.gov/Taxonomy/Browser/wwwtax.cgi?mode=Undef&id=41702&lvl=3&keep=1&srchmode=1&unlock) | *Riptortus pedestris* | EU427344 |
| Heteroptera | [Pyrrhocoridae](http://www.ncbi.nlm.nih.gov/Taxonomy/Browser/wwwtax.cgi?mode=Undef&id=36998&lvl=3&keep=1&srchmode=1&unlock) | *Dysdercus cingulatus* | EU427335 |
| Heteroptera | [Largidae](http://www.ncbi.nlm.nih.gov/Taxonomy/Browser/wwwtax.cgi?mode=Undef&id=209984&lvl=3&keep=1&srchmode=1&unlock) | *Physopelta gutta* | EU427343 |
| Heteroptera | [Saldidae](http://www.ncbi.nlm.nih.gov/Taxonomy/Browser/wwwtax.cgi?mode=Undef&id=50654&lvl=3&keep=1&srchmode=1&unlock) | *Saldula arsenjevi* | EU427345 |
| Heteroptera | [Aradidae](http://www.ncbi.nlm.nih.gov/Taxonomy/Browser/wwwtax.cgi?mode=Undef&id=236322&lvl=3&keep=1&srchmode=1&unlock) | *Neuroctenus parus* | EU427340 |
| Sternorrhyncha | [Psyllidae](http://www.ncbi.nlm.nih.gov/Taxonomy/Browser/wwwtax.cgi?mode=Undef&id=30092&lvl=3&keep=1&srchmode=1&unlock) | *Pachypsylla venusta* | NC 006157 |
| Sternorrhyncha | *Aphididae* | *Schizaphis graminum* | NC 006158 |
| Sternorrhyncha | [Aleyrodidae](http://www.ncbi.nlm.nih.gov/Taxonomy/Browser/wwwtax.cgi?mode=Undef&id=7036&lvl=3&keep=1&srchmode=1&unlock) | *Aleurochiton aceris* | NC 006160 |
| Sternorrhyncha | [Aleyrodidae](http://www.ncbi.nlm.nih.gov/Taxonomy/Browser/wwwtax.cgi?mode=Undef&id=7036&lvl=3&keep=1&srchmode=1&unlock) | *Bemisia tabaci* | NC 006279 |
| Sternorrhyncha | [Aleyrodidae](http://www.ncbi.nlm.nih.gov/Taxonomy/Browser/wwwtax.cgi?mode=Undef&id=7036&lvl=3&keep=1&srchmode=1&unlock) | *Trialeurodes vaporariorum* | NC 006280 |
| Sternorrhyncha | [Aleyrodidae](http://www.ncbi.nlm.nih.gov/Taxonomy/Browser/wwwtax.cgi?mode=Undef&id=7036&lvl=3&keep=1&srchmode=1&unlock) | *Neomaskellia andropogonis* | NC 006159 |
| Sternorrhyncha | [Aleyrodidae](http://www.ncbi.nlm.nih.gov/Taxonomy/Browser/wwwtax.cgi?mode=Undef&id=7036&lvl=3&keep=1&srchmode=1&unlock) | *Tetraleurodes acaciae* | NC 006292 |
| Sternorrhyncha | [Aleyrodidae](http://www.ncbi.nlm.nih.gov/Taxonomy/Browser/wwwtax.cgi?mode=Undef&id=7036&lvl=3&keep=1&srchmode=1&unlock) | *Aleurodicus dugesii* | NC 005939 |
